# Efficacy and Safety of Fixed-Dose Artesunate-Amodiaquine vs. Artemether-Lumefantrine for Repeated Treatment of Uncomplicated Malaria in Ugandan Children

**DOI:** 10.1371/journal.pone.0113311

**Published:** 2014-12-01

**Authors:** Adoke Yeka, Valerie Lameyre, Kibuuka Afizi, Mudangha Fredrick, Robinson Lukwago, Moses R. Kamya, Ambrose O. Talisuna

**Affiliations:** 1 School of Public Health, College of Health Sciences, Makerere University, Kampala, Uganda; 2 Sanofi Access to Medicines, Gentilly, France; 3 Department of Medicine, Makerere University College of Health Sciences, Kampala, Uganda; 4 Uganda Malaria Surveillance Program, Kampala, Uganda; 5 Malaria Public Health Department, University of Oxford-KEMRI-Wellcome Trust Programme, Nairobi, Kenya; The George Washington University Medical Center, United States of America

## Abstract

The safety and efficacy of the two most widely used fixed-dose artemisinin-based combination therapies (ACT), artesunate-amodiaquine (ASAQ) and artemether-lumefantrine (AL) are well established for single episodes of uncomplicated *Plasmodium falciparum* malaria, but the effects of repeated, long-term use are not well documented. We conducted a 2-year randomized, open-label, longitudinal, phase IV clinical trial comparing the efficacy and safety of fixed-dose ASAQ and AL for repeated treatment of uncomplicated malaria in children under 5 years at Nagongera Health Centre, Uganda. Participants were randomized to ASAQ or AL and all subsequent malaria episodes were treated with the same regimen. 413 children were enrolled and experienced a total of 6027 malaria episodes (mean 15; range, 1–26). For the first malaria episode, the PCR-corrected-cure rate for ASAQ (97.5%) was non-inferior to that for AL (97.0%; 95% CI [−0.028; 0.037]). PCR-corrected cure rates for subsequent malaria episodes that had over 100 cases (episodes 2–18), ranged from 88.1% to 98.9% per episode, with no clear difference between the treatment arms. Parasites were completely cleared by day 3 for all malaria episodes and gametocyte carriage was less than 1% by day 21. Fever clearance was faster in the ASAQ group for the first episode. Treatment compliance for subsequent episodes (only first dose administration observed) was close to 100%. Adverse events though common were similar between treatment arms and mostly related to the disease. Serious adverse events were uncommon, comparable between treatment arms and resolved spontaneously. Anemia and neutropenia occurred in <0.5% of cases per episode, abnormal liver function tests occurred in 0.3% to 1.4% of cases. Both regimens were safe and effective for repeated treatment of malaria.

**Trial Registration:**

Current Controlled Trials NCT00699920

## Introduction

Since 2001, artemisinin-based combination therapy (ACT) is recommended by the World Health Organization (WHO) for the first-line treatment of uncomplicated malaria [1]. The parasite biomass is rapidly reduced by the artemisinin component in the ACT, and the remaining parasites are eliminated by the companion drug [2]. This combination reduces the likelihood of the parasites developing resistance to either drug. Following the ban on monotherapies by the WHO in 2006, the uptake of ACTs has progressively increased in malaria-endemic countries.

Until 2007, the combination of artemether and lumefantrine (AL) was the only fixed-dose ACT widely available in malaria-endemic countries. A fixed-dose combination of artesunate and amodiaquine (ASAQ Winthrop [ASAQ]), developed by a partnership between the Drugs for Neglected Diseases initiative (DNDi) and Sanofi, was prequalified by the WHO in 2008 and included in the List of Essential Medicines in 2011 [3]–[5]. Subsequent studies confirmed a high therapeutic efficacy (94%–98%) of ASAQ [3], , non-inferiority to AL [7], [10], [12] and comparable safety profiles when used for a single episode of uncomplicated malaria [3], [6]–[12]. In most endemic areas, recurrent malaria that requires repeated administration of ACT is inevitable, however the efficacy and particularly the safety of repeated use of ASAQ and other ACT remains to be established.

We conducted a phase IV study to assess the efficacy and safety of repeated use of ASAQ and AL for the treatment of recurrent uncomplicated *P. falciparum* malaria in children less than 5 years old. The study was conducted between 2^nd^ June 2008 and 2nd June 2010 at Nagongera health centre, in Tororo district in Eastern Uganda, an area of intense perennial malaria transmission (annual entomological inoculation rate estimated at 125 infective bites per person per year) [13].

## Methods

### Study design

This was a randomized, open-label, longitudinal, phase IV clinical trial comparing the efficacy and safety of ASAQ and AL for the repeated treatment of uncomplicated malaria during a 2 year study period, in children less than 5 years old. The study was approved by the Faculty of Medicine Makerere University Research and Ethics Committee, the Uganda National Council of Science and Technology, and the Uganda National Drug Authority. The study was carried out in accordance with International Conference on Harmonization Guidelines for Good Clinical Practice and the Declaration of Helsinki. Caregivers of children included in the trial were explained the study objectives and procedures, and written informed consent was obtained from them. The protocol for this trial and supporting CONSORT checklist are available as supporting information; see Checklist S1 and Protocol S1. The study approvals are available as supporting information; see approval S1 and S2.

### Study site

The study was conducted between June 2008 and June 2010 at Nagongera Health Centre, Nagongera sub-county, Tororo district, Uganda. Nagongera is a rural sub-county located in Tororo district in eastern Uganda with a population of 37,715 people in 2009–2010 [14]. Tororo is characterized by high intensity year-round malaria transmission, with an estimated entomological inoculation rate (EIR) estimated at 125 infective bites per person per year) [13]. The majority of malaria cases in the area is caused by *Plasmodium falciparum*, while Anopheles gambiae s.s., and to a lesser extent Anopheles fenestus, are the major vectors [15]. The key malaria control interventions in the district include use of LLINs, malaria case management with ACTs and intermittent preventive treatment during pregnancy (IPTp).

### Study population

Children were included in the study if they were 11) aged 6 to 59 months 2) had uncomplicated malaria with asexual *P. falciparum* mono infection with parasitemia ≥2000/µL 3) had fever (axillary temperature ≥37.5°C) or history of fever within the previous 24 hours, 4) body weight ≥5 kg, and 5) hemoglobin (Hb) concentration ≥5 g/dL. Children were excluded from the study if they had a known allergy to one of the study medications, if they had a history of hepatic or hematological impairment during treatment with amodiaquine, a history of cardiac disease, or had a concomitant febrile illness. Children were excluded if they had severe malaria or danger signs of severe malaria (a recent history of convulsions (1 to 2 within 24 h), unconsciousness, lethargy, inability to drink or breast feed, vomiting, prostration); severe concomitant disease; a known disturbance of electrolyte balance (e.g. hypokalemia or hypomagnesaemia); were receiving a medication metabolized by or that inhibits cytochrome CYP 2D6; were receiving a medication known to prolong the QTc interval; or had received artesunate-amodiaquine or artemether-lumefantrine combination treatment within the previous 2 weeks. Children who experienced a new malaria attack at least 14 days after the previous one were included for subsequent follow up if they had a) a repeat infection with *Plasmodium falciparum, b)* fever (axillary temperature ≥37.5°C) or history of fever within the previous 24 hours, c) body weight ≥5 kg, and d) hemoglobin (Hb) concentration ≥5 g/dL, e) no severe malaria or danger signs. Participants were treated with the same drug and followed up for 42 days.

### Treatments

Following the initial diagnosis of uncomplicated *P. falciparum* malaria, children were referred to a study nurse responsible for the treatment group assignment and allocation of study medications but who was not responsible for patient assessment. A randomization list was computer generated by a statistician in blocks of 4 and provided to the nurse who randomized the children 1∶1 to either the ASAQ or AL treatment group. The randomization codes were provided in an opaque list corresponding to the patient study number. During randomization, the study nurse scraped the opaque case corresponding to the study number in order to determine the allocated treatment. Only the study nurse had access to the sealed treatment randomization list. However, given the variation in appearance, taste and dosing, the other research staff may have known to which arm children were assigned. Supervised treatment allocation and administration of medications was performed by the study nurse who administered all medications orally as follows: ASAQ (ASAQ Winthrop, Sanofi, Paris, France) was administered once daily for 3 days as 1 tablet of 25 mg artesunate/67.5 mg amodiaquine for body weights ≥5 to <9 kg, 1 tablet of 50 mg artesunate/135 mg amodiaquine for weights of ≥9 to <18 kg, or 1 tablet of 100 mg artesunate/270 mg amodiaquine for weights of ≥18 to <36 kg. AL (Coartem, Novartis, Basel, Switzerland) was administered twice daily for 3 days as 1 tablet of 20 mg artemether/120 mg lumefantrine for body weights <15 kg, 2 tablets for weights ≥15 to <25 kg, or 3 tablets for weights of ≥25 to <35 kg. If the child vomited or rejected the medication within the monitoring period, the same dose was re-administered. The administration of all doses for the first episode was directly observed. Patients were kept for 30 minutes after treatment and the dose was re-administered if vomiting occurred. All patients were provided with a 3-day supply of paracetamol for the treatment of febrile symptoms. Patients with hemoglobin <10•0 g/dL were treated with ferrous sulphate for 14 days and given mebendazole if they were over one year of age and had not been treated in the previous 6 months.

Participants included for the second and subsequent follow ups received the same treatment as for the first malaria episode, but treatment was unsupervised after the first dose. If vomiting occurred within 30 min, the child's caregivers were asked to return the child to the study clinic to receive the same treatment. For all episodes, if the child vomited or rejected the medication a second time, a replacement treatment (oral quinine) was administered. All patients who developed severe/complicated malaria during active follow-up were treated with parenteral quinine as per the national guidelines.

### Follow-up Procedures

At enrolment, children's parents or guardians were asked about use of other medications and presence of common symptoms. Axillary temperature and weight were recorded, and a physical examination performed. At follow up visits scheduled for Days 1, 2, 3, 7, 14, 21, 28, and 42, a standardized history was collected and physical examination performed. At each visit, including unscheduled ones, blood samples were collected by venipuncture or finger prick sampling on days 0, 1, 2, 3, 7, 14, 21, 28, and 42 for the first episode of malaria and on days 0, 3, 7, 14, 21, 28, and 42 for subsequent episodes. Aliquots (∼25 µL) were collected onto Whatman Gr3 filter paper, air dried, and stored in sealed sample bags at ambient temperature with a desiccant. The Hb concentration was assessed in blood samples collected on days 0, 3, 7, and 28. If Hb concentration was abnormal (<10 g/dL) on day 7, it was assessed again on day 14. If it was abnormal on day 28, it was assessed again on day 42. Blood hematology and biochemistry was performed on days 0, 7, and 28. Hematology and biochemistry measurements were repeated on day 14 and 42 if results were abnormal on day 7 and day 28 respectively. Parasitemia was assessed in all blood samples by thick and thin blood smears, and malaria parasite genotype was assessed for all patients with outcomes classified as late clinical failure or late parasitological failure. Spleen palpation was performed at each visit for each malaria episode and was scored according to Hackett's classification. Patients' parents were encouraged to attend the clinic at any time if the child was ill. Patients not attending the clinic at the scheduled visits were actively followed up at home. Each patient was followed up actively after recruitment for 42 days (first follow up) and then passively for 2 years. If within the 2-year period the patient presented with a new malaria attack, he/she was enrolled again in the study and treated with the same drugs.

### Laboratory procedures

Thick and thin blood smears were stained with 2% Giemsa for 30 minutes. Parasite density was determined by reading the thick blood smear and counting the number of asexual parasites per 200 white blood cells (WBCs), assuming a WBC count of 8,000/µl. Slides were considered negative if no parasite was detected after reading 100 high-powered fields. Presence of gametocytes was also recorded. Thin blood smears were reviewed for non-falciparum infections. Two microscopists independently read all the slides and parasite densities were calculated by averaging the two counts. Readings with discordant results (difference in species diagnosis, difference in parasite density of >50%, or any difference that affected recruitment or study outcome) were re-examined by a third microscopist; parasite density was calculated by averaging the two closest densities while the final parasite species was determined by two concordant reads. Hemoglobin measurements were made using a portable spectrophotometer Hemocue Hb 201(HemoCue, Ängelholm, Sweden) or using a Humacount 5 hematology counter (Gesellschaft für Biochemica und Diagnostica mbH, Wiesbaden, Germany).

Molecular genotyping was carried out at the Institut de recherche biomédicale des armées-IRBA, Ex-IMTSSA, Marseille, France on samples collected from patients with late treatment failure to discriminate between a recrudescent and a new infection. Parasite deoxyribonucleic acid (DNA) genotype was determined for samples collected on day 0 and on the day of any recurrent parasitemia. For first malaria episodes, parasite DNA was prepared from filter papers by Chelex extraction (Bio-Rad Laboratories, Hercules, CA), and for subsequent episodes, DNA was extracted using a MagMAX DNA Multi-Sample Kit (Life technologies, Gaithersburg, MD). Selected regions of the merozoite surface proteins 1 and 2 (*MSP1* and *MSP2*) and the glutamate rich protein (GLURP) were amplified using nested PCR as described previously [16]. Separate reactions were performed for each pair of primary and nested primers. A no-template control was used in all reactions. Genomic DNA from cloned laboratory strains was used as a positive control for each allele.

PCR products were characterized based on sequence and size polymorphisms identified by gel electrophoresis. Size polymorphisms were analyzed by capillary electrophoresis on polyacrylamide gels using an ABI 3130XL sequencer (Applied Biosystems, Carlsbad, CA) and compared using Genemapper 4.0 software (Applied Biosystems). Genotyping patterns were compared using GelCompar II software (Applied Maths, Sint-Martens-Latem, Belgium).

As described previously [16], children were considered to have recrudescence if at least 1 identical length polymorphism was found in the sample collected at day 0 and on the day of recurrent parasitemia for each marker (*MSP1*, *MSP2*, and *GLURP*). Children were considered to have a new infection if the length polymorphism was different between the sample collected at day 0 and the sample collected on the day of recurrent parasitemia for at least 1 marker. Results were considered indeterminate if DNA amplification was unsuccessful for DNA samples collected on either day 0 or the day of recurrent parasitemia.

### Objectives

The primary objective of the study was to demonstrate the non-inferiority of PCR-adjusted adequate clinical and parasitological response at D28 of ASAQ versus AL during the first malaria episode. The secondary objectives of the study were: 1) to compare the two treatment regiments in terms of D42 efficacy, parasitological and fever clearance, clinical and biological tolerability and evolution of gametocyte carriage during the first malaria episode (observed treatment administration) 2) to compare the two groups of treatment in terms of fever and parasite clearance at D3 and 3) to compare the two groups of treatment in terms of D28 and D42 clinical and parasitological effectiveness, clinical and biological tolerability, fever and parasite clearance at D3, evolution of gametocyte carriage and treatment compliance during the second and following malaria episodes (non observed administration). During the total follow up of the cohort, the study aimed to compare the two groups of treatment in terms of treatment incidence density, clinical and biological tolerability, impact on anemia and Hackett score.

### Outcomes – Efficacy

Treatment outcomes were classified according to the WHO guidelines for areas of intense transmission as adequate clinical and parasitological response (ACPR), early treatment failure (ETF), late clinical failure (LCF), and late parasitological failure (LPF) [17]. Patients were not assigned an efficacy outcome for the following reasons: 1) administration of antimalarial drugs outside the study protocol; 2) withdrawal of consent; and 3) loss to follow-up.

The primary efficacy endpoint was the clinical and parasitological outcome at day 28 adjusted by genotyping for the first malaria episode. Secondary efficacy endpoints included clinical and parasitological outcome at day 28 adjusted by genotyping for subsequent episodes, and clinical and parasitological outcome at day 28 unadjusted by genotyping, clinical and parasitological outcome at day 42, unadjusted and adjusted by genotyping, fever and parasite clearance, gametocytaemia (prevalence and density) at day 7, 14, 21, 28 and 42, hemoglobin changes between day 0 and day 28, and incidence of adverse events for all episodes. The treatment incidence density during the total duration of the study was compared.

### Outcomes - safety

Adverse events (AEs) were defined as any untoward medical occurrence (sign, symptom, disease or abnormal biological value) or clinical investigation in a treated subject, irrespective a causal relationship with the treatment (Guidance for Industry Good Clinical Practice: Consolidated Guidance [ICH E6], April 1996). Treatment-emergent AEs (TEAEs) were defined as AEs that developed or worsened within the study period (42 days) for each episode. All events were graded by the investigator by severity (mild, moderate, severe, life-threatening) using the WHO toxicity grading scale for determining the severity of adverse events and National Institute of Health paediatric toxicity tables, May 2001 guidelines, and the relationship to study treatment was recorded. Physical or clinical symptoms at each scheduled and unscheduled follow-up visit were recorded by the study investigator as an AE if they appeared or worsened during the study. Laboratory and vital sign abnormalities were recorded as AEs only if they were symptomatic, required corrective treatment, led to discontinuation, fulfilled a severity criterion, or were defined as an adverse event of special interest (AESI). AESIs included neutropenia as defined by a neutrophil count <400/mm^3^; blood alanine amino transferase concentration>8× upper limit of the normal range (0–45 IU/L); and blood alanine amino transferase concentration>3× upper limit of the normal range associated with a total bilirubin concentration>2× upper limit of the normal range (0.2–1.0 mg/dL). Serious adverse events (SAEs) were defined as any untoward medical occurrences that resulted in death, were life-threatening, required inpatient hospitalization or prolongation of existing hospitalization, resulted in persistent or significant disability or incapacity, were congenital anomalies or birth defects, or were considered medically important. Hospitalization during the first 3 days of the first malaria episode for social reasons (e.g. parents living too far from the health center to attend the first 4 daily visits) was not considered an AE or SAE.

### Outcomes - compliance

Treatment of the first episode was supervised. For subsequent episodes, for which only the first treatment dose was supervised, blister packages were returned at the end of the third day of treatment by the parent or guardian. Tablets remaining in packages were counted by the study nurse. Treatment compliance was calculated as the percentage of tablets actually taken vs. tablets that should have been taken.

### Insecticide-treated nets (ITNs)

Because of the high number of malaria infections observed during this study, the study stakeholders, in consultation with the ethical review board, decided in July 2009 to provide each child's family with an ITN (PermaNet 3.0, Vestergaard Frandsen, Lausanne, Switzerland). Parents were instructed by a study nurse to place an ITN over the child's bed.

In May 2010, children's families were surveyed about their use of the provided ITNs by a study investigator and, when needed for translation, a community healthcare worker. Briefly, parents or legal guardians were asked about the use of ITNs and the child's sleeping habits. Also, as part of the survey, correct placement of the ITNs in the home over the bed of the child was checked.

### Sample size calculation

The sample size was calculated using NQuery version 4.0 (Statistical Solutions, Saugus, MA) based on the non–inferiority testing of ASAQ versus AL for the first attack (supervised intakes). The acceptable non-inferiority margin (Δ) in proportion of success between ASAQ and AL was chosen at 5%. The test of non inferiority between ASAQ and AL was based on the unilateral confidence interval method with α = 5%, and β = 20%. According to previous studies in East Africa, [8] failure rate at D28 was about 2.7% for AL (approximately 97% of successes). Computing these figures gives us a sample size of 174 patients per treatment group. A total of 200 patients were recruited in each treatment group to account for possible 15% withdrawal or loss to follow-up.

### Statistical analysis

Statistical analysis was performed using SAS version 8.2 (SAS Institute, Cary, NC). All statistical tests were performed at a 5% significance level and were two tailed except for non-inferiority tests. Continuous variables were compared using Student's t-test for normal distributions or a non-parametric Wilcoxon test for distributions that were not normal. Categorical variables were compared using the chi-square test or Fisher's exact test. The intent-to-treat population was defined as all patients who received at least one dose of study medication and who did not have two rejections or vomiting; the per-protocol population comprised all ITT population children who completed the study without major protocol violation and the safety population comprised all patients who received at least one dose of study treatment.

Parasite densities were normalized using logarithmic transformation. Risks of treatment failure were estimated using the Kaplan-Meier product limit formula. Data were censored for patients who did not complete follow-up or were reinfected with non-falciparum species. For the PCR-adjusted ACPR, new *Plasmodium falciparum* infections detected on the basis of genotyping were also censored.

The primary objective, non-inferiority of ASAQ vs. AL in the day-28 PCR-corrected ACPR rate during the first malaria episode, was assessed in the per-protocol and intent-to-treat populations. In addition, in the secondary analyses, PCR-corrected rates for subsequent episodes were compared in the intent-to-treat population. ASAQ was considered non-inferior to AL if the lower limit of the one-sided 95% confidence interval for the difference in rates was greater than −5%. Parasite density, gametocyte carriage, and Hb concentrations were examined in the intent-to-treat population. Safety was assessed in all the patients who received at least one dose of the study treatment.

## Results

### Patient characteristics

**Figure 1:**
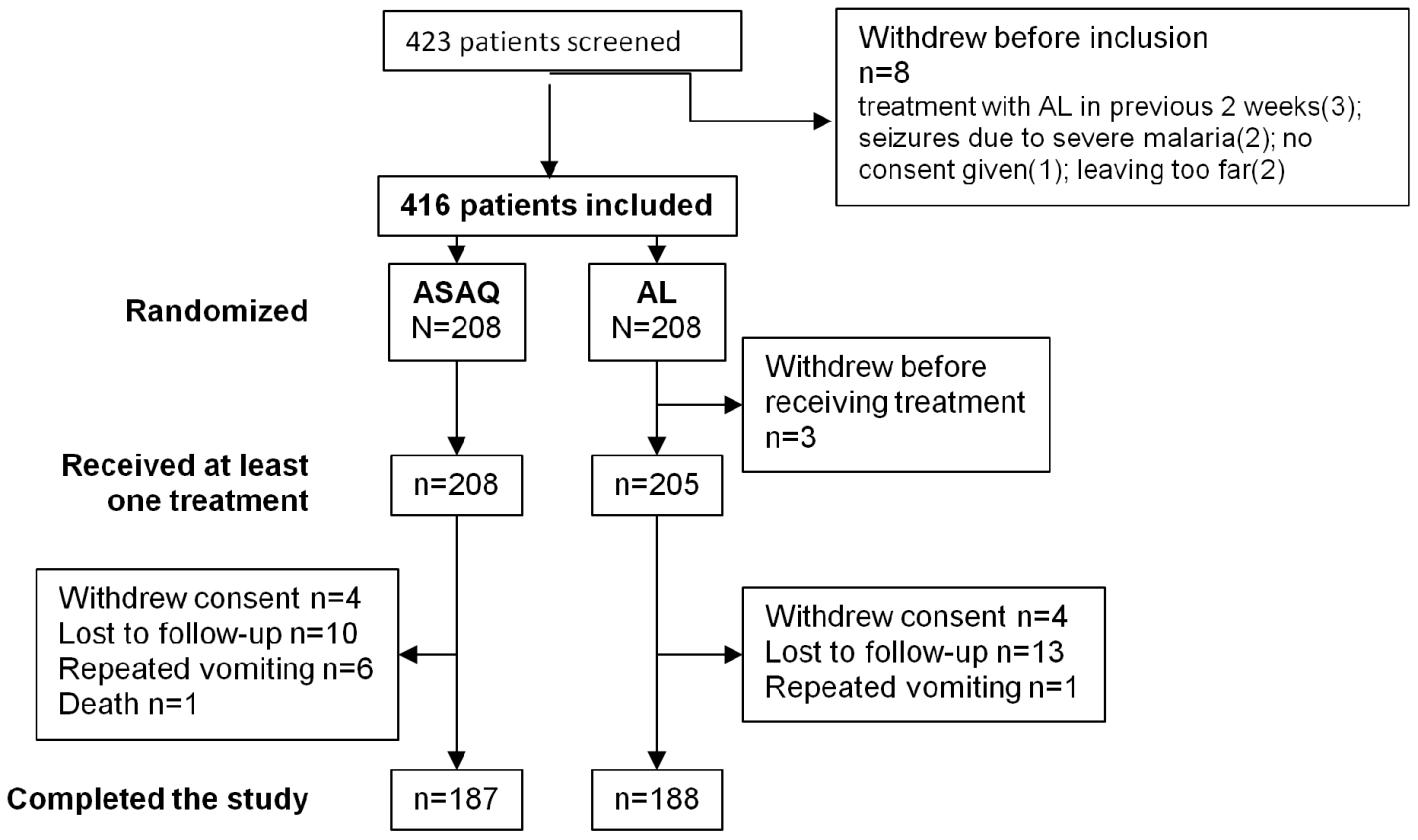
Patient trial profile. A total of 416 children were initially enrolled in the study and were randomized 1∶1 to 3 days of oral ASAQ or AL. Three patients in the AL group were excluded before receiving any treatment. During the 2-year study, another 21 in the ASAQ group and 18 in the AL group withdrew.

Of 422 patients who underwent screening, 416 were randomized to treatment (208 to ASAQ, 208 to AL), and 413 were enrolled in the study (Figure 1). Three children initially randomized to the AL group were not enrolled. A total of 39 treated children withdrew prematurely from the cohort, including 21 in the ASAQ group and 18 in the AL group, while 374 completed the 2 years of study follow up. Baseline characteristics were similar in the two treatment groups (Table 1).

**Table 1:** Baseline characteristics of patients.

	ASAQ	AL
Characteristic	N = 198	N = 201
Sex, n (%)		
Male	96 (48.5)	106 (52.7)
Female	102 (51.5)	95 (47.3)
Age, years		
Mean ± standard deviation	2.21±1.03	2.10±1.01
Range	0.5–4.7	0.5–4.7
Weight (kg)		
Mean ± standard deviation	10.6±2.4	10.3±2.2
Range	6–18	6–18
Height (cm)		
Mean ± standard deviation	82.7±9.8	80.9±8.9
Range	62–106	63–107
Body mass index (kg/m^2^)		
Mean ± standard deviation	15.49±1.76	15.63±1.69
Range	10.7–24.5	12.3–21.6
Splenomegaly (Hackett score)		
0	181 (91.4%)	178 (88.6%)
1	6 (3.0%)	6 (3.0%)
2	11 (5.6%)	17 (8.5%)
Parasite density/µL (geometric mean ± standard deviation)	30,185.9±24,989	29,013.5±26,248
Gametocyte carriage, n (%)	25 (12.6)	26 (12.9)
Gametocytemia (/µL)		
Geometric mean ± standard deviation	96.8±122.1	151.4±210.2
Range	16–440	24–900

Values are for patients from ITT pop.

### Malaria episodes

**Figure 2:**
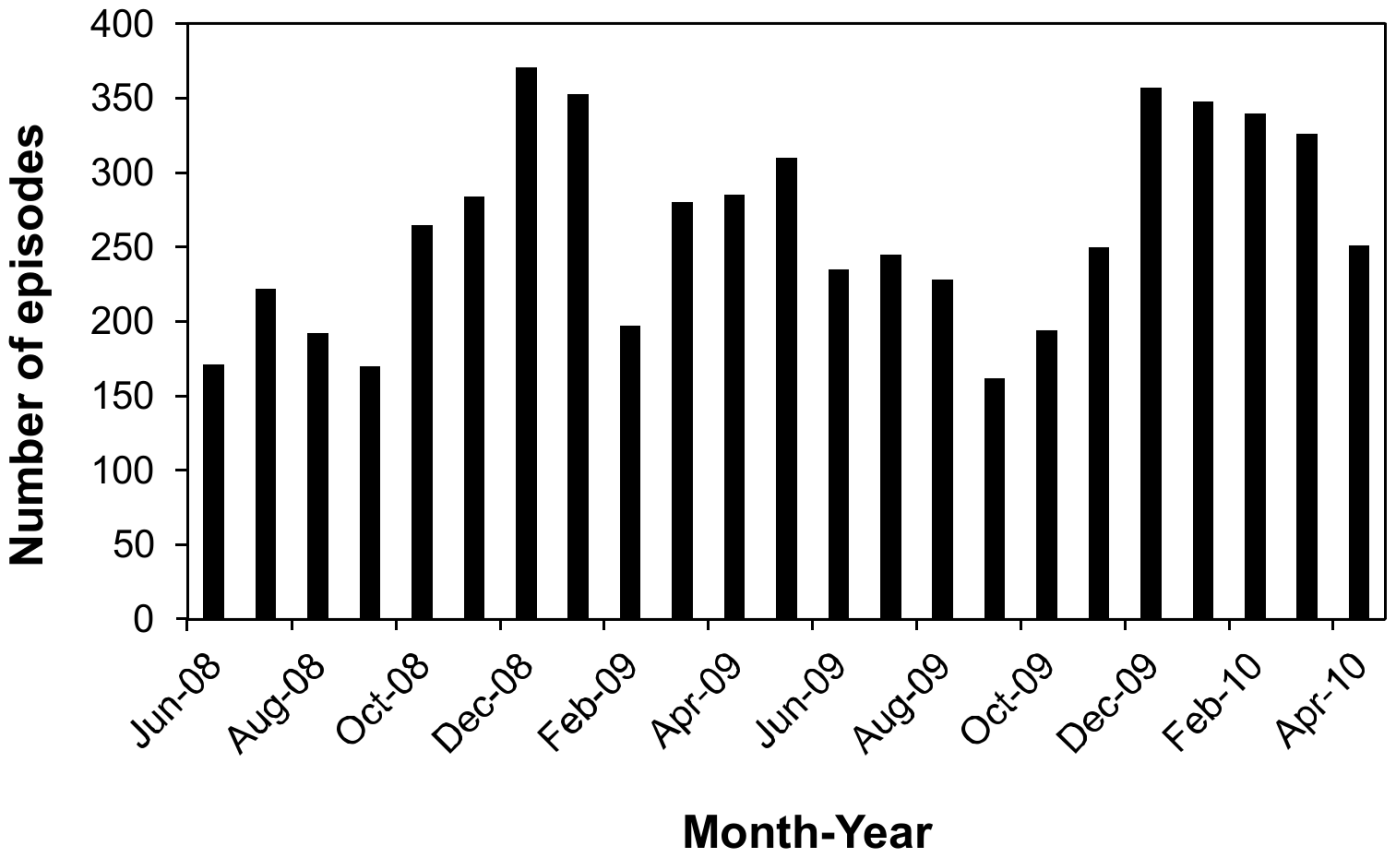
Malaria episodes experienced by patients during the 2 years of the study. (A) Number of children per malaria episode. (B) Number of children with malaria episodes during each month between June, 2008 and April, 2010.

The 413 enrolled children experienced a total of 6027 malaria episodes over the 2 years of the study. The children experienced on average (± standard deviation) 15±5 malaria episodes (range, 1–26) (Figure 2). Children treated with AL experienced slightly fewer episodes of malaria than those who received ASAQ (median, 16 vs. 15; p = 0.011). We also measured the incidence of recurrent malaria, considering all episodes after randomization. The incidence of malaria in study participants randomized to the AL treatment arm was 9.1 treatments per person-year compared to 9.9 treatments per person-year in the ASAQ arm. The mean of the average duration between 2 attacks per patient was longer in the AL than in the ASAQ group (mean ±SD, 49±21 vs. 43±18 days; p = 0.002) while the mean time from D0 of one attack to the D0 of the previous one was significantly greater in the AL group for the 3rd, 4th, 7th, 10th and 19th attack.

A peak in malaria episodes was observed from October 2008 to January 2009, April to May 2009 and from December 2009 to April 2010 (Figure 2). After distribution of ITNs to study participants in July 2009, malaria episodes initially decreased (between September and October 2009), but later rebounded (between December, 2009 and April, 2010) to a level even higher than before the distribution of ITNs.

### Treatment compliance and efficacy

Full treatment compliance was observed for most malaria episodes with the exception of episodes 2, 6, 10, and 20 where one child in the AL group did not take all the study medications. Overall treatment compliance was 100% for ASAQ and greater than 99% for AL.

The PCR-corrected ACPR rate during the first malaria episode was 97.5% for participants treated with ASAQ versus 97.0% for AL in the per-protocol population (95% CI [−0.028; 0.037]) confirming non - inferiority (Table 2). The PCR corrected ACPR rate in the intention to treat population was similarly high (97.5% in ASAQ group versus 97, 0% in AL group; 95%CI [−0.028; 0.037]) (Table 3). Non-inferiority was not met for the uncorrected ACPR rates in both PP and ITT populations.

**Table 2:** Estimates of comparative efficacy of ASAQ and AL for treatment of the first episode of malaria: Per protocol (PP) population.

	ASAQ	AL	Result of statistical comparison
Measure	N = 197	N = 200	
Day 28 PCR-corrected, n (%)			
ACPR	192 (97.5)	194 (97.0)	Non-inferior^a^
LCF	0 (0.0)	3 (1.5)	-
LPF	5 (2.5)	3 (1.5)	-
Day 28 uncorrected, n (%)			
ACPR	103 (52.3)	104 (52.0)	Not non-inferior^a^
LCF	23 (11.7)	35 (17.5)	-
LPF	71 (36.0)	61 (30.5)	-
Proportion without fever, n (%)			
Day 1	195 (98.5)	173 (86.1)	P<0.001^b^
Day 2	194 (98.0)	194 (96.5)	P = 0.543^b^
Day 3	197 (99.5)	194 (96.5)	P = 0.068^b^
Parasite density/µL (geometric mean ± standard deviation)			
Day 0	30,185.9±24989	29,013.5±26248	P = 0.458^c^
Day 1	986.5±2400.8	1214.4±2381.7	P = 0.030^c^
Day 2	1.1±11.5	2.3±14.3	P = 0.099^c^
Day 3	0.0±0.0	0.0±0.0	P = 1.000^c^
Proportion with parasites, n (%)			
Day 1	151 (76.3)	162 (80.6)	P = 0.292^d^
Day 2	2(1%)	7(3.5%)	P = 0.175^b^
Day 3	0	0	-
Time to parasitological clearance (days)			
Mean ± standard deviation	1.8±0.4	1.8±0.5	P = 0.150^c^

Values are for all subjects completing the first episode of the study according to protocol (PP population).

a ASAQ was considered non-inferior to AL if the lower limit of the one-sided 95% confidence interval was>−5%.

b P-value was determined by Fisher's exact test.

c P-value was determined by Wilcoxon's signed-rank test.

d P-value was determined by Chi-squared test.

**Table 3:** Estimates of comparative efficacy of ASAQ and AL for treatment of the first episode of malaria: Intention to treat (ITT) population.

	ASAQ	AL	Result of statistical comparison
Measure	N = 198	N = 201	
Day 28 PCR-corrected, n (%)			
ACPR	192 (97)	194 (96.5)	Non-inferior^a^
LCF	0 (0.0)	3 (1.5)	-
LPF	5 (2.5)	3 (1.5)	-
No outcome	1(0.5)	1(0.5)	
Day 28 uncorrected, n (%)			
ACPR	103 (52)	104 (51.7)	Not non-inferior^a^
LCF	23 (11.6)	35 (17.4)	-
LPF	71 (35.9)	61 (30.3)	-
No outcome	1(0.5)	1(0.5)	
Proportion without fever, n (%)			
Day 1	195 (98.5)	173 (86.1)	P<0.001^b^
Day 2	194 (98.0)	194 (96.5)	P = 0.543^b^
Day 3	197 (99.5)	194 (96.5)	P = 0.068^b^
Parasite density/µL (geometric mean ± standard deviation)			
Day 0	30,185.9±24989	29,013.5±26248	P = 0.458^c^
Day 1	986.5±2400.8	1214.4±2381.7	P = 0.030^c^
Day 2	1.1±11.5	2.3±14.3	P = 0.099^c^
Day 3	0.0±0.0	0.0±0.0	P = 1.000^c^
Proportion with parasites, n (%)			
Day 1	151 (76.3)	162 (80.6)	P = 0.292^d^
Day 2	2(1%)	7(3.5%)	P = 0.175^b^
Day 3	0	0	-
Time to parasitological clearance (days)			
Mean ± standard deviation	1.8±0.4	1.8±0.5	P = 0.150^c^

a ASAQ was considered non-inferior to AL if the lower limit of the one-sided 95% confidence interval was>−5%.

b P-value was determined by Fisher's exact test.

c P-value was determined by Wilcoxon's signed-rank test.

d P-value was determined by Chi-squared test.

**Figure 3:**
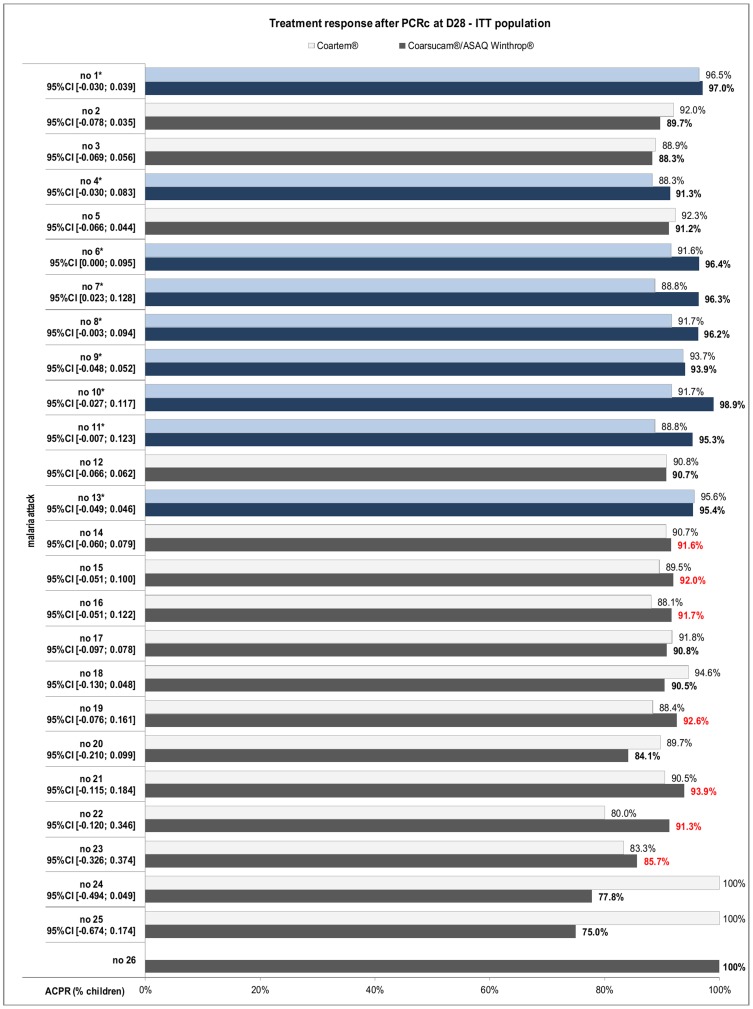
PCR-corrected ACPR rates. Shown is the percent of children in the ASAQ and AL groups with PCR-corrected ACPR on day 28 by month, ITT Population.

The D28 PCR-corrected ACPR rate in subsequent malaria episodes with more than 100 cases (episodes 2 to 18), varied from 88.8% to 98.9% for ASAQ and from 88.1% to 95.6% for AL in the PP population. In the ITT population the D28 PCR-corrected ACPR rate for episodes 2 to 18 varied from 88.3% to 98.9% for ASAQ and from 88.1% to 95.6% for AL in the PP population, (Figure 3 and Table 4 respectively). Non-inferiority of ASAQ vs. AL was demonstrated in 10 of these malaria episodes (episodes1, 4, 6, 7, 8, 9, 10, 11, 13, and 15). PCR-corrected ACPR rates in malaria episodes with less than 100 cases were similar between both treatments, although non-inferiority could not be demonstrated (Table 4). We had similar findings in the intention to treat populations.

**Table 4:** D28 ACPR rates adjusted by genotyping for recurrent malaria episodes with 95% confidence intervals in the PP population.

Malaria episode	Number of cases	D 28 ACPR rates adjusted by genotyping		95% confidence intervals in the PP population
		ASAQ Winthrop	AL	
1	397	97,5%	97,0%	* [−0.028; 0.037]
2	394	89,7%	92,0%	[−0.079; 0.035]
3	392	88,8%	90,3%	[−0.076; 0.045]
4	388	92,3%	89,7%	*[−0.031; 0.083]
5	380	93,6%	93,8%	[−0.050; 0.048]
6	380	96,9%	92,6%	*[−0.002; 0.088]
7	378	96,3%	88,8%	*[0.023; 0.128]
8	364	96,8%	92,2%	*[−0.001; 0.093]
9	354	94,4%	93,7%	*[−0.042; 0.056]
10	344	98,9%	91,7%	*[−0.027; 0.117]
11	330	95,9%	89,4%	*[−0.009; 0.121]
12	310	91,8%	91,4%	[−0.057; 0.066]
13	289	95,4%	95,6%	* [−0.049; 0.046]
14	259	92,3%	91,5%	[−0.059; 0.075]
15	228	93,5%	89,5%	*[−0.033; 0.113]
16	192	91,7%	88,1%	[−0.051; 0.122]
17	159	90,8%	93,1%	[−0.107; 0.062]
18	129	91,8%	94,6%	[−0.115; 0.058]
19	95	92,6%	92,7%	[−0.107; 0.105]
20	72	86,0%	89,7%	[−0.188; 0.116]
21	54	93,9%	90,5%	[−0.115; 0.184]
22	38	91%	80,0%	[−0.120; 0.346]
23	20	85,7%	83,3%	[−0.326; 0.374]
24	13	78%	100%	[−0.494; 0.049]
25	6	75%	100%	[−0.674; 0.174]
26	1	100%		

* =  the efficacy of ASAQ was non inferior to that of AL.

**Figure 4:**
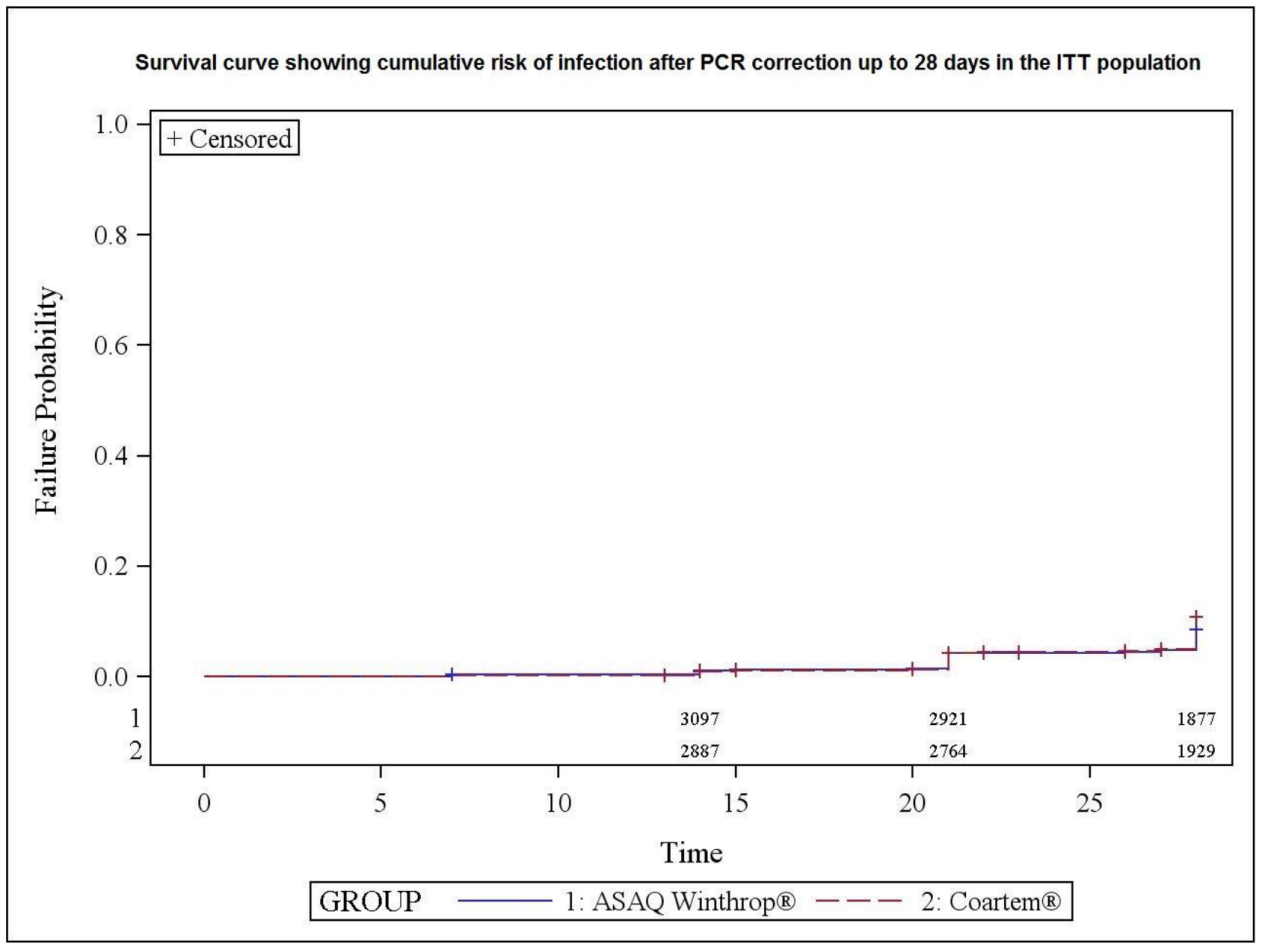
Risk of recurrent parasitaemia. Survival curve showing cumulative risk of infection after PCR correction up to 28 days in the PP population.

Figure 4 shows the survival curve of time to treatment failure after PCR correction for all the malaria episodes in the ITT population. Most episodes of treatment failure were seen>14 days in both treatment arms with a difference in favor of ASAQ (D28 global success rate of 93% with ASAQ versus 91% with AL, p = 0.036).

### Fever clearance

Fever clearance rate was significantly higher in participants treated with ASAQ compared to those treated with AL on day 1 of the first malaria episode (p<0.001), however there was no significant difference on days 2 and 3, with fever clearance rates>96% (Table 2). The proportion of afebrile children (axillary temperature <37.5°C) at day 3 was often ≥98% during the whole study period, and was comparable between the treatment groups.

### Parasite clearance

**Figure 5:**
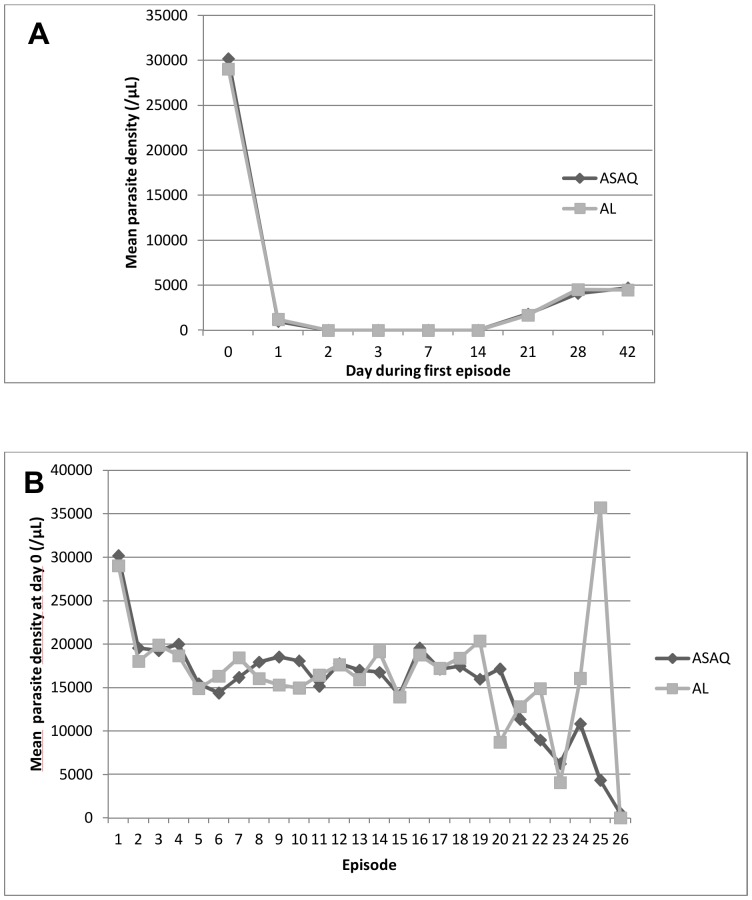
Parasite density. (A) Shows the mean parasite density in the ASAQ and AL groups between days 0 and 42 of the first episode. (B) Shows the mean parasite density in the ASAQ and AL groups at D0 for all episodes.

**Figure 6:**
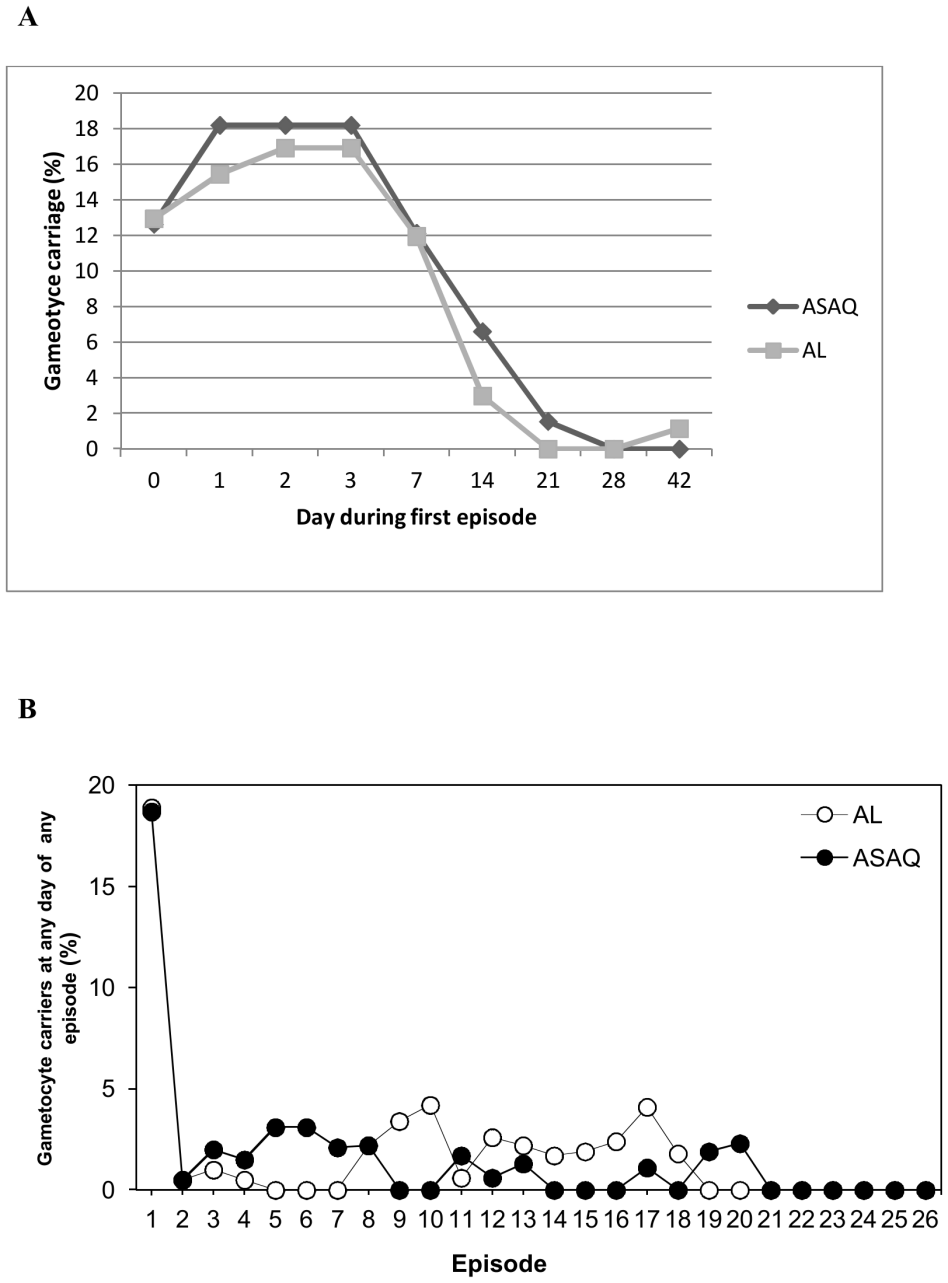
Gametocyte carriage. The proportion of children in the ASAQ and AL groups with gametocytes between days 0 and 42 of the first episode (A) and on any day on the follow up episodes (B).

The mean time to parasite clearance was 1.8 days for both treatment regimens (Table 2). Parasites were completely cleared by day 3 in all children for all episodes (Figure 5A). D0 mean parasite density was over 20000/µl for the first episode, remaining stable (around 10 000/µl) for the follow up episodes (Figure 5B). During the first malaria episode, gametocyte carriage between days 0 to 7 varied from 12.0% to 17.5%, but dropped below 2% by day 21 Figure 6). Gametocyte carriage was comparable between the two treatment groups. In the subsequent malaria episodes, gametocyte carriage between day 0 and 42 was below 5% in both treatment groups (Figure 6B).

### Blood Hb concentration

**Figure 7:**
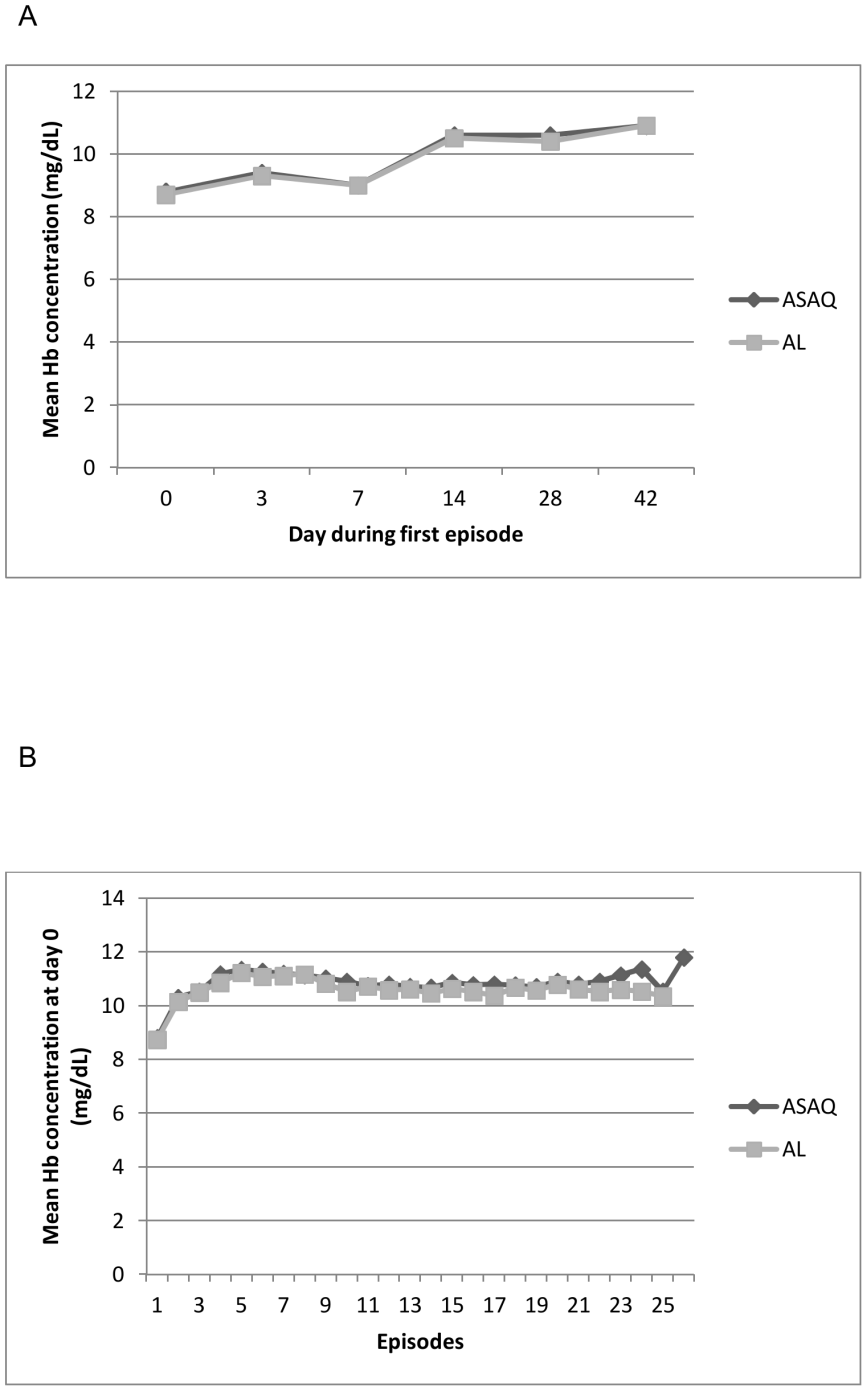
Blood Hb concentrations. Mean blood Hb concentrations in the ASAQ and AL groups between days 0 and 42 of the first episode (A) and on day 0 for all episodes (B). The dashed lined indicates the lower limit of the normal range for blood Hb concentration (10 mg/dL).

During the first malaria episode, mean Hb level was low (<10 g/dl) between days 0 to 7 in both treatment groups however levels became normal by day 14 and continued to increase up to day 42 (Figure 7A). In contrast to the first episode, the median day 0 Hb concentration was within the normal range during the subsequent malaria episodes (Figure 7B).

### Splenomegaly

Splenomegaly was detected on day 0 of the first episode in 8.6% (17/198) of children in the ASAQ group and 11.5% (23/201) of those in the AL group (p = 0.525). All cases were grade 1 or 2. For subsequent episodes, splenomegaly was present in at most 1.2% of the children in each group, and no differences were detected between the ASAQ and AL groups (data not shown).

### Safety

**Figure 8:**
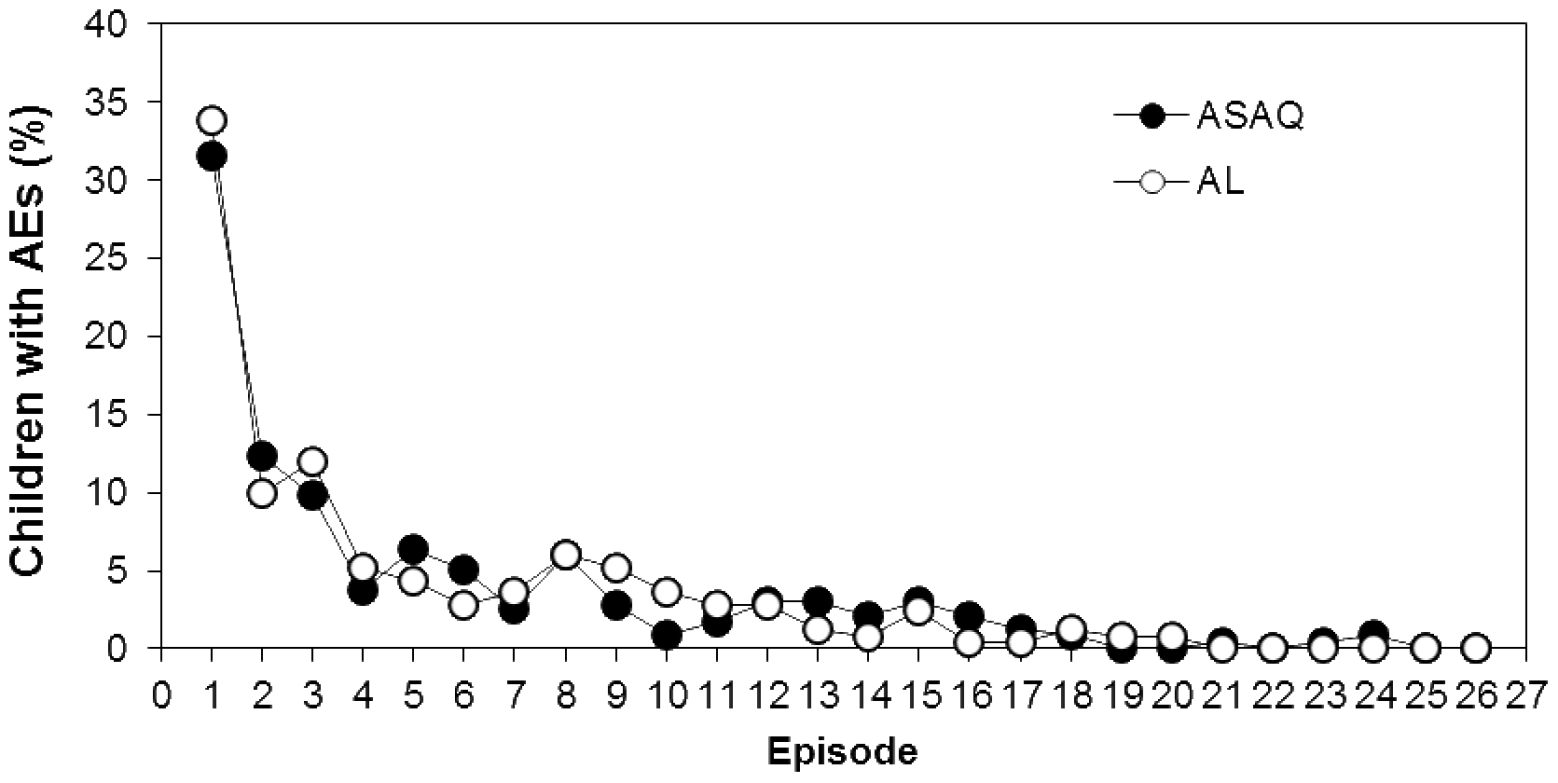
Incidence of AEs by episode. The incidence of AEs experienced per episode by children in the ASAQ and AL groups.

Similar numbers of children in the ASAQ and AL groups reported TEAEs (57.7% vs. 62.0%; p = 0.377) and SAEs (7.7% vs. 4.4%; p = 0.159) as shown in table 5. Most TEAEs were mild or moderate in severity. The most common TEAEs were general disorders (mainly pyrexia; 28.3% of children); infections and infestations (23.0%); eye disorders (mainly conjunctivitis; 15.3%); skin and cutaneous tissue disorders (13.3%); and abnormal laboratory findings (mainly abnormal liver function tests; 6.5%). The two treatment groups did not differ at any visit in the proportion of children with muscle or joint aches, headache, anorexia, nausea, vomiting, abdominal pain or disorders, cough, chest or respiratory rate disorders, viral upper respiratory infection, pruritus, tinnitus, behavioral changes, dehydration, or convulsion (data not shown). Most TEAEs occurred during the first three episodes (Figure 8). Also, no TEAEs considered treatment-related led to study discontinuation.

**Table 5:** Safety outcomes.

	ASAQ	AL	
Event	N = 208	N = 205	P-value^b^
TEAE during any episode, n (%)	120 (57.7%)	127 (62.0%)	0.377
Mild	17 (8.2)	14 (6.8)	
Moderate	106 (51.0)	117 (57.1)	
Severe	28 (13.5)	17 (8.3)	
Treatment-related	1 (0.5)^a^	1 (0.5)^a^	
SAE during any episode	16 (7.7%)	9 (4.4%)	0.159

a Both TEAEs considered treatment-related were severe abnormal liver function tests, which were considered AESIs.

b Determined by Chi-squared test.

AESIs were reported in 14 children in the ASAQ group (6.7%) and 14 (6.8%) in the AL group (table 5). The most common AESIs were abnormal liver function tests (5.6% of children overall) and neutropenia or agranulocytosis (1.5% overall). Abnormal liver function tests mainly included increased serum alanine aminotransferase concentrations, which were most often isolated and rarely associated with increased total bilirubinemia. These AESIs were asymptomatic and, except for two serious abnormal liver function tests (one in the ASAQ group and one in the AL group), were not considered to be treatment-related. All abnormal liver function tests returned to normal without corrective therapy and despite re-administration of treatment within 28 days. Also, all cases of neutropenia and agranulocytosis occurred within 28 days of the last treatment and returned to normal without corrective therapy within 7 to 49 days despite re-administration of treatment, and none were considered treatment-related. Although 15 of these AESIs were SAEs, none led to study treatment discontinuation, required corrective therapy, was life-threatening, or resulted in hospitalization.

SAEs were reported in 16 children in the ASAQ group and in 9 in the AL group (table 5). The most common SAEs were abnormal liver function tests (2.9% overall; also considered AESIs), severe malaria (1.5%), and blood disorders (1.5%). All children recovered from these SAEs except for one 2-year-old girl who died after presenting with severe malaria complicated with severe anemia and congestive heart failure 19 days after the end of ASAQ administration for her 16^th^ malaria episode. Blood tests indicated that she had recrudescence of her infection. These events and her death were not considered by the investigator to be treatment-related.

### ITN use survey

ITNs were distributed to study participants in July 2009 because of the high number of malaria episodes experienced. A survey performed in May 2010 showed that ITNs were distributed to all homes and were correctly placed over the bed of the study participants in 100% of the homes (Table 6). The survey also found that all but two of the children (99.4%) had slept under the ITN the night before, all but four (98.8%) slept under it every night during the 2 weeks before the interview, and all children went to bed by 11 PM.

**Table 6:** Use of ITNs by study participants.

Characteristic	n (%)
**Time participant goes to bed**	N = 355
6–7 pm	7 (2.0)
7–8 pm	45 (12.7)
8–9 pm	191 (53.8)
9–10 pm	106 (29.9)
10–11 pm	6 (1.7)
After 11 pm	0 (0.0)
**Proportion that slept under an ITN the previous night of survey**	N = 353
Slept under ITN	351 (99.4)
**Consistency of ITN use in the last two weeks**	N = 352
Every night	348 (98.9)
Most of the time	3 (0.9)
Rarely	1 (0.3)
**When bed net was obtained.**	N = 355
1 month to <6 months	4 (1.1)
6 months to <1 year	351 (98.9)
**Proportion of bed nets observed hanging^a^**	N = 354
Hanging	354 (100.0)

a Question answered by investigator or an accompanying community healthcare worker.

## Discussion

In malaria-endemic areas, patients suffer from repeated malaria infections and therefore repeatedly use antimalarial drugs. However, most clinical studies have assessed the efficacy and safety of ACTs for only a single malaria episode. This longitudinal phase IV study was conducted in an area of perennial high malaria transmission to compare the safety and efficacy of ASAQ and AL when administered repeatedly over a long period. The study was performed in an area of Eastern Uganda known to have high malaria transmission intensity, with an annual entomological inoculation rate of 125 infectious bites per person per year [13].

The 413 children treated in this study experienced a total of 6027 malaria episodes (range 1 to 26) and on average (± standard deviation) 15±5) episodes over the course of the 2-year study. This is to our best knowledge the highest number of episodes ever reported in studies that have evaluated the repeated use of ASAQ and AL. Patients experienced on average 2 to 3 episodes per year in a 2-year study in Mali [18] and up to 3 episodes in a 16-month study in Senegal [19]. In addition, the longitudinal design of this study allowed efficacy and safety to be assessed continuously over a long period of time. This study therefore provides unique insights into the safety and efficacy of the two most-widely used fixed-dose artemisinin-based combination therapies, ASAQ and AL.

This study showed that ASAQ and AL have high and comparable efficacy profiles for the repeated treatment of malaria in children. The PCR-corrected ACPR for ASAQ was 97.5% and was non-inferior to the rate for AL (97.0%). The PCR-corrected cure rates for ASAQ are comparable to findings reported previously in West and Central Africa (94% to 98%) [3], [6]–[12]. The study also showed that both ACT, cleared parasites within 3 days, Hb concentrations returned to normal within 14 days, and gametocytes were cleared within 21 days. Parasite clearance, normalization of Hb concentrations, and gametocyte clearance did not change over time. Furthermore, both ACT were well tolerated with similar safety profiles despite repeated, long-term use and were highly efficacious for the treatment of recurrent malaria. More importantly there were no patients with any persisting parasitaemia at day 3, indicating that delayed parasite clearance, a phenotype already observed in south East Asia has probably not emerged or reached this Ugandan site.

The number of malaria episodes was slightly, higher for participants treated with ASAQ compared to AL in this study, whereas it was lower or comparable in studies conducted in West Africa [18]–[20]. Furthermore, the time between two different episodes was shorter for ASAQ compared to AL in this study despite amodiaquine having a longer elimination half-life (∼10 days) compared to lumefantrine (∼5 days). Some studies in the same area showed that the post treatment prophylactic effect of drugs with a long elimination half-life was limited in this high transmission setting because of the overwhelming risk of recurrent malaria [21]. However, a recent study observed that compared to AL, the use of dihydroartemisinin-piperaquine (DP) to treat uncomplicated malaria delayed the time to recurrent malaria and reduced the incidences of treatments for malaria probably because of its long half life [22]. Another possible explanation could be resistance to amodiaquine in the study area; further analysis for resistance markers in our study is being undertaken.

The median number of malaria episodes was slightly but significantly higher in the ASAQ group than in the AL group, and the average time between episodes significantly longer in the AL group, with a mean time from D0 of one attack to the D0 of the previous one significantly greater in the AL group for the 3rd, 4th, 7th, 10th and 19th attack. This finding differs from that of a similar study conducted in Mali which showed that patients treated with ASAQ had significantly less risk of recurrent malaria compared to those treated with AL [18]. Our findings also differ from those of a study in Ghana that showed similarity in the incidence of malaria episodes between the two treatments [20]. In addition, pre-existing resistance to amodiaquine may limit its post treatment prophylactic effect in high transmission settings (34). In Uganda, in vitro resistance to AQ has been reported [23] and the prevalence of molecular markers putatively associated with CQ and AQ resistance (pfcrt 76T, pfmdr1 86Y and 1246Y) was very high in 2006 [24]. Analysis of resistance molecular markers will be performed on the samples of this study. PCR-corrected ACPR rates ranged from 88.8% to 98.9% for episodes 2 through 18 and were non-inferior for ASAQ compared to AL in 9 of the first 15 episodes. The lack of statistical non-inferiority for the other episodes is not conclusive because of the small number of children enrolled in these episodes that decreased the power of the analyses. Our study was designed and powered to assess non-inferiority only for the first malaria episode. In the future, such studies should be powered taking into consideration the expected number of repeat episodes in order to enable valid measurement of the differences between regimens with respect to recurrent infections. Nevertheless, for all subsequent episodes, the PCR-corrected ACPR rates were similar between ASAQ and AL.

Following an episode of malaria, even people who are asymptomatic can carry gametocytes and therefore remain reservoirs for infection [25]. Accordingly, eliminating gametocytes is an important goal for malaria treatment and for preventing malaria transmission. A systematic review of six studies in the Gambia and Kenya found that ACTs reduce gametocyte carriage and transmission [26]. Also, a 16-month study in Senegal reported a rapid decrease in malaria episodes in children and adults treated with ASAQ or AL, suggesting that these treatments can reduce gametocyte carriage [19].

The current study showed that both treatments rapidly reduced gametocyte carriage during the first episode and that this reduction in gametocyte carriage persisted during the subsequent episodes. Therefore, repeated treatment with ACTs is not only effective for treating individuals with malaria but might also reduce malaria transmission within the community. In such settings a post treatment dose with an ACT one month after a malaria episode could reduce recurrent episodes and improve the health of the population.

Fever clearance rate was significantly higher in participants treated with ASAQ compared to those treated with AL on day 1 of the first malaria episode (p<0.001), however there was no significant difference on days 2 and 3, where fever clearance rates were>96% on both treatment arms. The exact reason for this is not clear but could be related to the antipyretic properties of amodiaquine, however the supply of antipyretics to patients on the first 3 days, could have led to an over estimation of the fever clearance in both treatment arms.

Repeated administration of ASAQ and AL over a 2-year period in this study did not lead to unexpected safety issues. Safety profiles for both ACTs were good and comparable, and there was no evidence of emerging toxicity due to repeated use. AEs and SAEs were mainly reported during the first malaria episode and were consistent with features of malaria infection or concomitant infection or injuries. Anemia and neutropenia were observed in less than 0.5% of the children per episode, and only 0.3% to 1.4% of the participants had an abnormal liver function test. Increased alanine aminotransferase levels were rarely associated with increased total bilirubinemia. Furthermore all episodes of neutropenia spontaneously returned to normal and did not recur during subsequent treatment administrations.

There was a large reduction in the incidence of the AEs after the first malaria episode. This was probably due to a larger proportion of cases with fever (18.6%) during the first episode compared to less than 5.5% in the subsequent episodes. In the subsequent malaria episodes, children were included on the basis of positive parasitemia without regard for fever or history of fever. Furthermore there could have been more AEs in the first episode due to the closer follow-up and administration of all the treatment doses by the study staff. In this study, repeated treatments did not appear to be associated with hepatic or hematologic toxicity. Similar safety profiles were reported in two other studies that assessed the repeated use of ASAQ and AL, although there were fewer episodes than in the current study [18], [19].

Although one of the studies reported significantly more vomiting with the artesunate-amodiaquine co-blister than with AL (study in Mali) [18], a difference was not observed with the co-formulated ASAQ in the current study or in a previous study in Senegal [19]. In Mali, a co blister of AQ and AS was used with a ratio of AQ/AS of 3.1 instead of the fixed dose combination (FDC) where the corresponding ratio of AQ/AS is 2.7. This ratio was chosen to optimize the dosage in patients treated with the target dose of each of the 2 components [27]. A study in Liberia showed that fixed dose ASAQ and AL were well tolerated in patients of all age groups although certain mild or moderate AEs were more frequent in the ASAQ arm [28]

Administration of treatments was unsupervised for all but the first episode and first intake of subsequent treatments, but full compliance was obtained in all cases except for a few isolated episodes in children treated with AL. Excellent compliance for repeated treatment with ASAQ has been reported previously [19], whereas one study reported poor adherence with AL under non-clinical study conditions [29]. In our study, instructions provided by study nurses to patients' families on the importance of treatment adherence might have limited the difference in compliance between ASAQ and AL.

One year into the study, the unexpectedly high number of malaria episodes raised ethical concerns that led to a decision to distribute ITNs to the children's families. Although study staff members confirmed that the ITNs were properly placed and used, the ITNs did not appear to have a lasting impact on the incidence of malaria episodes. Although study participants had ITNs, coverage of ITN use in the study area was low. The proportion of children <5 who slept under a mosquito net in the area increased from 33.85 in 2006 to 47.8 in 2009 while the proportion of children under 5 who slept under an ITN increased from 12.8% in 2006 to 41.8% in 2010 [30], [31]. The low coverage and use of ITNs in the area could explain the apparent lack of impact of introduction of ITNs on the incidence of malaria. This could have also been due to a rebound in *Anopheles* numbers due to increasing insecticide resistance, as found in a recent longitudinal study on the effect of ITNs in a Senegalese community [32]. This could also have been due to a change in *Anopheles* biting behavior, as reported in 3-year study in Benin [33]. Because ITNs have been reported to reduce malaria morbidity and mortality [34] and are now widely used to combat the parasite [1], the possibility that ITNs are not always effective has generated substantial concern [35]–[37]. Moreover it is possible that even with adequate LLIN coverage, bed nets alone in such a high transmission setting might not offer adequate community protection and combining LLINs with IRS could go a long way in truncating malaria transmission in such settings.

Recent reports of delayed parasite clearance after artemisinin derivative treatment in Southeast Asia have raised concerns of emerging resistance to artemisinin [38]. First-generation ACTs may rapidly increase the frequency of molecular markers associated with resistance to the non-artemisinin partner drug [39], [40]. The absence of parasites at day 3 in all children for all episodes shows that the parasites remained susceptible to the artemisinin derivative component during the full 2 years of the study. This persistent effect of ASAQ is also supported by a 2-year study in Malian adults and children [18].

### Generalizability

This study was conducted in a high transmission area and our findings may be general sable to other settings in Africa with similar malaria transmission intensity.

### Overall evidence

This study is remarkable because of the number of malaria episodes studied, and it clearly showed that ASAQ is safe and effective for repeated use, reduces gametocyte carriage and is as well tolerated and effective as AL.

## Supporting Information

Approval S1 **Makerere Institutional Review Board/Ethics Committee approval document.**
(PDF)

Approval S2 **Uganda National Council of Science and Technology approval document.**
(PDF)

Checklist S1 **Completed CONSORT checklist.**
(DOC)

Protocol S1 **Study protocol.**
(DOC)
