# Supplementary material for: Efficacy and Safety of Fixed-Dose Artesunate-Amodiaquine vs. Artemether-Lumefantrine for Repeated Treatment of Uncomplicated Malaria in Ugandan Children
Source: PLoS One. 2014 Dec 1;9(12):e113311. doi: 10.1371/journal.pone.0113311 (PMC4249977; doi:10.1371/journal.pone.0113311)
Supplement: Protocol S1 — Study protocol. (DOC) [file pone.0113311.s004.doc]

**CLINICAL TRIAL PROTOCOL**

**PRODUCT:**

Coarsucam® (artesunate plus amodiaquine fixed combination)

| **TITLE**  **A RANDOMISED STUDY TO COMPARE A FIXED DOSE COMBINATION OF ARTESUNATE PLUS AMODIAQUINE VERSUS A FIXED DOSE COMBINATION OF ARTEMETHER PLUS LUMEFANTRINE IN TREATMENT OF REPEATED UNCOMPLICATED PLASMODIUM FALCIPARUM MALARIA ATTACKS OCCURRING DURING TWO YEARS OF FOLLOW-UP, IN CHILDREN IN UGANDA** |
| --- |

**STUDY NUMBER: ARAMF_L_02661**

**STUDY NAME: SMART-CURE**

**Final VERSION Date: 18 February 2008**

Sponsor: sanofi-aventis groupe.

« Any and all information presented in this document shall be treated as confidential and shall remain the exclusive property of **sanofi-aventis** (or any of its affiliated companies). The use of such confidential information must be restricted to the recipient for the agreed purpose and must not be disclosed, published or otherwise communicated to any unauthorized persons, for any reasons, in any form whatsoever without the prior written consent of **sanofi-aventis** (or the concerned affiliated company); ‘affiliated company’ means any corporation, partnership or other entity which at the date of communication or afterwards (i) controls directly or indirectly sanofi-aventis, (ii) is directly or indirectly controlled by sanofi-aventis, with ‘control’ meaning direct or indirect ownership or more than 50% of the capital stock or the voting rights in such corporation, partnership or other entity. »

**NAMES AND ADDRESSES OF**

| **COORDINATING INVESTIGATOR** | Name:  Institution  Address  Tel:  Fax:  E-mail: | Dr. Ambrose O. Talisuna MBchB, Msc, PhD  Assistant Commissioner Ministry of Health  Po Box 7272 Kampala ,Uganda  256. 712. 506 275; 256. 414. 345 887  256.414. 540.524  atalisuna@yahoo.com |
| --- | --- | --- |
| **CO-INVESTIGATOR** | Name:  Institution  Address:  Tel:  Fax:  E-mail: | Moses Kamya, MBChB, M Med, MPH  Makerere University Medical School  Department of Medicine, P.O. Box 7072, Kampala, Uganda  256-414-541188  256-414-540524  [malaria@infocom.co.ug](mailto:malaria@infocom.co.ug) |
| **STUDY**  **EPIDEMIOLOGIST** | Name:  Institution  Address:  Tel:  Fax:  E-mail: | Adoke Yeka, MBChB, MPH  Uganda Malaria Surveillance Project  UMSP, P.O. Box 7475, Kampala,  Uganda  256-414-530692; 0772473533  256-414-524540  [yadoke@yahoo.com](mailto:yadoke@yahoo.com); yadoke@muucsf.org |

| **STUDY MANAGEMENT** | Name:  Address:  Tel:  Fax:  E-mail: | Doctor Valérie LAMEYRE  Medical, Access to Medicines, Sanofi-Aventis  74-82 Avenue Raspail  94255 Gentilly Cedex  France  Tel: 00 33 1 41 24 58 56  Mob: 00 33 6 74 44 78 08  Fax: 00 33 1 41 24 58 48  e-mail: valerie.lameyre@sanofi-aventis.com |
| --- | --- | --- |

| **MONITORING TEAM’S REPRESENTATIVE** | Name:  Address:  Tel:  Fax:  E-mail: | Brigitte CHARRON  Medical, Access to Medicines  Sanofi-Aventis  74-82 Avenue Raspail  94255 Gentilly Cedex  France  N° Tél : +33 1 41 24 58 08 /+33 6 80 17 35 99  N° Fax : +33 1 41 24 58 48  E –mail : brigitte.charron@sanofi-aventis.com |
| --- | --- | --- |

| **SPONSOR** | Company:  Address:  Tel: | Sanofi aventis groupe  174 avenue de France,  75013 Paris – FRANCE |
| --- | --- | --- |

| **MONITOR** | Name:  Address:  Tel:  Fax:  E-mail | Tina OLYMPIO  Medical, Access to Medicines  Sanofi-Aventis  74-82 Avenue Raspail  94255 Gentilly Cedex  France  N° Tél : +33 1 41 24 53 13 /+33 6 80 36 44 40  N° Fax : +33 1 41 24 58 48  E –mail : tina.olympio@sanofi-aventis.com |
| --- | --- | --- |

# STUDY TEAM.

| **Name** | **Institution** | **Title** |
| --- | --- | --- |
| Ambrose Talisuna, MBChB, MSc, PhD | Uganda MoH | Principal Investigator |
| Moses Kamya, MBChB, MMED,MPH | MU | Co Investigator |
| Adoke Yeka, MBChB, MPH | UMSP | Epidemiologist. |
| Valérie LAMEYRE, MD | Sanofi Aventis | Medical Manager |
| Brigitte CHARRON | Sanofi Aventis | Project Leader |
| Tina OLYMPIO | Sanofi Aventis | Monitor |
| Samuel Nsobya, MSc | M U | Chief Laboratory Technologist |
| Eruaga Christopher DMLT | UMSP | Laboratory Technologist |
| Buyi Benjamin DMLT | UMSP | Laboratory Technologist. |
| Catherine Tugaineyo | UMSP | Administrator |
| Richard Oluga | UMSP | Accountant |
| Nuhu Kibampawo | UMSP | Driver |
| Kibuka Afizi, MBChB | UMSP | Medical Officer |
| Mudhanga Fred, MBChB | UMSP | Medical Officer |
| Musimenta Grace EN | UMSP | Nurse |
| Adeke Anna EN | UMSP | Nurse |
| Fred Adioma | UMSP | Home Visitor |
| Oburu Moses | UMSP | Home Visitor |

**Table of contents**

[STUDY TEAM. 4](#__RefHeading___Toc191196459)

[1. SYNOPSIS 9](#__RefHeading___Toc191196460)

[2. Flow Chart 13](#__RefHeading___Toc191196461)

[2.1 Study flow chart 13](#__RefHeading___Toc191196462)

[3. CLINICAL TRIAL PROTOCOL AGREEMENT FORM 14](#__RefHeading___Toc191196463)

[4. List of abbreviations 15](#__RefHeading___Toc191196464)

[5. Introduction and rationalE 16](#__RefHeading___Toc191196465)

[5.1 Summary of available information on the treated disease 16](#__RefHeading___Toc191196466)

[5.2 Product 16](#__RefHeading___Toc191196467)

[5.3 Comparator 16](#__RefHeading___Toc191196468)

[5.4 Study rationale 17](#__RefHeading___Toc191196469)

[5.5 Study site 17](#__RefHeading___Toc191196470)

[6. Study objectives 18](#__RefHeading___Toc191196471)

[6.1 Primary 18](#__RefHeading___Toc191196472)

[6.2 Secondary 18](#__RefHeading___Toc191196473)

[7. Study design 18](#__RefHeading___Toc191196474)

[7.1 Description of the Protocol 18](#__RefHeading___Toc191196475)

[7.2 Duration of study 19](#__RefHeading___Toc191196476)

[7.3 Data and Safety Monitoring. 19](#__RefHeading___Toc191196477)

[8. Selection of patients 20](#__RefHeading___Toc191196478)

[8.1 Number of patients planned 20](#__RefHeading___Toc191196479)

[8.2 Inclusion criteria 20](#__RefHeading___Toc191196480)

[8.2.1 Specific inclusion criteria for initial enrollment 20](#__RefHeading___Toc191196481)

[8.2.2 Inclusion criteria for each attack (except the failures) 20](#__RefHeading___Toc191196482)

[8.3 Exclusion criteria 20](#__RefHeading___Toc191196483)

[8.3.1 Specific exclusion criteria for initial enrollment 20](#__RefHeading___Toc191196484)

[8.3.2 Exclusion criteria for each attack 21](#__RefHeading___Toc191196485)

[9. Treatments 21](#__RefHeading___Toc191196486)

[9.1 Investigational Product 21](#__RefHeading___Toc191196487)

[Coarsucam® (artesunate+amodiaquine fixed-dose combination) 21](#__RefHeading___Toc191196488)

[Coartem®  (artemether+lumefantrine): 22](#__RefHeading___Toc191196489)

[9.2 Method of assigning patients to treatment group 22](#__RefHeading___Toc191196490)

[9.3 Packaging and labeling 23](#__RefHeading___Toc191196491)

[9.4 Storage conditions 23](#__RefHeading___Toc191196492)

[9.5 Responsibilities 23](#__RefHeading___Toc191196493)

[9.6 Retrieval and/or destruction of treatments 24](#__RefHeading___Toc191196494)

[9.6.1 Partially used or unused treatment 24](#__RefHeading___Toc191196495)

[9.6.2 Potential recall 24](#__RefHeading___Toc191196496)

[9.7 Concomitant treatment 24](#__RefHeading___Toc191196497)

[9.7.1 Prohibited medication 24](#__RefHeading___Toc191196498)

[9.7.2 Authorized medication 24](#__RefHeading___Toc191196499)

[9.7.3 Replacement treatment 25](#__RefHeading___Toc191196500)

[9.7.4 Management of treatment failures 25](#__RefHeading___Toc191196501)

[9.7.5 Management of non-malaria illnesses 25](#__RefHeading___Toc191196502)

[9.8 Post-Study treatment 26](#__RefHeading___Toc191196503)

[9.9 Treatment accountability and compliance 26](#__RefHeading___Toc191196504)

[10. Assessment of Investigational Product 26](#__RefHeading___Toc191196505)

[10.1 Efficacy 26](#__RefHeading___Toc191196506)

[10.1.1 Primary criteria 26](#__RefHeading___Toc191196507)

[10.1.2 Secondary criteria 27](#__RefHeading___Toc191196508)

[10.1.3 Methods of assessment 27](#__RefHeading___Toc191196509)

[10.2 Safety 28](#__RefHeading___Toc191196510)

[10.2.1 Clinical tolerability: 28](#__RefHeading___Toc191196511)

[10.2.2 Biological tolerability 28](#__RefHeading___Toc191196512)

[10.2.3 Impact of repeated treatment on clinical and biological tolerability 29](#__RefHeading___Toc191196513)

[10.3 Compliance for the 2^nd^ and following attacks 29](#__RefHeading___Toc191196514)

[11. Patient safety 29](#__RefHeading___Toc191196515)

[11.1 Safety instructions 29](#__RefHeading___Toc191196516)

[11.2 Adverse Events monitoring 30](#__RefHeading___Toc191196517)

[11.3 Definitions of Adverse Event (AE) and Serious Adverse Event (SAE) 30](#__RefHeading___Toc191196518)

[11.4 Identification of adverse events 31](#__RefHeading___Toc191196519)

[11.5 Severity, relationship of event to study drug and outcome 31](#__RefHeading___Toc191196520)

[11.6 Reporting of adverse events 32](#__RefHeading___Toc191196521)

[11.7 Obligation of the Investigator regarding safety reporting 32](#__RefHeading___Toc191196522)

[11.8 Adverse Event of special interest (AESI) 34](#__RefHeading___Toc191196523)

[11.9 Obligations of the Sponsor 34](#__RefHeading___Toc191196524)

[12. Handling of patient TEMPORARY OR DEFINITIVE TREATMENT DISCONTINUATION AND OF PATIENT STUDY DISCONTINUATION 34](#__RefHeading___Toc191196525)

[12.1 Treatment discontinuation with investigational product(s) 34](#__RefHeading___Toc191196526)

[12.1.1 List of criteria for treatment discontinuation 34](#__RefHeading___Toc191196527)

[12.1.2 Handling of patients after treatment discontinuation 35](#__RefHeading___Toc191196528)

[12.2 Procedure for withdrawal of patients from the cohort 35](#__RefHeading___Toc191196529)

[12.3 Consequence 36](#__RefHeading___Toc191196530)

[13. Study procedures 36](#__RefHeading___Toc191196531)

[13.1 Management of participants in OPD 36](#__RefHeading___Toc191196532)

[13.2 Visit schedule 36](#__RefHeading___Toc191196533)

[13.2.1 Inclusion visit: D0 36](#__RefHeading___Toc191196534)

[13.2.2 Visits on D1 and D2, and D3 for the 1^st^ attack 37](#__RefHeading___Toc191196535)

[13.2.3 Visit on D3 for the repeat attacks 37](#__RefHeading___Toc191196536)

[13.2.4 Post-treatment follow-up visits on D7 (+/-1), D14 (+/-1), D21 (+/- 1), D28 (+/- 2) and end-of-study visit on D42 (+/- 2) 37](#__RefHeading___Toc191196537)

[13.3 Routine visits 38](#__RefHeading___Toc191196538)

[13.4 After hours visits 38](#__RefHeading___Toc191196539)

[13.5 Medical care outside of the study clinic 38](#__RefHeading___Toc191196540)

[13.6 Definition of source data 39](#__RefHeading___Toc191196541)

[14. Statistical considerations 39](#__RefHeading___Toc191196542)

[14.1 Statistical and analytical plans 39](#__RefHeading___Toc191196543)

[14.2 Determination of sample size 39](#__RefHeading___Toc191196544)

[14.3 Analysis variables 39](#__RefHeading___Toc191196545)

[14.3.1 Demographic and baseline characteristics 39](#__RefHeading___Toc191196546)

[14.3.2 Efficacy variables 40](#__RefHeading___Toc191196547)

[14.3.2.1 Primary efficacy variable 40](#__RefHeading___Toc191196548)

[14.3.2.2 Secondary efficacy variables 40](#__RefHeading___Toc191196549)

[14.3.3 Safety variables 40](#__RefHeading___Toc191196550)

[14.3.3.1 Adverse events 40](#__RefHeading___Toc191196551)

[14.3.3.2 Laboratory safety variables 41](#__RefHeading___Toc191196552)

[14.3.3.3 Vital signs 41](#__RefHeading___Toc191196553)

[14.4 Analysis populations 41](#__RefHeading___Toc191196554)

[14.4.1 Efficacy populations 41](#__RefHeading___Toc191196555)

[14.4.2 Safety population 41](#__RefHeading___Toc191196556)

[14.4.3 Disposition of patients 41](#__RefHeading___Toc191196557)

[14.5 Statistical methods 41](#__RefHeading___Toc191196558)

[14.5.1 Demographics and baseline characteristics 42](#__RefHeading___Toc191196559)

[14.5.2 Extent of study treatment exposure and compliance 42](#__RefHeading___Toc191196560)

[14.5.3 Analysis of efficacy variables 42](#__RefHeading___Toc191196561)

[14.5.3.1 Analysis of primary efficacy variable(s) 42](#__RefHeading___Toc191196562)

[14.5.3.2 Analysis of secondary efficacy variables 43](#__RefHeading___Toc191196563)

[14.5.4 Analysis of safety data 43](#__RefHeading___Toc191196564)

[14.5.4.1 Analysis of adverse events 43](#__RefHeading___Toc191196565)

[14.5.4.2 Laboratory variables analysis 44](#__RefHeading___Toc191196566)

[14.5.4.3 Analysis of vital sign variables 44](#__RefHeading___Toc191196567)

[14.6 Interim analysis 44](#__RefHeading___Toc191196568)

[15. Ethical and regulatory standards 44](#__RefHeading___Toc191196569)

[15.1 Ethical principles 44](#__RefHeading___Toc191196570)

[15.2 Laws and regulations 44](#__RefHeading___Toc191196571)

[15.3 Informed consent 45](#__RefHeading___Toc191196572)

[15.4 Institutional Review Board/Independent Ethics Committee (IRB/IEC) 45](#__RefHeading___Toc191196573)

[16. Study monitoring 46](#__RefHeading___Toc191196574)

[16.1 Responsibilities of the Investigator(s) 46](#__RefHeading___Toc191196575)

[16.2 Responsibilities of the Sponsor 47](#__RefHeading___Toc191196576)

[16.3 Source document requirements 47](#__RefHeading___Toc191196577)

[16.4 Use and completion of Case Report Forms (CRFs) and additional request 47](#__RefHeading___Toc191196578)

[17. Administrative rules 48](#__RefHeading___Toc191196579)

[17.1 Curriculum Vitae 48](#__RefHeading___Toc191196580)

[17.2 Record retention in study site(s) 48](#__RefHeading___Toc191196581)

[18. Confidentiality 48](#__RefHeading___Toc191196582)

[19. Property rights 49](#__RefHeading___Toc191196583)

[20. Data protection 49](#__RefHeading___Toc191196584)

[21. Insurance compensation 49](#__RefHeading___Toc191196585)

[22. Sponsor audits and inspections by regulatory agencies 50](#__RefHeading___Toc191196586)

[23. Premature discontinuation of the study or premature close-out of a site 50](#__RefHeading___Toc191196587)

[23.1 Decided by the Sponsor in the following cases: 50](#__RefHeading___Toc191196588)

[23.2 Decided by the Investigator 50](#__RefHeading___Toc191196589)

[24. Clinical trial results 51](#__RefHeading___Toc191196590)

[25. Publications and communications 51](#__RefHeading___Toc191196591)

[25.1 Publication/communication of study results 51](#__RefHeading___Toc191196592)

[25.2 Public disclosure of clinical trials 52](#__RefHeading___Toc191196593)

[26. Clinical trial protocol amendments 53](#__RefHeading___Toc191196594)

[27. Bibliographic references 54](#__RefHeading___Toc191196595)

[28. Appendices 56](#__RefHeading___Toc191196596)

# SYNOPSIS

| **COMPOUND:** Coarsucam® | **STUDY No.: ARAMF_L_02661** |
| --- | --- |
| TITLE | A randomised study to compare a fixed dose combination of artesunate plus amodiaquine versus a fixed dose combination of artemether plus lumefantrine in treatment of repeated uncomplicated *Plasmodium falciparum* malaria attacks occurring during 2 years of follow-up, in children in Uganda |
| TRIAL LOCATION  Principal investigator | Dr.A Talisuna; Uganda |
| STUDY OBJECTIVE(S) | **Primary:**  To demonstrate the non-inferiority of PCR adjusted adequate clinical and parasitological response at D28 (WHO protocol in vivo D28 2003) of Coarsucam® (artesunate plus amodiaquine) versus Coartem® (artemether plus lumefantrine), based on the first malaria attack of each patient.  **Secondary:**  **For the first attack** (directly observed administration)**:**  To compare the two groups of treatment in terms of:   - D42 efficacy - Parasitological and fever clearance - Clinical and biological tolerability - Evolution of gametocyte carriage   **For repeated attacks**: (non observed administration )  To compare the two groups of treatment in terms of:   - D28 and D42 clinical and parasitological effectiveness - Clinical and biological tolerability - Proportion of patients without fever at D3 - Proportion of patients without parasite at D3 - Evolution of gametocyte carriage - Compliance   **During the total follow up of the cohort**  To compare the two groups of treatment in terms of:   - Treatment incidence density - Impact of repeated treatment on clinical and biological tolerability - Impact on anemia - Evolution of Hackett score |
| STUDY DESIGN | An open randomised, comparative, Phase IV trial |
| STUDY POPULATION |  |
| Specific selection criteria for initial inclusion in the cohort:  Main selection criteria for each attack: | - Children 6 to 59 months - Confirmed mono infection with *Plasmodium falciparum* with parasitemia ≥2000 asexual parasites /µl of blood - No movement from the investigator site area planned throughout the 2-year duration of the trial - No allergy to one of the study drugs - No concomitant febrile illness - Written informed consent of the parents /legal representative - Body weight ≥5 kg - Confirmed *Plasmodium falciparum* infection with positive parasitemia - Able to be treated by oral route - Fever (Axillary temperature ≥ 37,5°C) at D0 or history of fever in the previous 24 hours - Agreement to comply with the study protocol - No danger signs of malaria - No other severe illnesses or underlying diseases - No treatment with artesunate +amodiaquine or artemether-lumefantrine within the previous 2 weeks.   No participation in another ongoing clinical study |
| Total expected number of patients: | 400 patients, 200 per arm |
| Expected number of sites: | 1, Nagongera Health Centre IV, Tororo district |
| INVESTIGATIONAL PRODUCT(S) | **Coarsucam**®, fixed combination of artesunate (AS) and amodiaquine (AQ) |
| Formulation(s): | Infants tablets: AS 25/AQ 67,5 mg  Toddlers tablets: AS 50/AQ 135 mg  Children tablets: AS 100/AQ 270 mg |
| Route(s) of administration: | Oral route  Supervised administration for the first attack  Unsupervised administrations for following attacks (except for the 1^st^ intake) |
| Dose regimen:  COMPARATOR | Once daily, dose according to bodyweight range  Duration of treatment: 3days  **Coartem®** (arthemether+ lumefantrine)  Tablets, 20/120 mg, oral route, twice daily, dose according to bodyweight range.  Duration of treatment: 3 days |
| EVALUATION CRITERIA | - PCR corrected and uncorrected clinical and parasitological cure rate at D28 and D42 ( WHO classification) - Fever and parasitological clearance (for the 1^st^ attack) by measuring the axillary temperature and monitoring parasitemia - Proportion of afebrile patients at D3 and proportion of patients without parasites at D3 (for following attacks) - Clinical efficacy : evolution of baseline symptoms (all clinical symptoms will be graded) - Compliance based on number of residual tablets in blisters - Treatment incidence density during the 2 years of follow up (comparison of the number of malaria attacks between the two arms during the 2 years) - Mean delay between 2 attacks during the 2 years of follow up - Clinical tolerability ( incidence and intensity of recorded AE ) - Biological tolerability (Hb, Bilirubin, ALAT, Creatinine, Leukocytes, Neutrophils, and Platelets count) |
| RESCUE TREATMENT | Because PCR will not be available in real time:  Any failure before D14, will be considered as a recrudescence and will receive rescue treatment  Failure at D14 or later will be considered as reinfection; and the same ACT treatment will be repeated |
| ASSESSMENT SCHEDULE | Refer to flowchart |
| STATISTICAL CONSIDERATIONS | **Number of patients:**  With a non-inferiority margin (Δ) chosen at 5 %, α=5%, and β =20%, and the hypothesis of 97% of success rate with Coartem®, 174 patients are requested; taking account of 15% of lost follow-up 200 patients are needed per treatment group.  **Statistical generalities**  All statistical analyses will be performed on SAS software (version 8.2 for PC).  All statistical tests will be bilateral and performed at a 5% significance level excepted for the non-inferiority analysis (unilateral confidence interval)  **Efficacy Analysis**  The non-inferiority of PCR adjusted adequate clinical and parasitological response at D28 (WHO protocol in vivo D28 2003) of Coarsucam® (artesunate plus amodiaquine) versus Coartem® (artemether plus lumefantrine), based on the first malaria attack of each patient will be studied using the 95% one-sided confidence interval on ITT and Per Protocol populations. |
| DURATION OF STUDY PERIOD | 2 years  Each attack will be actively followed for 42 days |

# Flow Chart

## Study flow chart

| **STUDY PARAMETERS** | **REFERENCE DAYS FOR ALL ATTACKS** | | | | | | | | |  |
| --- | --- | --- | --- | --- | --- | --- | --- | --- | --- | --- |
|  | D0  V1  Inclusion visit | *D1**  *V2* | *D2**  *V3* | D3  V4  End of trt visit | D7  ±1d  V5 | D14  ±1d  V6 | D21  ±1d  V7 | D28  ±2d  V8 | D42 ±2d  V9  End of follow up visit | Un  sche  duled visit |
| Medical history | x |  |  |  |  |  |  |  |  |  |
| Age/Weight/Size/sex | x |  |  |  |  |  |  |  |  |  |
| Physical examination / Vital Signs | x | x | x | x | x | x | x | x | x | x |
| Clinical Tolerability: clinical signs | x | x | x | x | x | x | x | x | x | x |
| Parasitemia  thick and thin smear | x | x | x | x | x | x | x | x | x | x |
| Haemoglobin | x |  |  | x | x | x |  | x | x† | (x) |
| Platelets+ leukocytes + neutrophils | x |  |  |  | x | x^🞊^ |  | x | x† | (x) |
| Creatinemia | x |  |  |  | x | x^🞊^ |  | x | x† | (x) |
| ALT, total bilirubin | x |  |  |  | x | x^🞊^ |  | x | x† | (x) |
| Filter paper for PCR if thick smear + | x |  |  |  | (x) | (x) | (x) | (x) | (x) | (x) |
| Filter paper for DSAQ or L dosage | x# |  |  | x | x |  |  |  |  |  |
| AE record |  | x | x | x | x | x | x | x | x | x |
| Study treatment | x | x | x |  |  |  |  |  |  |  |
| Concomitant treatments | x | x | x | x | x | x | x | x | x | x |

* visits carried out at the 1^st^ attack only

# not performed for the 1^st^ attack

⧫ and in case of severe signs

🞊 if abnormal value at D7

† if abnormal at D28

In case of non-presentation at a planned visit, the patient’s parents shall be contacted again

# CLINICAL TRIAL PROTOCOL AGREEMENT FORM

I, ………………………………………………………………………, the Investigator, have examined the above-referenced sanofi-aventis Clinical Trial Protocol and have fully discussed the objectives of this Clinical Trial and the content of this Clinical Trial Protocol with the Sponsor’s team.

I agree to conduct the Clinical Trial according to this Clinical Trial Protocol and to comply with its requirements, patient to ethical and safety considerations.

In understand that, should the decision be made by the sponsor to terminate prematurely or suspend the Clinical Trial at any time for whatever reason, such decision will be communicated to me in writing. Conversely, should I decide to withdraw from execution of the Clinical Trial, I will communicate such decision in writing to the Sponsor.

INVESTIGATOR

NAME:

DATE:

Signature: ________________________________

FOR THE SPONSOR

NAME:

ADDRESS:

SIGNATORY

NAME:

DATE:

Signature: ________________________________

# List of abbreviations

ACT Artemisinin-based combination therapies

AE Adverse Event

ALT Alanine Aminotransferase

AQ Amodiaquine

AS Artesunate

DSAQ Desethylamodiaquine

DRF Discrepancy Resolution Form

IRB/IEC Institutional Review Board/Independent Ethics Committee

CRF Case Report Form

FOMREC Faculty of Medicine Makerere University Research and Ethics

Committee

NDA the Uganda National Drug Authority,

GCP Good Clinical Practice

ICH International Conference on Harmonisation on of technical requirements for registration of pharmaceuticals for human use

IP Investigational Product

ITT Intent to treat (analysis)

AL Lumefantrine

OPD Out Patient Department

pLDH Plasmodium lactate dehydrogenase

PCR Polymerase chain reaction

NMCP National Malaria Control Program

PP Per protocol (analysis)

RBM Roll Back Malaria

RDT Rapid diagnostic test

SAE Serious Adverse Event

TDR Special program for research and training in tropical diseases (WHO)

UMSP Uganda Malaria Surveillance Project

WHO World Health Organization

# Introduction and rationalE

## Summary of available information on the treated disease

In recent years, the development and propagation of *Plasmodium* *falciparum* strains resistant to conventional antimalarials have considerably hampered the control of malaria in Africa.

In order to limit the spread of chemoresistance and ensure the protection of new antimalarial drugs, national and international experts, in agreement with the WHO, have thus recommended the discontinuation of monotherapy and the use of bitherapy for the treatment of uncomplicated *Plasmodium falciparum* malaria.

This recommendation particularly concerns the use of ACT, which combines conventional antimalarial agents with a prolonged schizonticidal effect, with an oral artemisinin derivative (1).

Several countries have followed these recommendations and modified their policy for the management of uncomplicated malaria attacks by placing ACT at the forefront of the available treatments.

A number of controlled phase III studies under the auspices of the TDR (2), together with other large-scale studies (3) have investigated bitherapy with artesunate combined with amodiaquine, as a single daily dose for 3 days. This well-tolerated combination does not increase the possible undesirable effects arising from amodiaquine alone. This combination greatly improves the cure rates and reduces gametocytaemia, at least transiently, in countries where this antimalarial has become ineffective. A coblister containing artesunate and amodiaquine (Arsucam®) was registered for the treatment of uncomplicated *P. falciparum* malaria attacks in endemic countries since 2002; phase IV studies, conducted in Senegal (4), the Comoros (5) and Mali (6) in over 1000 patients, have confirmed its therapeutic efficacy in the region of 95 to 99%, and its good clinical and laboratory safety profile.

## Product

A fixed-dose combined formulation containing artesunate and amodiaquine in a single tablet has been developed by sanofi-aventis in partnership with DNDI (Drugs for Neglected Disease Initiative), and has been registered under the name of Coarsucam® in endemic countries. The objective of this combined formulation (Coarsucam®) is to improve treatment compliance by eliminating the risk of only one of the two drugs being taken, and by reducing the number of tablets to be administered.

Added to a very similar bioavailability of this coformulation and the separate drugs, two clinical studies confirmed the non inferiority in terms of efficacy and tolerability, versus the loose dose combination of artesunate + amodiaquine (in 750 children under five, Burkina-Faso (7)) and versus Coartem® ( in 941 patients ,Cameroon, Madagascar, Mali, Senegal).

## Comparator

Artemether lumefantrine (AL) is a fixed-combination drug containing the artemisinin derivative, artemether, and lumefantrine (previously known as benflumetol). The fact that AL combines an artemisinin derivative with a novel agent in a fixed-combination regimen is an important advantage of this therapy. The studies conducted with the artemether/lumefantrine fixed-dose combination to date have demonstrated the efficacy and good safety profile of a treatment regimen comprising 6 doses over 3 days in Africa, with therapeutic efficacy rates on D28 in the region of 97 to 98% (8, 9). At present, Coartem® is the WHO reference fixed-dose ACT.

However, AL is administered twice daily (a total of 6 doses over 3 days), and should ideally be taken with fatty food or liquid to maximize absorption, raising concerns about adherence (10).

## Study rationale

This phase IV study will validate the efficacy and safety of Coarsucam® in children under 5 years old in a high transmission area. Effectiveness, compliance and safety of Coarsucam® in repeated administrations for the treatment of consecutive episodes of uncomplicated malaria, in more real conditions of use, (unsupervised intakes) will also be validated. Coarsucam® will be compared to the fixed-dose combination containing an artemisinin derivative currently recommended as first line treatment by Uganda’s health authorities, for this reason*,* patients must be weighting at least 5 kg

## Study site

The study will be conducted in Nagongera Health Centre IV Tororo district, Uganda, an area of very high malaria endemicity. A designated study clinic will be located within the Nagongera Health Centre IV. Patients will be recruited from the catchment area of Nagongera Health Centre IV within a 10 – 15 Km radius. The study clinic will be open daily from 8:00 am to 5:00 pm and after-hours care will be available at Nagongera Health Centre IV. Related clinical and molecular studies will be conducted at affiliated laboratories at the UMSP Molecular Laboratory in Kampala.

Tororo district is situated in Eastern Uganda and borders the republic of Kenya to the east, Mbale district to the northeast, Busia district to the west, Pallisa district to the north and Lake Victoria to the south. It lies along longitude 34 ^o^, 10 ^o^ East and latitude 0^o^,40` North.

It is a low-lying area with rolling hills and savannah grasslands with some dispersed trees. This area experiences a bi-modal rainfall pattern. The first rains peak in March-May and the second rains in August-September. The dry season is longer than the rain season. The average temperature ranges from 18 ^o^ C to 32 ^o^ C and the average humidity is 40-50 %.

Tororo is an area with ethnic diversity. The inhabitants practice mainly subsistence agriculture (11).

The Tororo population experience perennial holoendemic malaria. A survey conducted in 1995 in the Kiyeyi target area in Tororo found spleen rates among sick children aged 2-9 years old of 79% and crude parasite ratios of 47.5% (n=305). *P. falciparum* was responsible for 94.5% of the infections while *P.vivax* accounted for 5.5%. A more recent study in 1999 showed parasite prevalence (*P. falciparum*) of 90.6% (106/117) among asymptomatic children aged 1-9 years (12). Results of an entomological study carried out in 2001 show that the annual entomological inoculation rate for An. gambiae is more than 400 and for An. funestus more than 70 (13).

# Study objectives

## Primary

The objective of this study is to demonstrate the non-inferiority of PCR adjusted adequate clinical and parasitological response at D28 (WHO protocol in vivo D28 2003) of Coarsucam® versus Coartem®, based on the first malaria attack of each patient.

## Secondary

**For the first attack** (observed administration)**:**

To compare the two groups of treatment in terms of:

- D42 efficacy
- Parasitological and fever clearance
- Clinical and biological tolerability
- Evolution of gametocyte carriage

**For attack 2nd and following**: (non observed administration)

To compare the two groups of treatment in terms of:

- D28 and D42 clinical and parasitological effectiveness
- Clinical and biological tolerability
- Proportion of patients without fever at D3
- Proportion of patients without parasites at D3
- Evolution of gametocyte carriage
- Compliance

**During the total follow up of the cohort**

To compare the two groups of treatment in terms of:

- Treatment incidence density
- Impact of repeated treatment on clinical and biological tolerability
- Impact on anaemia
- Impact on Hackett score

# Study design

## Description of the Protocol

This will be a randomized, open, longitudinal Phase IV clinical trial comparing the safety, tolerability, and efficacy of two different combination antimalarial regimens for the treatment of uncomplicated malaria. The clinical study will recruit participants from the catchment area of Nagongera Health Centre IV, and follow clinical care and outcomes for this cohort over a period of 2 years.

## Duration of study

The scheduled duration of the study is 2 years. Each malaria attack that occurs during this period will be followed for 42 days.

Each new malaria attack occurring at least 14 days after the previous one will be considered as a reinfection, will be therefore treated with the same drug and followed up during 42 days.

All patients presenting to the study clinic with a new episode of fever will undergo standard evaluation (history, physical examination and Giemsa-stained blood smear) for the diagnosis of malaria. Participants will be randomized to one of two combination treatment regimens at the time of their first diagnosis of uncomplicated malaria. Each patient will be followed up actively after recruitment for 42 days (first follow up) and then passively for 2 years. If within the 2-year period the patient presents with a new malaria attack, he/she will be enrolled again in the study. At each episode the patient will receive the same drug.

Participants will be followed for 2 years for all routine medical care in the study clinic. All subsequent episodes of uncomplicated malaria will be treated with each participant’s assigned treatment regimen. All clinical treatment failures occurring within 14 days of diagnosis and all episodes of complicated malaria will be treated with quinine, the standard therapy for malaria after treatment failure in Uganda. All episodes diagnosed more than 14 days after a previous episode will be considered new episodes for treatment purposes.

## Data and Safety Monitoring.

A study committee, assembled in conjunction with SANOFI AVENTIS, will be established for the purpose of providing an independent advice on safety of the treatments tested. The committee shall comprise of members who are experts in African health care and/or in malaria disease/ treatment and /or in pharmacovigilance. This committee will review the study protocol prior to implementation of the trial and will be convened to review the study periodically. The committee will review any case report forms containing conflicting or questionable efficacy and/or safety data under blind conditions. One or two meetings may be held during the study as needed. A final meeting will be held in order to finalise the report. In the meantime, telephone conferences may be organised at the request of the sponsor, an investigator or member of the committee. All decisions made by the committee will be documented in writing.

The trial is planned for a period of two years. Interim reports containing information on study progress and data quality (including subject recruitment, patient follow-up, and protocol adherence), and safety data (adverse events, serious adverse events), will be submitted every 6 months to the expert board. This committee will be notified regularly regarding study implementation. Any notifications of serious adverse events will be sent to the committee; the causal relationship with the study treatments will be discussed. The committee will be able to suspend inclusions in all or part of the study groups.

# Selection of patients

## Number of patients planned

Two hundred patients will be enrolled in each group. The duration of the study will be 2 years starting from the enrollment date of the first patient. We anticipate that all the study patients shall be recruited within four months. Once patients have been assigned to a given study drug, each new episode of uncomplicated malaria, occurring after a delay of 14 days, will be retreated with the same treatment previously used.

## Inclusion criteria

### Specific inclusion criteria for initial enrollment

- Male and female children 6 to 59 months
- Confirmed mono infection with *Plasmodium falciparum*, with parasite density ≥ 2000 asexual forms per µl of blood
- Signed (or thumb-printed whenever parents/guardians are illiterate) informed consent by the parents or guardians (informed consent will be asked only at recruitment and will cover the whole period of the study, including subsequent follow up).
- Parents’ or guardians’ willingness and ability to comply with the study protocol for the duration of the trial.

### Inclusion criteria for each attack (except the failures)

- Body weight ≥ 5 kg
- Able to be treated by oral route
- Fever (axillary temperature ≥ 37.5 degrees Celsius) at D0 or history of fever within the previous 24 hours (this criteria is not required in case of late failure after D14 in the previous attack)
- confirmed *Plasmodium falciparum* infection with positive parasitemia
- Haemoglobin value ≥ 5.0 g/dl;
- Agreement to comply with the study protocol

## Exclusion criteria

### Specific exclusion criteria for initial enrollment

- Plan to permanently move out of the investigator site area in the duration of the trial
- patient participating in another ongoing clinical trial
- allergy to one of the investigational medicinal products
- history of hepatic and (or) haematological impairment during treatment with amodiaquine
- history of cardiac disease
- concomitant febrile illness

### Exclusion criteria for each attack

- presence of at least one danger sign of malaria: recent history of convulsions (1-2 within 24h),unconsciousness state, lethargy, unable to drink or breast feed, vomiting everything, unable to stand/sit due to weakness
- severe concomitant disease or known disturbances of electrolyte balance such as hypokalaemia or hypomagnesaemia
- intake of medication metabolised by cytochrome CYP 2D6 (e.g. metoprolol, flecainide, imipramine, amitriptyline, clomipramine) at the time of inclusion
- intake of drugs known to inhibit CYP 2A6 (e.g. methoxsalem, pilocarpine, tranylcypromine) and/or 2C8 cytochromes (e.g. trimethoprim, ritonavir, ketoconazole, montelukast, gemfibrozil) at the time of inclusion
- intake of medication known to prolong the QTc interval, such as class IA and III antiarrythmics, neuroleptics, antidepressant agents, certain antibiotics including drugs in the macrolide class, fluoroquinolones, imidazole and triazole, antifungal agents, certain non-sedative antihistamines (terfenadine, astemizole) and cisapride at the time of inclusion
- patient having received artesunate + amodiaquine or artemether + lumefantrine at

# Treatments

## Investigational Product

### Coarsucam® (artesunate+amodiaquine fixed-dose combination)

Coarsucam® double-layer artesunate/amodiaquine tablets (from Sanofi-aventis); 2 homothetic dosage strengths:

- Infants tablets: AS 25/AQ 67,5 mg
- Toddlers tablets: AS 50/AQ 135 mg
- Children tablets: AS 100/AQ 270 mg

The dosages of Coarsucam® are adapted according to the patient’s body weight:

Body weight ≥5 and <9 kg: daily dosage of 25/67.5mg.

Body weight ≥9 and <18 kg: daily dosage of 50/135 mg*.*

Body weight ≥18 and <36 kg: daily dosage of 100/270 mg*.*

Body weight ≥36 kg: daily dosage of 200/540 mg*.*

Coarsucam® is administered in 1 intake per day.

The treatment duration is 3 days.

The tablets are administered orally with a small amount of still drinking water. For the younger children, the tablets may be dissolved and administered with still drinking water.

### Coartem®  (artemether+lumefantrine):

The artemether/lumefantrine fixed-dose combination tablets (from Novartis) contain 20/120 mg.

The dosages of Coartem® are adapted according to the patient’s body weight:

Body weight <15 kg: daily dosage of 40/240mg in 2 intakes

Body weight ≥15 and <25kg: daily dosage of 80/480mg in 2 intakes

Body weight ≥25 and <35kg: daily dosage of 120/720mg in 2 intakes

Body weight ≥35 daily dosage of 160/960mg in 2 intakes

An interval of at least 8 hours must be maintained between the 1^st^ and the 2^nd^ intake (1^st^ day).

The treatment duration is 3 days.

The tablets are administered orally with still drinking water. Food or milky drink should be taken as soon as possible after each intake.

## Method of assigning patients to treatment group

**9.2.1. Randomisation.**

Prior to the onset of the study, a computer-generated randomization list will be prepared by a statistician. The randomisation code will correspond to the 2 treatment groups. This randomisation list will link each patient’s study number to an opaque zone hiding the treatment group assignment. The list will be accessible only to study nurses. A copy of the original randomization list will be stored by the sponsor in a sealed envelope.

Participants will be randomized to their assigned treatment regimen at the time of their first diagnosis of uncomplicated malaria. Each patient will be identified throughout the study by their patient number allocated at inclusion. This number will consist of a randomization number and the running number of the episode. The randomization number will represent the chronological order of the patient inclusion at the centre. At each randomization number a treatment will be allocated, the name of treatment will be discovered only after recruitment. The treatments will be allocated to the patients according to this list. Each patient will be assigned a single randomization number for the entire duration of the study (2 years).

**9.2.2. Treatment allocation**

Following the diagnosis of their first episode of uncomplicated malaria, participants will be referred to the study nurse who will be responsible for the treatment group assignment and allocation of the study medications. The study nurse will assign treatment groups as follows:

1. On the randomization list select next study number

2. Scrape the opaque case corresponding to study number in order to discover the allocated treatment

3. Record onto the treatment accountability form the study number, enrollment date, treatment assignment name, and study medication dosages to be given.

4. Store form with the randomization list

5. Fill in the treatment allocation page. After the monitoring of the CRF this form will be attached to the CRF

A patient will be treated by the same treatment for all repeated episodes of uncomplicated falciparum malaria, occurring within a minimal delay of 14 days throughout the study (2 years).

Study investigators involved in patient evaluation and treatment outcome classification will be blinded to treatment assignment.. Only nurses responsible for administration of study drugs will remain unblinded to participant’s assigned treatment regimens.

## Packaging and labeling

All test drugs (investigational products- IPs) will be stored in their original packaging in the study clinic. Commercial blister packs of 3 tablets for Coarsucam® and 8 tablets for Coartem® will be used. A label with the study reference, patient number (to be filled in) and the statement “product for use in clinical trials” will be affixed to each blister/pack.

## Storage conditions

The investigational treatments are stored in compliance with the manufacturer’s recommendations (temperature below 30°C and to use before the expired date), in the original packaging and in premises with restricted access.

## Responsibilities

The Investigator or other personnel allowed to store and dispense Investigational Product will be responsible for ensuring that the Investigational Product used in the clinical trial is securely maintained as specified by the Sponsor and in accordance with the applicable regulatory requirements.

All Investigational Product shall be dispensed in accordance with the randomization list and by a study nurse who is not in charge of patient’s assessment. It is the Investigator's responsibility to ensure that an accurate record of Investigational Product issued and returned is maintained. A registry of all study medication will be maintained within the site investigator file (ISF) for the study. The date received, lot number, expiration date, and date used will be recorded for each of the study medications. A record log of investigational will be kept at the study clinic.

Any quality issue noticed with the receipt or use of an Investigational Product (deficient IP in condition, appearance, pertaining documentation, labeling, expiry date, etc.) should be promptly notified to the Sponsor, who will initiate a complaint procedure.

Under no circumstances will the Investigator and his staff supply Investigational Product to a third party, allows the Investigational Product to be used other than as directed by this Clinical Trial Protocol, or dispose of Investigational Product in any other manner.

## Retrieval and/or destruction of treatments

### Partially used or unused treatment

It is the Sponsor’s responsibility to ensure the destruction of all partially used or unused treatments. A detailed treatment form of the returned Investigational Product will be established with the Investigator and countersigned by the Investigator and the Monitoring Team.

Unused test drugs shall be returned to the sponsor for destruction in accordance with the National Drug Authority (NDA) guidelines for re-export of drugs. However, if it is agreeable to the NDA, requests for the compassionate use of the unused test drugs, especially those registered in the country shall be made.

In any case the Investigator will not destroy or dispose of the partially used or unused Investigational Product unless the Sponsor provides written authorization.

### Potential recall

A potential defect in the quality of Investigational Product may be patient to initiation by the Sponsor of a recall procedure. In this case, the Investigator will be responsible for promptly addressing any request made by the Sponsor, in order to recall Investigational Product and eliminate potential hazards.

## Concomitant treatment

### Prohibited medication

Any antimalarials or antibiotic with antimalarial activity (erythromycin or other macrolides, co-trimoxazole or other sulphonamides, any tetracycline including doxycycline, and quinolones, clindamycin…) is not authorised during the study follow up of 42 days. Intake of these drugs leads to withdrawal of the patient from the ongoing active follow up. However, the patient will be kept in the passive follow up and when presenting with another clinical malaria episode fulfilling the inclusion/exclusion criteria, another active follow up can be started. There are no disallowed concomitant therapies during the passive follow up.

### Authorized medication

Fever will be treated with oral paracetamol at a maximum dose of 60 mg/kg/day.

For patients with anaemia (Hb < 10 g/dL), the study team will follow Integrated Management of Childhood Illness (IMCI) guidelines: anemic children will be treated with iron sulfate (100 mg po qD for 2 weeks) and mebendazole (only children > 1 year of age; 250 mg age 1-2 years; 500 mg > 2 years age; treated no more frequently than every 6 months).

Treatments introduced within the 7 days prior to inclusion and/or prescribed during the study shall be recorded in the case report form, including paracetamol, antibiotics or traditional medicines.

### Replacement treatment

Any patient who rejects or vomits the medication within 30 minutes of administration will be retreated with a second dose. Any patient who rejects or vomits repeatedly (> 1 time) will be treated with an oral replacement treatment recommended by Uganda’s authorities. If the patient rejects or vomits this replacement dose he/she will be treated with parenteral quinine and the reason of withdrawal will be “complicated malaria”. If the replacement treatment is accepted the reason of withdrawal will be “repeated vomiting or rejection…”. The reason for treatment discontinuation for the current attack is recorded in the case report form in the section “Reason for treatment discontinuation”. The replacement treatment is to be recorded in the case report form.

### Management of treatment failures

Patients treated for uncomplicated malaria that are classified as failures (within 14 days of treatment) will be treated with quinine (10 mg/kg TID x 7d). In the unlikely event that a patient fails to have an adequate clinical response to quinine (either as first-line therapy for complicated disease or second-line therapy), treatment will be repeated with quinine (3 days) and clindamycin (5 mg/kg QID for 7 days). In this case, a failure CRF will be completed and patient will be followed-up until D42 from the initial enrolment Safety medication will be recorded in the failure case report form.

In experience of the site, failure to respond to quinine therapy is generally an indication of non-compliance. In the very unlikely event that a patient fails to have an adequate clinical response to quinine plus clindamycin therapy or if they develop severe or complicated malaria, they will be referred to Nagongera Health Centre IV or Tororo Hospital for further evaluation.

Treatment failures occurring on D14 or later have to be considering as reinfections and have to be treated with the same ACT allocated by the randomisation.

### Management of non-malaria illnesses

Patients who are found to have illnesses other than malaria will receive standard-of-care treatment in the study clinic, according to standardized algorithms, or will be referred to the appropriate facility at Nagongera Health Centre IV. We will avoid the routine use of non-study medications with antimalarial activity, including tetracycline, antifolate, and macrolide antibiotics, when acceptable alternatives are available. During follow-up for non-malarial illnesses, blood smears will be done at the discretion of the study physician if the patients are febrile (axillary temperature > 37.5˚C) or report history of fever in the past 24 hours. If the blood smear is positive, the patient will be diagnosed with a new episode of malaria and managed per study protocol. If a patient comes to the study clinic for a non-malarial illness and 30 days have passed since the last encounter with the study team, a Routine Visit form will be completed in the study clinic. If a patient is diagnosed with a non-malarial illness at the same time as malaria or during malaria follow-up, treatment will be at the discretion of the physician, but this will have no impact on the management of malaria.

## Post-Study treatment

The study medication dossier is currently under evaluation by Uganda’s health authorities, and will certainly be accessible to the patient before the end of the study.

## Treatment accountability and compliance

For the first attack, each dose of the study treatment will be administered by a study nurse, and the patient will be monitored for 30 minutes after administration. If the patient vomits or rejects the treatment within the 30 minutes, the same dose will be re-administered. In the event of repeated vomiting, a replacement treatment will be given as discussed in 9.7.3. Study participants may choose to either be hospitalised (retained at the clinic) for administration of study medications or to commute from home. Participants will be reimbursed for transport to and from the clinic and for meals or other incidental expenses if they are hospitalised for treatment administration. Amounts for reimbursement will be based on the current rates for transport, meals and incidental expenses. Home visitors shall actively follow patients who do not come for scheduled appointments.

For the repeat attacks the 1^st^ intake will be administered at the study clinic and the other intakes will be unsupervised. At day 3, the patient will give the blister to the study nurse who will check the number of remaining tablets in the returned blister. The parent/guardian will inform the clinical team member of any event related to these intakes. In case of vomiting of an unsupervised intake within the 30 minutes, the patient will visit the study clinic where the same dosage will be administered again.

The study nurse shall record the information on the appropriate CRF page. The Monitor in charge of the study then checks the CRF data by comparing them with the Investigational Product which he/she has retrieved and treatment log forms. All steps will be taken to ensure that patients receive treatment in compliance with the protocol.

# Assessment of Investigational Product

## Efficacy

### Primary criteria

Clinical and parasitological efficacy shall be assessed according to the 2003 WHO in vivo study criteria (according to data of axillary temperature and parasitaemia at D28, after PCR correction.) (cf. Appendix 1). This endpoint is evaluated for the first malaria attack of each patient.

### Secondary criteria

For the first episode:

- Clinical and parasitological efficacy is defined by grading clinical signs further to an interview by the investigator, together with the values obtained for temperature and parasitaemia before and after PCR correction at D42, and before PCR correction at D28
- Monitoring of parasitaemia during the different visits in order to define clearance
- Measure of axillary temperature at each visit in order to define the clearance of fever
- Monitoring of gametocytaemia during each evaluation

For the following episodes:

- Clinical and parasitological effectiveness is defined by grading the clinical signs further to an interview by the investigator, together with the values obtained for temperature and parasitaemia before and after PCR correction at D28 and D42
- Proportion of patients without fever at D3 obtained with the values of temperature (axillary temperature<37.5°C)
- Proportion of patients without parasites at D3 (negative thick smear)

During the total follow up of the cohort:

- Compare the number of malaria attack for each patient between two arms and mean delay between 2 attacks during the 2-years of follow up
- Compare the number of patients with anemia at D0 of each episode during the 2 years per arm.
- Compare the evolution of anaemia by patient between D0 and D28 for each episode

### Methods of assessment

- Physical and Clinical signs and symptoms: the following signs and symptoms will be examined systematically by the investigator and described as being absent, mild, moderate, severe or life threatening: weakness, muscle and/or joint aches, headache, anorexia, nausea, vomiting, abdominal pain, diarrhea, cough, pruritus, tinnitus, behavioural changes, flu, convulsion, dehydration, jaundice, chest ,abdomen, skin (cf. Appendix 2 for grading all of them), Hackett splenic score (score from 0 to 5: Appendix 2d)
- Temperature: axillary temperature will be taken using an electronic thermometer
- Blood pressure (SBP, DBP in mmHg), respiratory rate (pm) and pulse rate (bpm) will be measured after resting for 10 minutes in the sitting position.
- Microscopy

Blood samples will be collected from patients for thick and thin blood smears on days 0, 1, 2, 3, 7, 14, 21, 28, 42 and on any unscheduled day that the patient presents with clinical deterioration or recurrent fever. About a drop of blood is collected for each smear. Thick and thin blood smears obtained from fingerpick blood samples will be stained with Giemsa and read by experienced laboratory technologists who are not directly involved in direct patient care. Parasite and gametocyte densities will be calculated from thick blood smears and thin smears will be used for parasite species identification (Appendix 6). Urgent thick smears will be read in our study clinic for initial diagnosis and to identify treatment failures during follow-up. Routine blood smears will be read within 48 hours. For quality control, all slides will be read by a second microscopist and a third reviewer will settle any discrepant readings.

- Molecular biology methods (PCR): Each time a thick blood smear is obtained blood will also be collected onto filter paper. Samples will be collected by venipuncture or by fingerprick sampling. Blood will be placed onto filter paper in approximately 25 ul aliquots per blood spot. The samples will be labelled, air-dried and stored in small, sealed sample bags at ambient temperature with desiccant. Parasite DNA will subsequently be removed from the filter paper and prepared for molecular analysis using a chelex extraction method. Genotyping will be performed on all patients with outcomes classified as LCF or LPF. Genotyping of parasites collected at baseline (day 0) and the day of recurrent parasitemia will be done to distinguish between true recrudescence and new infections.

Molecular studies will be performed only for research purposes and will have no impact on the clinical management of study patients.

- Anaemia will be assessed by the haemoglobin levels on D0, D3, D7, D14, D28 and D42 if there is still an abnormal value on D28. Haemoglobin measurements shall be done by haemacue or a haematology coulter.

## Safety

### Clinical tolerability:

Incidence and severity of adverse events collected. At each visit, the investigator will ask the patient and parent if he/she has experienced any adverse events since the last visit. Vomiting and diarrhoea will be recorded as adverse event after D3.

For the 2^nd^ and following attacks the patient booklet will be checked by the investigator.

On D0 (except for the 1^st^ attack) blood from a fingerpick will be collected onto filter paper; in case of intolerance to the study drug, levels of desethyl-amodiaquine (DSAQ) or lumefantrine (L) could be assessed to check for the residual quantity of the study drug.

### Biological tolerability

- Haematology and biochemistry will be routinely performed on days 0, 7, and 28. Haematology and biochemistry will be repeated on Day 14 and 42 if results are abnormal on Day 7 and Day 28 respectively. Haematology and biochemistry may be performed on any other day if the clinician feels results are necessary for patient management. An additional haemoglobin evaluation will be performed on day 3 from blood collected by a fingerprick and read using a portable spectrophotometer (Haemacue). Blood samples for haematology and biochemistry will be collected from patients by venipuncture. About 2 mls of blood will be drawn from each patient for biochemistry/haematology during these visits. Additional venipunctures will be performed, as appropriate, for follow-up of adverse events.

Laboratory analyses are described in Appendix 6

- Haematological parameters: haemoglobin, platelets, leukocytes, neutrophils. These parameters will be measured by a complete blood count and assay of haemoglobin and measurements shall be done by a haematology coulter
- Biochemical parameters: blood creatinine, total bilirubin, ALT. These parameters will be measured according to the enzyme assay methods for each parameter.

Note: Any abnormal laboratory (at least grade 3) value will be immediately rechecked, in case of confirmation all investigations to know the reason will be performed and the patient will be followed until normalization. The possible withdrawal of the patient from the cohort will be taken by the investigator.

### Impact of repeated treatment on clinical and biological tolerability

All adverse events will be catalogued based on their frequency, severity, and relationship to study medication using standardized protocols (Appendix 2). These indices of safety and tolerability among treatment groups will be compared using each episode of malaria treated with a study drug as the unit of analysis. The same analyse will be done with the biological parameters.

## Compliance for the 2^nd^ and following attacks

Compliance shall be determined by

- Checking the remaining tablets in returned blisters at D3 by patients. The investigator will count the remaining tablets and in case of discordance the investigator will ask for the reason.
- Dosage of plasma level of desethyl-amodiaquine or lumefantrine. At D3 and D7, a vaccinostyle will be used to collect blood from fingerpick onto filter paper. It will be an exploratory assessment that could be preformed in case of failure or significant intolerance

# Patient safety

## Safety instructions

Safety and tolerability of the treatments will be evaluated by recording Adverse Events (AEs) and grading laboratory and vital signs evaluations. A severity grading scale, based on toxicity grading scales developed by the WHO and the National Institutes of Health, Division of Microbiology and Infectious Diseases, will be used to grade severity of all symptoms, physical exam findings, and haemoglobin results (see Appendix 2 and 3).

All laboratories abnormalities on D28 (ref: Appendix 3)

- Grade 2 or more for biological data
- Clinically significant abnormality (whatever the grade)

have to be followed until resolution or progression has to be stabilized.

For each malaria episode, follow-up for safety will be conducted during the scheduled malaria follow-up visits, and at any unscheduled visit. Follow-up for adverse events reported during a given malaria episode will continue until the patient is re-treated with study medications, or the study ends. If an AE is on going at the time of inclusion for a new malaria episode this AE will be recorded as a concomitant disease at D0 (the AE number recorded at the episode where this AE occurred will be specified to link them). At each episode a new cycle of adverse event assessment and reporting will begin according to the definitions of AE and SAE. Each AE will be considered as one until it has recovered.

## Adverse Events monitoring

All events will be managed and reported in compliance with all applicable regulations, and included in the final clinical study report.

All physical/clinical symptoms described at each visit will be recorded as an adverse event if appears or worsens during the study and is considered to be related to the study drug.

For social reasons a patient may be hospitalized for the first 3 days of the 1^st^ malaria attack (treatment period), this hospitalization will not be reported as an AE/SAE.

## Definitions of Adverse Event (AE) and Serious Adverse Event (SAE)

An **Adverse Event** is any untoward medical occurrence in a patient or clinical investigation patient administered a pharmaceutical product and which does not necessarily have to have a causal relationship with this treatment.

A priori, efficacy endpoints as specified in the protocol will not be considered as AEs except if, because of the course or severity or any other features of such events, the Investigator, according to his/her best medical judgment, considers these events as exceptional in this medical condition.

A **Serious Adverse Event** is any untoward medical occurrence that at any dose:

1. • Results in death or;
2. • Is life-threatening or;

Note: The term "life-threatening" in the definition of "serious" refers to an event in which the patient was at risk of death at the time of the event; it does not refer to an event which hypothetically might have caused death if it were more severe.

1. • Requires inpatient hospitalization or prolongation of existing hospitalization or;
2. • Results in persistent or significant disability/incapacity or;
3. • Is a congenital anomaly/birth defect;
4. • Is a medically important event:

Medical and scientific judgment should be exercised in deciding whether expedited reporting is appropriate in other situations, such as important medical events that may not be immediately life-threatening or result in death or hospitalization but may jeopardize the patient or may require intervention to prevent one of the other outcomes listed in the definition above.

Note: Examples of such events are intensive treatment in an emergency room or at home for allergic bronchospasm, blood dyscrasias, convulsions or a asymptomatic increase of ALT (≥10 ULN) that does not result in hospitalization, or development of drug dependency or drug abuse.

## Identification of adverse events

At each follow-up visit (days 1, 2, 3, 7, 14, 21, 28, and 42, and any unscheduled day), study clinicians will assess patients according to a standardized clinical record form. A severity grading scale, based on toxicity grading scales developed by the WHO and the National Institutes of Health, Division of Microbiology and Infectious Diseases, will be used to grade severity of all symptoms, physical exam findings, and laboratory results as mild, moderate, severe or life threatening (Appendices 2 and 3). Any new event, or an event present at baseline that is increasing in severity, will be considered an adverse event.

## Severity, relationship of event to study drug and outcome

The severity of a clinical adverse event is to be scored according to the following scale:

1. Mild Awareness of sign or symptom, but easily tolerated

2. Moderate Discomfort enough to cause interference with usual activity

3. Severe Incapacitating with inability to work or perform usual activity

4. Life-threatening Patients at risk of death at the time of the event

If the same event worsens in intensity, a new Adverse Event Form should be completed with the date of worsening, a new intensity, etc

The relationship (Yes or No) to the listed Investigational Product will be reported according to the Investigator's judgment

The outcome of the AE will be assessed at the time of the last patient observation according to the following classification:

| Recovered :  Recovered with sequelae | The patient has fully recovered with no observable residual effects  The AE has resulted in a permanent impairment |
| --- | --- |
| Recovering : | Improvement in the patient’s condition has occurred, but the patient still has some residual effects |
| Not recovered : | The AE has not resolved and remains the same as at onset |
| - Death : | The patient died due to the AE |
| - Unknown : | The outcome of the AE is not known because the patient did not return for follow-up (lost to follow-up) |

## Reporting of adverse events

For each possible adverse event identified and graded as mild, moderate, severe or life threatening, an adverse event report form will be completed. The following information will be recorded for all adverse experiences that are reported:

1. Description of event
2. Date of event onset
3. Maximum severity of the event
4. Maximum suspected relationship of the event to study medication
5. Is the event serious?
6. Outcome
7. Date event resolved

## Obligation of the Investigator regarding safety reporting

***Adverse Events***

All Adverse Events regardless of seriousness or relationship to Investigational Product, spanning from the first visit planned in the Clinical Trial Protocol/signature of the informed consent form, up to the last visit planned in the protocol (D42), are to be recorded on the corresponding page(s) included in the Case Report Form.

Whenever possible, symptoms should be grouped as a single syndrome or diagnosis. The Investigator should specify the date of onset, intensity, action taken with respect to Investigational Product, corrective treatment/therapy given, additional investigations performed, outcome and his/her opinion as to whether there is a reasonable possibility that the Adverse Event was caused by the Investigational Product.

Laboratory, vital signs abnormalities are to be recorded as Adverse Events only if they are medically relevant: symptomatic, requiring corrective treatment, leading to discontinuation and/or fulfilling a seriousness criterion and/or is defined as an AESI.

***Serious Adverse Events***

In the case of a Serious Adverse Event the Investigator must immediately:

1. • SEND (within 1 working day, preferably by fax) the signed and dated corresponding page(s) in the Case Report Form **and** the “SAE complementary information” form to

- Dr Valérie Lameyre (address on the cover page)
- Tel.: +33 1 41 24 58 56 or +33 6 74 44 78 08
- or Brigitte Charron: Tel. +33 1 41 24 58 08 or +33 6 80 17 35 99
- ***Fax: +33 1 55 71 95 80***;
- immediately (on the same day) TELEPHONE Valérie Lameyre or the study monitor in the event of death or a life-threatening event;

1. • ATTACH the photocopy of all examinations carried out and the dates on which these examinations were performed. Care should be taken to ensure that the patient's identity is protected and the patient's identifiers in the Clinical Trial are properly mentioned on any copy of source document provided to the Sponsor. For laboratory results, include the laboratory normal ranges;
2. • Follow-up of any Serious Adverse Event that is fatal or life threatening should be provided within one additional calendar week.

- Serious adverse events shall be reported to FOMREC within 7 days while minor adverse events shall be submitted in the annual report.
- Serious adverse events shall be reported to the UNCST and the NDA in prescribed progress reports (annual progress reports).

***Follow-up***

- The Investigator should take all appropriate measures to ensure the safety of the patients, notably he/she should follow up the outcome of any Adverse Events (clinical signs, laboratory values or other, etc.) until the return to normal or consolidation of the patient's condition. If the AE is graded:
- mild (grade 1), the patient will be managed according to good medical practice and the active follow up will be stopped at the last study visit.
- more than 1, the patient will be followed until the AE resolves, improves, or stabilizes. All information collected after the last visit will be recorded in the source documents.
- Any new relevant information concerning the initial SAE is to be described on the “SAE follow-up information form” by the investigator and sent to Dr Valérie Lameyre,
- In case of any Serious Adverse Event, the patient must be followed up until clinical recovery is complete and laboratory results have returned to normal, or until progression has been stabilized. This may imply that follow-up will continue after the patient has left the Clinical Trial and that additional investigations may be requested by the Monitoring Team;
- In case of any Serious Adverse Event brought to the attention of the Investigator at any time after cessation of Investigational Product and considered by him/her to be caused by the Investigational Product with a reasonable possibility, this should be reported to the Monitoring Team.

## Adverse Event of special interest (AESI)

An adverse event of special interest (serious or non-serious) is one of scientific and medical concern specific to the sponsor’s product or program, for which ongoing monitoring and rapid communication by the investigator to the Sponsor may be appropriate. Such events may require further investigation in order to characterize and understand them.

In case of AESI, the Sponsor will be informed immediately (i.e. within 1 working day), even not fulfilling a seriousness criterion, using the AE form together with the SAE complementary form to be sent to the representative of the monitoring team whose name, address and fax number appear on the clinical trial protocol.

All neutropenia with neutrophil count < 400/mm3 and all values of ALAT exceeding 8x upper laboratory normal (ULN) value or ALAT exceeded 3xULN associated to total bilirubin exceeded 2xULN will be considered as AESI. Refer to the specific guideline in Appendix 5 for the management of these AESI.

## Obligations of the Sponsor

During the course of the study, the Sponsor will report in an expedited manner all SAEs that are both unexpected and at least reasonably related to the IP, to the Authorities, IECs / IRBs as appropriate and to the Investigators.

In addition, the Sponsor may report in an expedited manner all SAEs that are expected and at least reasonably related to the IPs to the Authorities, according to local regulations.

Any other AE not listed as an expected event in the Summary of Product Characteristics and in this protocol will be considered as unexpected.

The Sponsor will report all safety observations made during the conduct of the trial in the CSR.

# Handling of patient TEMPORARY OR DEFINITIVE TREATMENT DISCONTINUATION AND OF PATIENT STUDY DISCONTINUATION

The investigational product should be continued whenever possible. Any IP discontinuation should be fully documented in the CRF. In any case, the patient should remain in the study as long as possible.

## Treatment discontinuation with investigational product(s)

### List of criteria for treatment discontinuation

The objective criteria for discontinuing the study treatment and resorting to a safety treatment are as follows:

- Withdrawal of informed consent: The parents/guardians may withdraw the patient from treatment with Investigational Product if they decide to do so, at any time and irrespective of the reason, or this may be the Investigator’s decision
- onset of one or more danger signs/or signs of complicated malaria, according to the WHO definition (cf. Appendix 4),
- parenteral treatment becomes necessary,
- onset of an adverse event justifying treatment discontinuation
- repeated vomiting/rejection

If the patient’s condition progresses in an unsatisfactory manner, he/she will be directed to the centre’s referring facilities.

### Handling of patients after treatment discontinuation

Patients will be followed up according to the study procedures as specified in this protocol up to the scheduled date of study completion (D42), or up to recovery or stabilization of a followed-up AE, whichever comes last.

If possible, and after the discontinuation of treatment, the patients will be assessed using the procedure normally planned for the last dosing day with the Investigational Product (D3) and the final evaluation. After quinine prescription notified, the case report form will be fully completed till D42 and efficacy of quinine will be biologically and clinically evaluated.

## Procedure for withdrawal of patients from the cohort

Enrolled patients will be withdrawn from the cohort for the following reasons:

1. Movement out of the study area for > 60 consecutive days
2. Inability to be located for > 60 consecutive days
3. Withdrawal of informed consent
4. Development of any serious adverse event that requires a withdrawal from study
5. Failure to achieve an adequate clinical response after treatment with quinine plus clindamycin except if it was a severe malaria attack requiring parenteral quinine therapy.
6. Unable to comply with the study schedule and procedures
7. Diagnosis of a serious chronic disease requiring frequent medical care.

If a patient is withdrawn for reasons # 1, 2, or 3, we will be unable to perform any additional study procedures, and will not plan to obtain any follow-up tests. If a patient is withdrawn for reasons # 4, 5 or 7, because a serious health problem developed plans to obtain appropriate follow-up tests will be individualized to each patient. If the patient is withdrawn for reason # 6, plans to obtain appropriate follow-up tests, will be individualized to each patient, depending on the health status of the patient at the time of withdrawal, and the willingness of the participant and their parent/guardian to proceed with additional testing.

- All study withdrawals should be recorded by the Investigator in the appropriate pages when considered as confirmed;
- If possible, the patients are assessed using the procedures normally planned on D28 if withdrawal before D28 or using the procedures planned on D42 if withdrawal between D28 and D42.

The Investigator should make every effort to contact the patient, to identify the reason why he/she failed to attend the visit, and to determine his/her health status, including at least his/her vital status.

Patients who did not complete the study and for whom no endpoint data are available will be considered as lost to follow-up. The statistical analysis plan will specify how these patients lost to follow-up for their primary endpoints will be considered.

## Consequence

Patients who have been withdrawn from a followed-up attack can be maintain or not in the cohort, for potential next attacks, according to investigator’s judgment. Their randomization number of patients not maintained in the cohort must not be reused.

# Study procedures

## Management of participants in OPD

The patients will be screened in out patient department (Appendix 9)

All patients will be enrolled in the cohort during the open hours of the study clinic; the staff should consider that the 2nd intake of Coartem® has to be administered at least 8 hours after the first one.

If a patient has already been recruited for a previous episode, he will be seen by a person informed on the study and treated by the same study drug than previously.

## Visit schedule

### Inclusion visit: D0

The following tests will be carried out:

- Physical examination, collection of medical history, concomitant treatments and demographic data (age, sex), together with body weight and height,
- Vital signs: blood pressure, respiratory and pulse rates at rest, axillary temperature
- Clinical signs and symptoms
- Haematological and biochemical analyses
- Thick and thin smear and filter paper blood spot samples
- Treatment administration
- For 2^nd^ and following attacks:
- Delivery of treatments and patient booklet
- Filter paper blood spot sample for potential DSAQ / L dosage

### Visits on D1 and D2, and D3 for the 1^st^ attack

- Physical examination
- Axillary temperature
- Clinical safety
- Thick and thin smear
- On D1 and D2: Treatment administration
- On D3: filter paper blood spot sample for eventual DSAQ or Lumefantrine dosage.
- On D3: Hg dosage.
- Concomitant treatments
- Adverse events

### Visit on D3 for the repeat attacks

- Physical examination
- Axillary temperature
- Clinical safety
- Hg dosage.
- Thick and thin smear
- Collection and check of returned treatment and patient booklet
- Filter paper blood spot sample for eventual DSAQ or Lumefantrine dosage.
- Concomitant treatments
- Adverse events

### Post-treatment follow-up visits on D7 (+/-1), D14 (+/-1), D21 (+/- 1), D28 (+/- 2) and end-of-study visit on D42 (+/- 2)

- Physical examination
- Axillary temperature
- Clinical safety
- Haematological and biochemical analyses on D7 and D14 (if abnormal results are obtained on D7, except for Hg systematically dosed on D14) on D28 and D42 (if abnormal results are obtained on D28);
- Filter paper blood spot sample for eventual DSAQ or L dosage on D7 only
- Thick and thin smear (and filter paper blood spot samples for eventual PCR)
- Concomitant treatments
- Adverse events

In the event of a positive thick film, a PCR analysis will be carried out on the corresponding blood spot sample.

If a patient cannot perform one visit all efforts will be made to review him/her at the next visit.

## Routine visits

Patients who have not reported to the clinic for any consecutive 30-day period will be visited at home. Patients will be asked about visits to outside health facilities and the use of any medications outside the study protocol. The study protocol will be reinforced with discussion regarding the need to come to the study clinic promptly with the onset of any illness and to avoid use of outside medications. All visits will be recorded in a study registry.

## After hours visits

Study participants will be encouraged to visit Nagongera Health Centre IV outpatients department (OPD), which is adjacent to the study clinic, when urgent care is needed outside of study clinic hours. Participants will be instructed to inform health workers at OPD of their involvement in the study at the time of registration and to visit the study clinic on the following day. An updated list of patients included in the study will be provided to OPD. Identified OPD personnel will be educated about the study protocol and a nurse, employed by the project, will oversee adherence to the study protocol.

If a patient is diagnosed with uncomplicated malaria and has already been assigned a treatment regimen, he/she will receive treatment from an OPD supply of study medications based on a treatment allocation list maintained in the OPD, the data required to fill in the 1st visit of the CRF, should be collected. If a patient is diagnosed with severe malaria, he/she will receive quinine following standard treatment guidelines of both the health facility and this protocol. In these cases the OPD health workers will call one investigator to inform him about the new episode of malaria.

Patients with non-malarial illnesses will be managed at the discretion of the OPD staff. Upon discharge from the OPD, patients will receive follow-up at the study clinic as outlined in the figure. Study personnel will visit the OPD daily to inquire about visits from study patients and facilitate follow-up in the study clinic. The date and reason of all visits will be recorded in a study registry.

## Medical care outside of the study clinic

We will provide all routine medical care, including evaluations, medications available in our clinic, and cost of any transportation free of charge. In addition, we will reimburse the cost of consultation for referrals made by study physicians to other clinics and services within Nagongera Health Centre IV or Tororo Hospital. However, reimbursement of all diagnostic tests and treatment recommended outside the study clinic cannot be guaranteed in all circumstances. Decisions on reimbursement will be made by the study coordinator and the investigators, in conjunction with the sponsor if necessary.

## Definition of source data

Concomitant diseases together with any concomitant medication will be recorded directly in the case report form (CRF) after the interview with the parent.

Clinical signs and the results obtained for vital signs may be recorded directly in the case report form after the tests.

The visit dates, demographic data will be recorded in the health centre registry or in the patient’s source medical file, together with the history of fever (if applicable), the thick film results, any onset of adverse events and any new treatments taken. The printouts for the laboratory test results will be filed with the CRF, if the printouts are on thermal paper a copy of the results will be done and the document will be validated/ signed by the person responsible of laboratory tests.

Treatment accounts will be recorded directly on the sheet provided for that purpose.

A copy of the results of PCR determinations will be filed.

# Statistical considerations

## Statistical and analytical plans

The material of [Section 14](#_Ref99339812) of the Clinical Trial Protocol is the basis for the Statistical Analysis Plan for the study. This plan may be revised during the study to accommodate Clinical Trial Protocol amendments and to make changes to adapt to unexpected issues in study execution and data that affect planned analysis. These revisions will be based on blinded review of the study and data, and a final plan will be issued before database lock.

## Determination of sample size

The main analysis will be based on the non–inferiority testing of Coarsucam® (fixed dose combination of artesunate plus amodiaquine) versus Coartem® (artemether plus lumefantrine) for the first attack (supervised intakes).

The acceptable non-inferiority margin (Δ) in proportion of success between Coarsucam® and Coartem®, has been chosen at 5 %.

The test of non inferiority between Coarsucam® group and Coartem® group will be based on the unilateral confidence interval method with α=5%, and β =20%,

According to Mutabingwa (7) failure rate at D28 is about 2.7% for Coartem ® (97% of successes) in East Africa (Tanzania).

With these hypotheses (computed with Nquery advisor Software, process PTE1a-1) 174 patients are needed by group of treatment.

Finally 200 patients will be included in each group in order to take account of 15% of prematurely withdrawal.

## Analysis variables

### Demographic and baseline characteristics

The demographic data and parameters recorded on inclusion will undergo a descriptive analysis according to treatment groups on the safety population.

The evaluations used as baseline values will be the last evaluations performed before treatment administration. Any repeated evaluations will be considered baseline values if performed prior to the first intake of treatment.

### Efficacy variables

#### Primary efficacy variable

The main criterion is defined as the adequate response to the treatment at D28, after PCR correction according to WHO guidelines for assessing response to treatment (2003), for the first attack and for both treatment groups.

#### Secondary efficacy variables

Comparison of both treatment groups in terms of:

- Response to treatment at D28, before PCR correction for the first attack
- Response to treatment at D28, before and after PCR correction for all repeated attacks
- Response to treatment at D42, before and after PCR for each attack
- Time to parasite clearance (proportion of aparasitaemic patients at 24, 48 and 72 hours) for the first attack
- Time to fever clearance (proportion of afebrile patients at 24, 48 and 72 hours) for the first attack
- Proportion of afebrile patients at D3 for the following attacks
- Proportion of patients without parasite at D3 for the following attacks
- Evolution of number of gametocytes carriers during the 42 days of follow-up and for each episode
- Evolution of the mean of gametocytes during the 42 days of follow-up and for each episode
- Evolution of abnormal biological baseline value for each attack
- Number of malaria attacks during the 2 years of follow up (treatment density)
- Evolution of the average duration between 2 attacks during the 2 years of follow up
- Evolution of haemoglobin value for each patient during each attack (between D0 and D28) during the 2 years of follow up
- Evolution of mean haemoglobin values at D0,D3, D7,D14 and D28 for each episode during the 2 years of follow up
- Evolution of Hackett score during the 2 years of follow-up

### Safety variables

#### Adverse events

- Adverse events recorded during the follow up
- Serious adverse events
- Deaths

#### Laboratory safety variables

Haematological parameters: haemoglobin, platelets, leukocytes, neutrophils

Biochemical parameters:, blood creatinine, total bilirubin, ALT

#### Vital signs

- Axillary temperature at each time of measurement

## Analysis populations

### Efficacy populations

The following populations will be defined for the analysis,:

- Intent to treat (ITT) population: all included patients known to have received at least one dose of treatment (excluding patients with double rejection or vomiting at the first intake).
- Per protocol (PP) population all ITT population patients that completed the study treatment in accordance with the study protocol and completed all specified assessments without major violation

### Safety population

Safety population: all randomized patients who have got at least one dose of treatment, including patients with double vomiting or rejection at the first intake.

Number of patients in each population will be displayed by treatment group.

### Disposition of patients

The status of protocol deviations (minor/major) will be listed and discussed during a blind review meeting with the study committee.

## Statistical methods

All statistical analyses will be performed on SAS software (version 8.2 for PC).

All statistical tests will be performed at a 5% significance level and will be bilateral except for the non-inferiority analysis (unilateral confidence interval).

Distributions of parameters will be summarized by mean, standard deviation, median, minimum and maximum. These distributions will be compared using the Student t-test if normal distribution hypothesis is confirmed. Otherwise, distributions will be compared using the non-parametric Wilcoxon test.

All qualitative parameters will be summarized in contingency tables displaying frequencies and corresponding percentages.

For these parameters, a chi-square test (replaced by Fisher exact test if the expected frequency in any one of the cells of the contingency table is < 5) will be performed.

### Demographics and baseline characteristics

Comparability at baseline (of the 1^st^ episode) between the two treatment groups will be assessed on the safety, ITT and PP populations for demographics and baseline characteristics.

The medical history / concomitant diseases (coded using system organ MedDRA) will be compared by group of treatment at the randomization time and described for the following attacks.

Concomitants treatments (coded using the WHO Drug Dictionary (ATC Code)) will be compared by group of treatment and described for the following attacks.

### Extent of study treatment exposure and compliance

The analysis of the compliance will be performed on the safety population and on the ITT population.

Number of tablets taken

Compliance = ---------------------------------------------------------------------------- x 100

theoretical Total number of tablets taken

The compliance will be calculated for each attack.

The evolution of the compliance will be analyzed for each patient during the 2 years of follow-up.

The mean compliance will be presented by group of treatment and overall.

An exploratory analysis could be performed by dosing blood level of DSAQ or Lumefantrine on D3.

### Analysis of efficacy variables

The efficacy data analysis will be performed on the ITT population and on the Per Protocol population.

#### Analysis of primary efficacy variable(s)

The main criterion is defined as the response to the treatment after PCR correction according to WHO guidelines 2003 for the first attack.

The response will be classified such as:

- ETF (Early Treatment Failure)
- LPF ( Late Parasitological Failure)
- LCF (Late Clinical Failure)
- ACPR (Adequate Clinical and Parasitological Response)

The proportion of successes will be compared by group of treatment.

The main analysis is strictly defined as the non-inferiority analysis for the first attack.

The test of non inferiority between Coarsucam® group and Coartem® group will be based on the confidence interval method: the lower limit of the 95% one-sided confidence interval for the proportion difference between the two treatment groups should be greater than -Δ to conclude non-inferiority.

#### Analysis of secondary efficacy variables

Non inferiority analysis will be performed on the same way than for the main criterion for

- before PCR corrected cure rate at D28 for the first attack
- before and after PCR corrected cure rate at D28 for the following attacks
- before and after PCR corrected cure rate at D42 for all attacks

An analysis for repeated measurements will be performed. A model will be built in order to explain the success/failure of the response. The rank of the attack, the group of treatment and their interaction will be included in the model. Some other relevant parameters could be included if descriptive analysis find out discriminating items.

Comparison between treatment groups will be provided for the following parameters

- Time to parasitological clearance for the 1^st^ episode (number of patients without parasite from D1 to D3)
- Time to fever clearance for the 1^st^ episode (number of patients without temperature from D1 to D3)
- Proportion of patients without fever at D3 for repeated attacks
- Proportion of patients without parasite at D3 for repeated attacks
- Evolution of number of gametocyte carriers and mean values of gametocyte rate during the 42 days of follow-up for each episode
- Evolution of hemoglobin value for each patient during each attack (between D0 and D28) occurring during the 2 years of follow up
- Evolution of mean hemoglobin values at D0,D3, D7,D14 and D28 of each episode during the 2 years of follow up
- Evolution of Hackett score during the 2 years of follow up

The number of malaria attacks will be summarized by treatment group and overall.

The evolution of average duration between 2 attacks during the 2 years of follow up will be computed and analyzed using survival analysis methods.

### Analysis of safety data

This analysis will be performed on the safety population.

#### Analysis of adverse events

Treatment-emergent AEs (TEAEs) are defined as AEs that developed or worsened during the study period (time from first dose of study medication up to D42 days after the first dose of study medication) for each attack.

All adverse events will be coded using MedDRA. The following frequency distributions of adverse events (incidence tables) will be provided for the safety population:

- All and possibly related TEAEs by system organ class,
- Possibly related TEAEs by system organ class and by decreasing frequency,
- All and possibly related TEAEs by intensity and system organ class,
- Serious adverse events: All and possibly related TEAEs by seriousness criterion,
- Serious adverse events: All and possibly related TEAEs by system organ class,
- Death: All and possibly related TEAEs by system organ class,
- Other significant adverse events: All and possibly related TEAEs by “other significant” adverse event criterion,
- Discontinuation: All and possibly related TEAEs by system organ class,
- Laboratory abnormalities: All and possibly related TEAEs,

#### Laboratory variables analysis

Biological parameters will be summarized by group of treatment and overall at each time of measurement.

All abnormal variables will be graded and each parameter will be counted by grade.

The number of patients with abnormal data will be compared by grade between each treatment group and over the 2 years of follow-up

Transition tables will be provided between D0, D7 and D28 in order to assess the evolution of these parameters classified as normal/abnormal and by grade according to the laboratory ranges.

Absolute and relative changes to the baseline will be computed for each parameter for each attack. The evolution between D0 and D7 and D0 and D28 will be analyzed for each attack

#### Analysis of vital sign variables

Not applicable

## Interim analysis

No interim analysis is planned for this study.

# Ethical and regulatory standards

## Ethical principles

This Clinical Trial will be conducted in accordance with the principles laid down by the 18th World Medical Assembly (Helsinki, 1964) and all applicable amendments laid down by the World Medical Assemblies and the ICH guidelines for Good Clinical Practice (appendix 8).

## Laws and regulations

This Clinical Trial will be conducted in compliance with all international laws and regulations, and national laws and regulations of Uganda, as well as any applicable guidelines.

## Informed consent

The Investigator (according to applicable regulatory requirements), or a person designated by the Investigator, should fully inform the patient and the parent[s] or guardian[s] of all pertinent aspects of the clinical trial including the written information given approval / favorable opinion by the Faculty of Medicine Makerere University Research and Ethics Committee (FOMREC), Uganda National Council of Science and Technology UNCST, the Uganda National Drug Authority, NDA. All participants should be informed to the fullest extent possible about the study in language and terms they are able to understand.

Prior to a patient’s participation in the clinical trial, the Informed Consent Form should be signed and personally dated by the patient’s parent(s) or by the patient’s legally acceptable representative, and by the person who conducted the informed consent discussion. Whenever possible, the consent will be signed by both parents/guardians. If only one parent or guardian signs the consent form, the investigator must document the reason for only one parent or guardian’s signature. If they are unable to sign, they may place their fingerprint or a cross on the consent form.

For patients unable to read or understand English or the local dialect, the patient information leaflet and consent form will be read out and explained in the parent/guardian’s local dialect in the presence of a witness who will also sign the consent form to confirm that the parent/guardian has freely given his/her consent.

Only one informed consent will be signed at the patient’s inclusion into the cohort, the duration of the study (including passive follow-up) will be explained to parent/guardian. Prior to include a patient in a new episode follow-up, an oral consent has to be obtained from parent/guardian.

The person discussing this information with the patient will also sign and date the consent form.

## Institutional Review Board/Independent Ethics Committee (IRB/IEC)

The Investigator or the Sponsor must submit this Clinical Trial Protocol to the appropriate Ethics Committee (the Faculty of Medicine Makerere University Research and Ethics Committee (FOMREC), Uganda National Council of Science and Technology (UNCST), the Uganda National Drug Authority, (NDA)), and is required to forward to the Sponsor a copy of the written and dated approval/favorable opinion signed by the Chairman with Ethics Committee (FOMREC, UNCST and NDA). In compliance with international recommendations, a similar protocol performed in Senegal (including children and adults and with a 28 day follow-up) has been submitted for consultation to a French IEC.

The Clinical Trial (study number, Clinical Trial Protocol title and version number), the documents reviewed (Clinical Trial Protocol, Informed Consent Form, Summary of Product Characteristics, Investigator’s CV, etc.), the list of voting members and their qualifications and the date of the review should be clearly stated on the written (FOMREC) approval/favorable opinion.

Investigational Product will not be released at the study site and the Clinical Trial will not start until a copy of this written and dated approval/favorable opinion has been received by the Sponsor.

During the Clinical Trial, any amendment or modification to the Clinical Trial Protocol should be submitted to the Faculty of Medicine Makerere University Research and Ethics Committee (FOMREC), Uganda National Council of Science and Technology (UNCST), the Uganda National Drug Authority, (NDA). It should also be informed of any event likely to affect the safety of patients or the continued conduct of the Clinical Trial, in particular any change in safety. All updates to the Investigator’s Brochure will be sent to the Faculty of Medicine Makerere University Research and Ethics Committee (FOMREC), Uganda National Council of Science and Technology (UNCST), the Uganda National Drug Authority, (NDA?.

A progress report shall be sent to the Faculty of Medicine Makerere University Research and Ethics Committee (FOMREC), Uganda National Council of Science and Technology (UNCST), the Uganda National Drug Authority, annually or as prescribed and a summary of the Clinical Trial’s outcome at the end of the Clinical Trial. Any serious adverse reactions and/or unanticipated effects on subjects which may occur as a result of this study shall be immediately reported to the FOMREC, UNCST and NDA. The FOMREC Continuing annual Review Form shall be completed and submitted annually to the review board (when due) as well as the Final/Study termination form at the end of the proposed study . The final study report shall be submitted to the FOM-REC using a standard form.

# Study monitoring

## Responsibilities of the Investigator(s)

The Investigator(s) undertake(s) to perform the Clinical Trial in accordance with this Clinical Trial Protocol, ICH guidelines for Good Clinical Practice and the applicable regulatory requirements.

The Investigator is required to ensure compliance with all procedures required by the Clinical Trial Protocol and with all study procedures provided by the Sponsor (including security rules). The Investigator agrees to provide reliable data and all information requested by the Clinical Trial Protocol (with the help of the Case Report Form [CRF], Discrepancy Resolution Form [DRF] or other appropriate instrument) in an accurate and legible manner according to the instructions provided and to ensure direct access to source documents by Sponsor representatives.

The Investigator may appoint such other individuals as he may deem appropriate as Sub-Investigators to assist in the conduct of the Clinical Trial in accordance with the Clinical Trial Protocol. All Sub-Investigators shall be appointed and listed in a timely manner. The Sub-Investigators will be supervised by and under the responsibility of the Investigator. The Investigator will provide them with a copy of the Clinical Trial Protocol and all necessary information.

## Responsibilities of the Sponsor

The Sponsor of this Clinical Trial is responsible to Health Authorities for taking all reasonable steps to ensure the proper conduct of the Clinical Trial Protocol as regards ethics, Clinical Trial Protocol compliance, and integrity and validity of the data recorded on the Case Report Forms. Thus, the main duty of the Monitoring Team is to help the Investigator and the Sponsor maintain a high level of ethical, scientific, technical and regulatory quality in all aspects of the Clinical Trial.

At regular intervals during the Clinical Trial, the site will be contacted, through monitoring visits, letters, emails or telephone calls, by a representative of the Monitoring Team to review study progress, Investigator and patient compliance with Clinical Trial Protocol requirements and any emergent problems. During these monitoring visits, the following but not exhaustive list of points will be scrutinized with the Investigator: patient informed consent, patient eligibility, patient recruitment and follow-up, Serious Adverse Event documentation and reporting, Investigational Product allocation, patient compliance with the Clinical Trial Protocol and the Investigational Product regimen, Investigational Product accountability, concomitant therapy use and quality of data.

## Source document requirements

According to the ICH guidelines for Good Clinical Practice, the Monitoring Team must check the Case Report Form entries against the source documents, except for the pre-identified source data directly recorded in the Case Report Form. The Informed Consent Form will include a statement by which the patient allows the Sponsor’s duly authorized personnel, the Faculty of Medicine Makerere University Research and Ethics Committee (FOMREC), Uganda National Council of Science and Technology (UNCST), the Uganda National Drug Authority, (NDA) to have direct access to source data which support the data on the Case Report Forms (eg, patient's medical file, appointment books, original laboratory records, etc.). Such personnel, bound by professional secrecy, must keep confidential all personal identity or personal medical information (according to confidentiality rules).

## Use and completion of Case Report Forms (CRFs) and additional request

It is the responsibility of the Investigator to maintain adequate and accurate CRFs (according to the technology used) designed by the Sponsor to record (according to Sponsor instructions) all observations and other data pertinent to the clinical investigation. All CRFs should be completed in their entirety in a neat, legible manner to ensure accurate interpretation of data.

Should a correction be made, the information to be modified must not be overwritten. The corrected information will be transcribed by the authorized person next to the previous value, initialed and dated.

The collection of the CRF will be done by the monitor after the whole completion of the CRF.

The computerized handling of the data by the Sponsor after receipt of the CRFs may generate additional requests (DRF) to which the Investigator is obliged to respond by confirming or modifying the data questioned. The requests with their responses will be appended to the CRFs held by the Investigator and the Sponsor.

# Administrative rules

## Curriculum Vitae

An updated copy of the curriculum vitae limited to the experience, qualification and training for each Investigator and Sub-Investigator will be provided to the Sponsor prior to the beginning of the Clinical Trial.

## Record retention in study site(s)

The Investigator must maintain confidential all study documentation, and take measures to prevent accidental or premature destruction of these documents.

It is recommended that the Investigator retain the study documents at least fifteen (15) years after the completion or discontinuation of the Clinical Trial, unless otherwise specified in the Investigator Agreement in line with national standards and/or local laws.

However, applicable regulatory requirements should be taken into account in the event that a longer period is required.

The Investigator must notify the Sponsor prior to destroying any study essential documents following the Clinical Trial completion or discontinuation.

If the Investigator's personal situation is such that archiving can no longer be ensured by him/her, the Investigator shall inform the Sponsor and the relevant records shall be transferred to a mutually agreed upon designee.

# Confidentiality

All information disclosed or provided by the Sponsor (or any company/institution acting on their behalf), or produced during the Clinical Trial, including, but not limited to, the Clinical Trial Protocol, the CRFs, and the results obtained during the course of the Clinical Trial, is confidential. The Investigator and any person under his/her authority agree to undertake to keep confidential and not to disclose the information to any third party without the prior written approval of the Sponsor.

However, the submission of this Clinical Trial Protocol and other necessary documentation to the Faculty of Medicine Makerere University Research and Ethics Committee (FOMREC), Uganda National Council of Science and Technology (UNCST), the Uganda National Drug Authority, (NDA)is expressly permitted, their members having the same obligation of confidentiality.

The Sub-Investigators shall be bound by the same obligation as the Investigator. The Investigator shall inform the Sub-Investigators of the confidential nature of the Clinical Trial.

The Investigator and the Sub-Investigators shall use the information solely for the purposes of the Clinical Trial, to the exclusion of any use for their own or for a third party's account.

# Property rights

All information, documents and Investigational Product provided by the Sponsor or its designee are and remain the sole property of the Sponsor.

The Investigator shall not mention any information or the Product in any application for a patent or for any other intellectual property rights.

All the results, documents and inventions, which arise directly or indirectly from the Clinical Trial in any form, shall be the exclusive property of the Sponsor.

The Sponsor may use or exploit all the results at its own discretion, without any limitation to its property right (territory, field, continuance). The Sponsor shall be under no obligation to patent, develop, market or otherwise use the results of the Clinical Trial.

# Data protection

The patient's personal data and Investigator's personal data which may be included in the Sponsor database shall be treated in compliance with all applicable laws and regulations.

When archiving or processing personal data pertaining to the Investigator and/or to the patients, the Sponsor shall take all appropriate measures to safeguard and prevent access to this data by any unauthorized third party.

# Insurance compensation

The Sponsor certifies that it has taken out a liability insurance policy covering all clinical trials under its sponsorship. This insurance policy is in accordance with local laws and requirements. The insurance obtained by the sponsor does not relieve the Investigator and the collaborators from maintaining their own liability insurance policy. A copy of the insurance certificate will be provided to the Ethic Committees/IRBs or CA in countries requiring this documentation.

# Sponsor audits and inspections by regulatory agencies

For the purpose of ensuring compliance with the Clinical Trial Protocol, Good Clinical Practice and applicable regulatory requirements, the Investigator should permit auditing by or on the behalf of the Sponsor and inspection by applicable regulatory authorities.

The Investigator agrees to allow the auditors/inspectors to have direct access to his/her study records for review, being understood that this personnel is bound by professional secrecy, and as such will not disclose any personal identity or personal medical information.

The Investigator will make every effort to help with the performance of the audits and inspections, giving access to all necessary facilities, data, and documents.

As soon as the Investigator is notified of a future inspection by the authorities, he will inform the Sponsor and authorize the Sponsor to participate in this inspection.

The confidentiality of the data verified and the protection of the patients should be respected during these inspections.

Any result and information arising from the inspections by the regulatory authorities will be immediately communicated by the Investigator to the Sponsor.

The Investigator shall take appropriate measures required by the Sponsor to take corrective actions for all problems found during the audit or inspections.

# Premature discontinuation of the study or premature close-out of a site

## Decided by the Sponsor in the following cases:

If the information on the product leads to doubt as to the benefit/risk ratio;

If the Investigator has received from the Sponsor all Investigational Product, means and information necessary to perform the Clinical Trial and has not included any patient after a reasonable period of time mutually agreed upon;

In the event the results of the Clinical Trial do not appear to be scientifically convincing to the Sponsor (for example based on the results of a planned futility analysis);

If the aim of the Clinical Trial has become outdated or is no longer of interest;

In the event of breach by the Investigator of a fundamental obligation under this agreement, including but not limited to breach of the Clinical Trial Protocol, breach of the applicable laws and regulations or breach of the ICH guidelines for Good Clinical Practice;

In any case the Sponsor will notify the Investigator of its decision by written notice.

## Decided by the Investigator

The Investigator must notify (at least 30 days prior to discontinuation) the Sponsor of his/her decision and give the reason in writing.

In all cases (decided by the sponsor or by the Investigator), the Faculty of Medicine Makerere University Research and Ethics Committee (FOMREC), Uganda National Council of Science and Technology (UNCST), the Uganda National Drug Authority, (NDA) should be informed.

# Clinical trial results

The Sponsor will be responsible for preparing a Clinical Study Report;

When the data from all investigational sites have been fully analyzed by the Sponsor, the latter will communicate the results of the Clinical Trial to the Investigator(s);

Regardless of the study outcome the Sponsor is committed to publish the results.

# Publications and communications

## Publication/communication of study results

The Sponsor recognizes the Investigator's right to utilize data derived from the clinical trial for teaching purposes, communication at congresses and scientific publications. Nevertheless, in order to ensure the accuracy and scientific value of the information, while preserving the independence and accountability of the Investigator, and the confidentiality of the information, only checked and validated data will be used. To that effect, it is essential that the parties exchange and discuss, prior to any publication or communication, any draft publication or communication made by the Investigator.

The Investigator shall send to the Sponsor a copy of the manuscript for review and possible comments at least forty-five (45) calendar days in advance of the date of submission to the journal and at least twenty (20) days in advance for abstracts. The publication shall be delayed until a written response is received by the Sponsor, not to exceed ninety (90) days The Sponsor can delay publication or communication for a limited time in order to protect the confidentiality or proprietary nature of any information contained therein, it being understood that the Sponsor cannot refuse its consent without reasonable cause. The Investigator agrees to include the modifications requested by the Sponsor, provided they do not jeopardize the accuracy and/or the scientific value of the publication.

The study results must be submitted to the review of the Steering Committee before publication. All study investigators and Committee members give full authority to the Steering Committee for primary presentation and/or primary publication of results. However in absence of primary publication within 12 months of the termination of the clinical trial at all other sites, the Sponsor or the Steering Committee if applicable may consider investigator’s request for independent publication..The Investigator agrees not to publish the results of the clinical trial pertaining to his/her center prior to the publication of the overall clinical trial results. If no publication has occurred within twelve (12) months of the termination of the clinical trial at all other sites, the Investigator shall have the right to publish independently the results of this clinical trial, patient to the review procedure set forth herein. If the clinical trial is conducted with the support of a Steering Committee, the latter may define specific rules for publication.

No other publication is allowed before the primary publication. Any subsequent presentation or publication by a study participant (including for substudies) must be approved by the Sponsor and make reference to the study and the primary publication.

The Investigator shall not use the name(s) of the Sponsor and/or its employees in advertising or promotional material or publication without the prior written consent of the Sponsor. The Sponsor shall not use the name(s) of the Investigator and/or the collaborators in advertising or promotional material or publication without having received his/her and/or their prior written consent(s).

The Sponsor has the right at any time to publish the results of the clinical trial.

## Public disclosure of clinical trials

Regarding clinical trial registries, sanofi-aventis will comply with the following:

- This clinical trial will be recorded in a registry accessible to the public free of charge; Sanofi-aventis has decided to register its clinical trials on the following web site: [www.clinicaltrials.gov](javascript:go_out_ext('http://www.clinicaltrials.gov');).
  - The registry will contain basic information about each trial sufficient to inform interested patients (and their healthcare practitioners) how to enrol in the trial;
  - The trial will be registered under a unique identification number to ensure transparency of clinical trial results;
  - As a general rule, clinical trial information will be published within 21 days following enrolment of the first patient.
- The results of this clinical trial will be published in a database accessible to the public free of charge; Sanofi-aventis has decided to publish the results of its clinical trials on the following web site: [www.clinicalstudyresults.org](javascript:go_out_ext('http://www.clinicalstudyresults.org');)
  - If trial results are published in a peer-reviewed medical journal, the database will contain a citation to or link to the journal article and/or a summary of the results in a standard format, such as the ICH E-3 summary format, that includes a description of the trial design and methodology, results of primary and secondary outcome measures, and safety results. Study results will be recorded in a database in the ICH E-3 format if they are not published in a medical journal within required time frame;
  - Results will include the unique identification number used to register the corresponding trial;
  - As a general rule, results will be published within one year of trial completion, unless such publication would compromise publication in a peer-reviewed medical journal or contravene national laws or regulations.

In case of local requirement, additional registration at local registries may also be done by sanofi-aventis.

# Clinical trial protocol amendments

All appendices attached hereto and referred to herein are made part of this Clinical Trial Protocol.

The Investigator should not implement any deviation from, or changes of the Clinical Trial Protocol without agreement by the Sponsor and prior review and documented approval/favorable opinion from the Faculty of Medicine Makerere University Research and Ethics Committee (FOMREC), Uganda National Council of Science and Technology (UNCST), the Uganda National Drug Authority, (NDA)of an amendment, except where necessary to eliminate an immediate hazard(s) to Clinical Trial Patients, or when the change(s) involves only logistical or administrative aspects of the trial. Any change agreed upon will be recorded in writing, the written amendment will be signed by the Investigator and by the Sponsor and the signed amendment will be filed with this Clinical Trial Protocol.

Any amendment to the Clinical Trial Protocol requires written approval/favorable opinion by the Faculty of Medicine Makerere University Research and Ethics Committee (FOMREC), Uganda National Council of Science and Technology (UNCST), the Uganda National Drug Authority, (NDA) prior to its implementation, unless there are overriding safety reasons.

In some instances, an amendment may require a change to the Informed Consent Form. The Investigator must receive an approval/favorable opinion from the Faculty of Medicine Makerere University Research and Ethics Committee (FOMREC), Uganda National Council of Science and Technology (UNCST), the Uganda National Drug Authority, (NDA) concerning the revised Informed Consent Form prior to implementation of the change

# Bibliographic references

1 World Health Organization. Antimalarial drug combination therapy: report of a technical consultation. Geneva: WHO, 2001: 33.

2 Adjuik M, Agnamey P, Babiker A, et al.: Amodiaquine-artesunate versus amodiaquine for uncomplicated Plasmodium falciparum malaria in African children: a randomised, multicentre trial. Lancet 2002; 359: 1365–72.

3 Brasseur P, Agnamey P, Eldin de Pecoulas P, Faucher JF, Cisse M, Gaye O, Sokhna C, Trape JF, Oliaro P: Efficacy, safety and pharmaco-vigilance of artesunate-amodiaquine combination for treatment of uncomplicated malaria in Casamance, Senegal; MIM conference Nov 2005

4 Ndiaye JL, Faye B, Ndiaye D, Dieng T, Bah IB, Dieng Y, Faye O, Ndir O, Gaye O: Management of uncomplicated malaria attacks by combinations therapies artemisin-based in Senegal, West Africa; American Journal of Trop Med and Hyg, 2004 vol l71

5 Tall A, Rabarijaona LP, Ariey F, Robert V, et al: Efficacy of artesunate+amodiaquine, artesunate+ sulfadoxine-pyrimethamine, chloroquine+ sulfadoxine-pyrimethamine in P.falciparum malaria in Comoros; MIM conference Nov 2005

6 Bakary F, Djimde A, Sidibe B, Dembele D,et al: Comparative efficacy , safety and tolerability of three treatment regiments for uncomplicated falciparum malaria: artesunate+amodiaquine vs artesunate+sulfadoxine-pyrimethamine,vs artesunate; American Journal of Trop Med and Hyg, 2004 vol l71

7 Sirima et al., International congress of Tropical medicine and International Health, Amsterdam, 2007

8 Mutabingwa TK, Heller A, Hallett R, Ahmed J, Drakeley C, Greenwood B, Whitty C: Amodiaquine alone, amodiaquine_sulfadoxinepyrimethamine, amodiaquine_artesunate, andartemether-lumefantrine for outpatient treatment of malaria in Tanzanian children: a four-arm randomised effectiveness trial; Lancet 2005; 365: 1474–80

9 Piola P, Fogg C, Bajunirwe F, BiraroS, et al: Supervised versus unsupervised intake of six-dose artemether-lumefantrine for treatment of acute, uncomplicated Plasmodium falciparum malaria in Mbarara, Uganda: a randomised trial: Lancet 2005; 365: 1467–73

10. Omari AA, Gamble C, Garner P (2004). Artemether-Lumefantrine for uncomplicated malaria: a systematic review. Trop Med Int Health 9: 192-199.

11. The East African Network for Monitoring Antimalarial Treatment (EANMAT) The efficacy of antimalarial monotherapies sulphadoxine-pyrimethamine and amodiaquine in East Africa: implications for sub-regional policy. Trop. Med. Int. Health. 2003; 8: 860-67.

12. Talisuna A, Nalunkuma-Kazibwe A, Bakyaita N, Langi P, Mutabingwa TK, Watkins WM, Van Marck E, D’Alessandro U, Egwang. TG. Efficacy of Sulphadoxine-pyrimethamine alone or combined with Amodiaquine or Chloroquine for the treatment of uncomplicated falciparum malaria in Ugandan children Trop. Med. Int. Health. 2004; 9:222-9.

13. Okello PE, Van Bortel W, Byaruhanga AM, Correwyn A, Roelants P, et al. (2006) Variation in malaria transmission intensity in seven sites throughout Uganda. Am J Trop Med Hyg 75: 219-225.

# Appendices

APPENDIX 1: WHO guidelines for assessing response to treatment (2003)

APPENDIX 2: Guidelines for grading physical examination findings

APPENDIX 3: Guidelines for grading laboratory results

APPENDIX 4: Criteria for severe malaria / Danger signs

APPENDIX 5: Specific guideline for neutropenia and increase of ALT

APPENDIX 6: Methods of blood sample analyzes

APPENDIX 7: Operational definitions

APPENDIX 8: World Medical Association Declaration of Helsinki

APPENDIX 9: Flow of patients in OPD and Study Clinic

APPENDIX 1Document related to the assessment of judgment criteria

WHO guidelines for assessing response to treatment (2003)

| ETF (Early Treatment Failure):   - Development of danger signs or severe malaria on Days 1or 2 or 3 in the presence of parasitemia - Parasitemia on Day 2 higher than on Day 0, irrespective of axillary temperature - Parasitemia on Day 3 with axillary temperature > 37,5°C - Parasitemia on Day 3 > 25% of count on Day 0 |
| --- |
| LCF (Late Clinical Failure):   - Development of danger signs or severe malaria after Day 3 in the presence of parasitemia, without previously meeting any of the criteria of early treatment failure - Axillary temperature > 37,5°C ( or history of fever) on any day from Day 4 to 28 in the presence of parasitemia, without previously meeting any of the criteria of early treatment failure |
| LPF ( Late Parasitological Failure):   - Presence of parasitemia on any day from Day 7 to Day 28 and axillary temperature  < 37,5°C , without previously meeting any of the criteria of early treatment failure or late clinical failure |
| **ACPR (Adequate Clinical and Parasitological Response)**   - Absence of parasitaemia on Day 28, irrespective of axillary temperature, without previously meeting any of the criteria of early treatment failure or late clinical or parasitological failure |

Appendix 2: Guidelines for grading physical examination findings

|  | **Grade 1**  **MILD** | **Grade 2**  **MODERATE** | **Grade 3**  **SEVERE** | **Grade 4**  **LIFE THREATENING** |
| --- | --- | --- | --- | --- |
| **Subjective fever in the past 24 h** | N/A | Present (Yes) | N/A | N/A |
| ***Weakness*** | Mild decrease in activity, still playing | Moderate decrease in activity, playing limited | Not participating in usual activities, not playing | Lethargic |
| **Muscle and/or joint aches*** | Mild localised complaints | Mild diffuse complaints | Objective weakness; function limited | N/A |
| ***Headache**** | Mild, no therapy required | Transient, moderate; therapy required | Severe; responds to initial narcotic therapy | Intractable; requires repeated narcotic therapy |
| **Anorexia** | Decreased appetite, but still taking solid food | Decreased appetite, avoiding solid food | Refusing to breast feed, appetite very decreased, no solids or liquids taken  (< 2 years < 12 hr;  > 2 years < 24 hr) | Refusing to breast feed, appetite very decreased, no solids or liquids taken  (< 2 years < 12 hr; > 2 years < 24 hr) |
| **Nausea*** | Mild discomfort; maintains reasonable intake | Moderate discomfort; intake decreased significantly; some activity limited | Severe discomfort; no significant intake; activities limited | Minimal fluid intake |
| **Vomiting** | Transient emesis | Occasional or moderate vomiting | Orthostatic hypotension or IV fluids required | Hypotensive shock or hospitalization required for IV fluid |
| **Abdominal pain*** | Mild | Moderate – no treatment needed | Moderate to severe – treatment needed | Severe – hospitalized for treatment |
| **Diarrhea** | Transient 3-4 loose stools/day | 5-7 loose stools/day | Orthostatic hypotension or > 7 loose stools/day or IV fluids required | Hypotensive shock or hospitalization for IV fluid therapy required |
| **Cough** | Transient – no treatment required | Continuous, requires treatment | Uncontrolled | Cyanosis, stridor, severe shortness of breath |
| **Pruritis** | Pruritis without rash | Pruritic rash, pruritis without rash that disturbs sleep | Mild urticaria | Severe urticaria, anaphylaxis, angioedema |
| **Tinnitus*** | Mild ringing or roaring sound | Moderate ringing or roaring sound | Severe ringing or roaring sound with associated hearing loss | N/A |
| **Behavioural changes** | Mild difficultty concentrating; mild confusion or agitation; activities of daily living unaffected; no treat^t^ | Moderate confusion or agitation; some limitation of activities of daily living; minimal treatment | Severe confusion or agitation; Needs assistance for activities of daily living; therapy required | Toxic psychosis; hospitalization required |
| **“Flu”**  **(viral URI)** | Mild nasal congestion, mild rhinorrhea, no cough | Moderate nasal congestion, moderate rhinorrhea, cough present | N/A (if severe, classify individual symptoms) | N/A (if life-threatening, classify individual symptoms) |
| **Convulsion** | N/A | N/A | Localized or generalized seizure | Status epilepticus |
| *** Assess only in children > 3 years of age. Answer N/A for younger children and those unable to answer.** | | | | |

† Reference – Based on WHO Toxicity Grading Scale for Determining the Severity of Adverse Events

**APPENDIX 2b Guidelines for grading physical examination findings (cont.)**

|  | **Grade 1**  **MILD** | **Grade 2**  **MODERATE** | **Grade 3**  **SEVERE** | **Grade 4**  **LIFE-THREATENING** | |
| --- | --- | --- | --- | --- | --- |
| **Temperature* (tympanic membrane)** | 38.0-38.4°C | 38.5-40.0°C | > 40.0°C | Sustained fever, equal or greater than 40.0°C for longer than 5 days | |
| **Dehydration**  ****** | Normal skin turgor and touch, moist mucous membranes, tears present, eyes normal, fontanelle flat, CNS – consolable, pulse regular, urine output normal | Skin dry with + tenting, dry mucous membranes, eyes deep set, decreased tears, fontanelle soft, CNS – irritable, pulse slightly increased, urine output decreased | Skin clammy with lack of turgor, parched/cracked mucous membranes, sunken eyes, no tears, sunken fontanelle, CNS – lethargic, pusle increased, no urine output | | |
| **Facial edema** | Present, mild swelling of eyes | Moderate swelling of eyes, face | Severe swelling involving eyes, face, and mucous membranes; unable to open eyes | | Airway compromise |
| **Jaundice** | Slight yellowing of sclera and conjunctiva | Moderate yellowing of sclera and conjunctiva, yellowing of mucous membranes | Severe yellowing of sclera and conjunctiva, yellowing of skin | | N/A |
| **Chest** | Mildly increased RR (for age, temperature), transient or localised adventitious sounds | Moderately increased RR, diffuse or persistent adventitious sounds | Rapid RR (< 2 months > 60, 2-12 months > 50,  1-5 years > 40, adults > 30)*nasal flaring, retractions | | Cyanosis |
| **Abdomen** | Normal bowel sounds, mild localised tenderness, and/or liver palpable 2-4 cm below the right costal margin (RCM), and/or spleen palpable, and/or umbilical hernia present | Normal or mildly abnormal bowel sounds, moderate or diffuse tenderness; and/or mild to moderately enlarged liver (4-6 cm below the RCM) and/or spleen palpable up to half-way between umbilicus and symphysis pubis | Severely abnormal bowel sounds, severe tenderness to palpation. Evidence of peritoneal irritation and/or significant enlargement of liver (> 6 cm below the RCM) and/or spleen palpable beyond half-way between umbilicus and symphysis pubis | | Absent bowel sounds. Involuntary rigidity |
| **Skin†** | Localised rash, erythema, or pruritis | Diffuse, maculopapular rash, dry desquamation | Vesiculation, moist desquamation, or ulceration | | Exfoliative dermatitis, mucous membrane involvement or erythema multiforme or suspected Stevens-Johnson or necrosis requiring surgery |

*** Reference - DMID Pediatric Toxicity Tables, May 2001, Drug Fever (Rectal)**

**** Reference – The Harriet Lane Handbook, 15^th^ edition, 2000**

**† Reference – WHO Toxicity Grading Scale for Determining the Severity of Adverse Events**

**APPENDIX 2c :** **Guidelines for grading physical examination findings (cont.)**

|  | **Grade 1**  **MILD** | **Grade 2**  **MODERATE** | **Grade 3**  **SEVERE** | **Grade 4**  **LIFE-THREATENING** |
| --- | --- | --- | --- | --- |
| **Hearing** | *< 4 years: N/A*  > 4 years: Decreased hearing in one ear | *< 4 years: N/A*  > 4 years: Decreased hearing in both ears or severe impairment in one ear | *< 4 years: Any evidence of hearing impairment*  > 4 years: Severe impairment in both ears | N/A |
| **Tablet test** | Difficulty grasping tablet but able to pick up | Unable to pick up tablet without dropping | Unable to grasp tablet | N/A |
| **Clinical symptoms/sign** *(not otherwise specified)* | No therapy; monitor condition | May require minimal intervention and monitoring | Requires medical care and possible hospitalization | Requires active medical intervention, hospitalization, or hospice care |

**APPENDIX 2d :**

**Guidelines for grading splenic enlargement.**

Hackett splenic score (score from 0 to 5)

0 = spleen not palpable even on deep inspiration

1= spleen palpable on deep inspiration

2 = spleen palpable on normal inspiration, but not beyond a horizontal line half way between the costal margin and umbilicus, measured in a line dropped vertically from the left nipple

3 = spleen extending below this line, but not below a line running horizontally through the umbilicus

4 = spleen extending below this line, but not below a horizontal line half way between the umbilicus and pubic symphysis

5 = spleen extending below this line.

**APPENDIX 3**

**GUIDELINES FOR GRADING LABORATORY RESULTS: paediatric toxicity tables (greater than 3 months of age)**

|  | **Grade 1**  **MILD** | **Grade 2**  **MODERATE** | **Grade 3**  **SEVERE** | **Grade 4**  **LIFE-THREATENING** |
| --- | --- | --- | --- | --- |
| **Absolute neutrophil count *(/****mm^3^****)*** | 750 –1200 | 400 –749 | 250 –399 | <250 |
| **Hemoglobin**  ***(****g/dL)*  *age >3months and < 2 years* | 9.0 – 9.9 | 7.0 – 8.9 | <7.0 | Cardiac failure secondary to anemia |
| **Hemoglobin**  ***(****g/dL)*  *age >2 years* | 10 –10.9 | 7.0 –9.9 | <7.0 | Cardiac failure secondary to anemia |
| **Platelets *(/****mm^3^****)*** | N/A | 50,000 –75,000 | 25,000 –49,999 | <25,000 |
| **ALT *(U/L)*** | 1.1-4.9 x ULN | 5.0-9.9 x ULN | 10.0-15.0 x ULN | >15.0 x ULN |
| **AST *(U/L)*** | 1.1-4.9 x ULN | 5.0-9.9 x ULN | 10.0-15.0 x ULN | >15.0 x ULN |
| **Bilirubin** | 1.1-1.9 x ULN | 2-2.9 x ULN | 3.0-7.5 x ULN | >7.5 x ULN |
| **Creatinine**  3 months to 2 years of age | 0.6–0.8 x ULN | 0.9–1.1 x ULN | 1.2–1.5 x ULN | >1.5 x ULN |
| 2 to 12years of age | 0.7–1.0 x ULN | 1.1–1.6 x ULN | 1.7–2.0 x ULN | >2.0 x ULN |
| *Age>12 years* | *1.0–1.7 x ULN* | *1.8–2.4 x ULN* | *2.5–3.5 x ULN* | *>3.5 x ULN* |
| **Laboratory values**  *(not otherwise specified)* | Abnormal but requiring no immediate intervention; follow | Sufficiently abnormal to require evaluation as to causality and perhaps mild therapeutic intervention | Sufficiently severe to require evaluation and treatment | Life-threatening severity; requires immediate evaluation, treatment, and usually hospitalization |

**Reference – DMID Pediatric Toxicity Tables, May 2001**

**APPENDIX 4**

**Criteria for Severe Malaria/Danger Signs (WHO, 2000)**

**Severe Malaria**

- prostration
- Unarousable coma *(if after convulsion, > 30 min)*
- Repeated convulsions *(> 2 within 24 h)*
  - Respiratory distress *(laboured breathing at rest)*
  - circulatory collapse
  - pulmonary oedema (radiological)
  - abnormal bleeding
  - jaundice
  - haemoglobinurea
  - Severe anaemia *(Hb < 5.0 g/dL)*
  - hypoglycemia
  - acidosis
  - hyperlactataemia
  - hyperparasitemia
  - renal impairment

**Danger Signs**

- Recent convulsions *(>1 within 24 h)*
- Altered consciousness  *(confusion)*
- Lethargy
  - Unable to drink or breast feed
  - Vomiting everything
  - Unable to stand/sit due to weakness

**APPENDIX 5 a**

*.*

**NEUTROPENIA**

| Neutrophils < 400/mm^3^ |
| --- |

| Repeat immediately a full blood count |
| --- |

| Neutrophils < 400/mm^3^ confirmed  with signs of infection |  | Neutrophils < 400/mm^3^ confirmed  with no sign of infection |
| --- | --- | --- |

| 1. DISCONTINUE Investigational Product, hospitalization should be considered. |  | 1. DISCONTINUE Investigational Product |
| --- | --- | --- |
| 1. PERFORM biological investigations for infection |  | 1. INVESTIGATE for etiology |

in both situations

| 1. INFORM the sponsor |
| --- |
| 1. INVESTIGATE previous treatments, particularly long-term, even a long time ago, exposure to toxic agents, e.g. benzene, X-rays, etc. |
| 1. PERFORM and collect the following investigations (results):  - RBC and platelet counts - Serology: EBV, (HIV), mumps, measles, rubella |
| 1. DECISION for potential bone marrow aspiration: to be taken in specialized unit |
| 1. FREEZE serum (5 mL x 2) on Day 1 (cessation of Investigational Product) and Day 5 (For further investigations). |
| 1. MONITOR the leukocyte and neutrophil counts 3 times per week for at least one week, then twice a month until it returns to normal, |

**APPENDIX 5 b**

**INCREASE IN ALANINE AMINOTRANSFERASES**

| Increase in  ALT (SGPT) |
| --- |

| > 10 ULN |
| --- |

| Repeat immediately the count  if confirmed |
| --- |

| 1. DISCONTINUE administration of the Investigational Product 2. HOSPITALIZATION should be considered if ALT ≥ 10 ULN and/or jaundice or coagulation disorder (PT <50% with factor V <50%) or signs of hepatic encephalopathy 3. INFORM the sponsor 4. INTERVIEW patient again about consumption of alcohol, drugs and herbals received *before* and *during* the trial and possible contamination by HAV, HBc or HCV virus in the last six months (blood or blood product transfusion, intravenous drug addiction) 5. INVESTIGATE for other etiology | 1. PERFORM the following examinations :  - Complete blood count and LFTs - Serum creatinine - Anti-HIV IgM, anti HBc IgM, anti-HCV IgM, anti-CMV IgM - Specific serologic markers of recent infection with ***** EBV, herpes viruses and toxoplasma (depending on the clinical context) ***** hepatobiliary ultrasonography  1. FREEZE serum (5 mL x 2) 2. MONITOR LFTs (including aminotransferases) every 3 days for the first week then once weekly until return to normal or for at least 3 months |
| --- | --- |

**APPENDIX 6:**

**Methods of blood sample analyzes**

The procedures describing in detail the blood sampling methods, storage and analysis of samples will be described in an operating manual validated by the investigator and filed in the site.

The apparatus used for the determinations must have a regular quality control system.

Each tube/ slide will be identified with the patient inclusion number together with the date and visit day.

**1. Haematological analyses**

The haematological analysis will consist of a haemoglobin determination and a complete blood count (CBC) in order to measure leukocytosis and platelet count. Hemoglobin for screening patients will be measured from fingerprick blood samples using a portable spectrophotometer (HemoCue, Anglom, Sweden). Other haematological measurements shall be done by a haematology coulter.

**2. Biochemical analyses**

The following biochemical parameters will be analysed: hepatic enzymes (ALT), total bilirubine, blood creatinine, and blood glucose. In the event of abnormal values, a control sample will be performed.

**3 Parasitological analyses**

Screening thick smears will be stained with 10% Giemsa for 10 minutes. Study thick and thin blood smears will be stained with 2% Giemsa for 30 minutes. Thick blood smears will be evaluated for the presence of parasitemia (asexual forms only) and gametocytes. Parasite and gametocyte densities will be calculated from thick blood smears by counting the number of asexual parasites and gametocytes, respectively, per 200 leukocytes (or per 500, if the count is <10 parasites or gametocytes/200 leukocytes), assuming a leukocyte count of 8,000/μl. A thick blood smear will be considered negative when the examination of 100 high power fields does not reveal asexual parasites or gametocytes. Thin blood smears will be evaluated to determine parasite species.

**4. Molecular biology studies**

Blood samples will be collected from patients on days 0, 2, 3, 7, 14, 21, 28, 42 and on any unscheduled day that the patient presents with clinical deterioration or recurrent fever. Blood will be placed onto filter paper in approximately 25 ul aliquots per blood spot. The samples will be labelled, air-dried and stored in small, sealed sample bags at ambient temperature with desiccant. Parasite DNA will subsequently be removed from the filter paper and prepared for molecular analysis using a chelex extraction method. Genotyping will be performed on all patients with outcomes classified as LCF or LPF. Genotyping of parasites collected at baseline (day 0) and the day of recurrent parasitemia will be done to distinguish between true recrudescence and new infections.

Genotyping of the recurrent infection will be done by characterizing MSP1, MSP2 and GLURP, single-copy genes in the Plasmodium falciparum genome. PCR-amplification of DNA from a single parasite clone results in a single amplification product. For the three genes, each PCR-amplification product of a different size is considered to originate from a different clone of Plasmodium falciparum and reflects a different genotype. For the samples collected from the same patient at day 0 and day of recurrent parasitaemia, the length polymorphism of MSP1, MSP2 and GLURP will be determined. Selected regions of the MSP1, MSP2 and GLURP will be amplified using PCR and characterized based on sequence and size polymorphisms identified by gel electrophoresis. Genotyping patterns on the day of recurrent parasitemia will be compared with those at treatment initiation using GelCompar II software (Applied Maths). This laboratory work will be performed in Kampala. Results will be interpreted as follows:

Recrudescence: For each marker (MSP1, MSP2 and GLURP), at least one identical length polymorphism is found in the sample collected at day 0 and day of recurrent parasitaemia.

New infection: For at least one marker, length polymorphism is different between the sample collected at day 0 and that at day of recurrent parasitaemia.

Indeterminate: Samples that failed to produce a result due to an inability to amplify DNA at day 0 and/or day of recurrent parasitaemia.

**APPENDIX 7**

**OPERATIONAL DEFINITIONS.**

**Audit:** A systematic examination, carried out independently of those directly involved in the clinical trial to determine whether the conduct of the trial complies with the protocol and whether the data reported are consistent with the records on the site.

**Case Report form (CRF)**: A paper or electronic document used to record data on each trial subject during the trial, as defined by the protocol.

**Clinical Trial:** A systemic study on pharmaceutical products in human subjects, performed in order to discover or verify the effects of and/identity any adverse reactions to investigational products, and/or to study the absorption, distribution, metabolism, and excretion of the products with the object of ascertaining their efficacy and safety.

**Compliance:** Adherence to all the research related requirements, Good Clinical Practice (GCP) requirements, and applicable regulatory requirements.

**Ethics committee:** An independent body, constituted of medical professionals and non medical members whose responsibility is to verify that the safety, integrity and human rights of the subjects participating in a particular trial are protected and to consider the general ethics of the trial, thereby providing public reassurance.

**Good Clinical Practice (GCP):** A standard for clinical studies which encompasses the design, conduct, monitoring, termination, audit, analyses, reporting and documentation of the studies and which ensures that the studies are scientifically and ethically sound and that the clinical properties of the pharmaceutical products are properly documented.

**Informed consent:** A subject’s voluntary confirmation of willingness to participate in a particular trial and the documentation thereof after all appropriate information has been given about the trial. In the case of small children, the informed consent will be signed by the parent or guardian.

**Investigator:** Each medical person who is involved in the study conduct, and responsible for the trial and for the rights, health and welfare of the subjects in the trial.

**Monitor:** A person, appointed by and responsible to the, the sponsor for the monitoring and reporting of progress of the trial and for verification of data.

**Principal Investigator:** The investigator serving as coordinator within each study site.

**Protocol:** A document which states the background, rationale and objectives of the trial and describes its design, methodology and organisation.

**Source data:** All records of certified copies of original observations, clinical findings or other activities in a clinical trial necessary for the reconstruction and evaluation of the trial.

**Sponsor:** An individual, a company, an institution or an organisation which takes responsibility for the initiation, management and / or financing of a clinical trial.

**APPENDIX 8.**

**World Medical Association Declaration of Helsinki**

**WORLD MEDICAL ASSOCIATION DECLARATION OF HELSINKI**

**Ethical Principles for Medical Research Involving Human Subjects**

**Recommendations guiding medical physicians**

**in biomedical research involving human subjects**

Adopted by the 18th WMA General Assembly

Helsinki, Finland, June 1964

and amended by the

29th WMA General Assembly, Tokyo, Japan, October 1975

35th WMA General Assembly, Venice, Italy, October 1983

41st WMA General Assembly, Hong Kong, September 1989

48th WMA General Assembly, Somerset West, Republic of South Africa, October 1996

and the

52nd WMA General Assembly, Edinburgh, Scotland, October 2000

Note of Clarification on Paragraph 29 added by the WMA General Assembly, Washington 2002.

**A. INTRODUCTION**

1. The World Medical Association has developed the Declaration of Helsinki as a statement of ethical principles to provide guidance to physicians and other participants in medical research involving human subjects. Medical research involving human subjects includes research on identifiable human material or identifiable data.

2. It is the duty of the physician to promote and safeguard the health of the people. The physician's knowledge and conscience are dedicated to the fulfillment of this duty.

3. The Declaration of Geneva of the World Medical Association binds the physician with the words, "The health of my subject will be my first consideration," and the International Code of Medical Ethics declares that, "A physician shall act only in the subject's interest when providing medical care which might have the effect of weakening the physical and mental condition of the subject."

4. Medical progress is based on research which ultimately must rest in part on experimentation involving human subjects.

5. In medical research on human subjects, considerations related to the well-being of the human subject should take precedence over the interests of science and society.

6. The primary purpose of medical research involving human subjects is to improve prophylactic, diagnostic and therapeutic procedures and the understanding of the etiology and pathogenesis of disease. Even the best proven prophylactic, diagnostic, and therapeutic methods must continuously be challenged through research for their effectiveness, efficiency, accessibility and quality.

7. In current medical practice and in medical research, most prophylactic, diagnostic and therapeutic procedures involve risks and burdens.

8. Medical research is subject to ethical standards that promote respect for all human beings and protect their health and rights. Some research populations are vulnerable and need special protection. The particular needs of the economically and medically disadvantaged must be recognized. Special attention is also required for those who cannot give or refuse consent for themselves, for those who may be subject to giving consent under duress, for those who will not benefit personally from the research and for those for whom the research is combined with care.

9. Research Investigators should be aware of the ethical, legal and regulatory requirements for research on human subjects in their own countries as well as applicable international requirements. No national ethical, legal or regulatory requirement should be allowed to reduce or eliminate any of the protections for human subjects set forth in this Declaration.

**B. BASIC PRINCIPLES FOR ALL MEDICAL RESEARCH**

10. It is the duty of the physician in medical research to protect the life, health, privacy, and dignity of the human subject.

11. Medical research involving human subjects must conform to generally accepted scientific principles, be based on a thorough knowledge of the scientific literature, other relevant sources of information, and on adequate laboratory and, where appropriate, animal experimentation.

12. Appropriate caution must be exercised in the conduct of research which may affect the environment, and the welfare of animals used for research must be respected.

13. The design and performance of each experimental procedure involving human subjects should be clearly formulated in an experimental protocol. This protocol should be submitted for consideration, comment, guidance, and where appropriate, approval to a specially appointed ethical review committee, which must be independent of the Investigator, the Sponsor or any other kind of undue influence. This independent committee should be in conformity with the laws and regulations of the country in which the research experiment is performed. The committee has the right to monitor ongoing trials. The researcher has the obligation to provide monitoring information to the committee, especially any serious adverse events. The researcher should also submit to the committee, for review, information regarding funding, Sponsors, institutional affiliations, other potential conflicts of interest and incentives for subjects.

14. The research protocol should always contain a statement of the ethical considerations involved and should indicate that there is compliance with the principles enunciated in this Declaration.

15. Medical research involving human subjects should be conducted only by scientifically qualified persons and under the supervision of a clinically competent medical person. The responsibility for the human subject must always rest with a medically qualified person and never rest on the subject of the research, even though the subject has given consent.

16. Every medical research project involving human subjects should be preceded by careful assessment of predictable risks and burdens in comparison with foreseeable benefits to the subject or to others. This does not preclude the participation of healthy volunteers in medical research. The design of all studies should be publicly available.

17. Physicians should abstain from engaging in research projects involving human subjects unless they are confident that the risks involved have been adequately assessed and can be satisfactorily managed. Physicians should cease any investigation if the risks are found to outweigh the potential benefits or if there is conclusive proof of positive and beneficial results.

18. Medical research involving human subjects should only be conducted if the importance of the objective outweighs the inherent risks and burdens to the subject. This is especially important when the human subjects are healthy volunteers.

19. Medical research is only justified if there is a reasonable likelihood that the populations in which the research is carried out stand to benefit from the results of the research.

20. The subjects must be volunteers and informed participants in the research project.

21. The right of research subjects to safeguard their integrity must always be respected. Every precaution should be taken to respect the privacy of the subject, the confidentiality of the subject's information and to minimize the impact of the study on the subject's physical and mental integrity and on the personality of the subject.

22. In any research on human beings, each potential subject must be adequately informed of the aims, methods, sources of funding, any possible conflicts of interest, institutional affiliations of the researcher, the anticipated benefits and potential risks of the study and the discomfort it may entail. The subject should be informed of the right to abstain from participation in the study or to withdraw consent to participate at any time without reprisal. After ensuring that the subject has understood the information, the physician should then obtain the subject's freely-given informed consent, preferably in writing. If the consent cannot be obtained in writing, the non-written consent must be formally documented and witnessed.

23. When obtaining informed consent for the research project the physician should be particularly cautious if the subject is in a dependent relationship with the physician or may consent under duress. In that case the informed consent should be obtained by a well-informed physician who is not engaged in the investigation and who is completely independent of this relationship.

24. For a research subject who is legally incompetent, physically or mentally incapable of giving consent or is a legally incompetent minor, the Investigator must obtain informed consent from the legally authorized representative in accordance with applicable law. These groups should not be included in research unless the research is necessary to promote the health of the population represented and this research cannot instead be performed on legally competent persons.

25. When a subject deemed legally incompetent, such as a minor child, is able to give assent to decisions about participation in research, the Investigator must obtain that assent in addition to the consent of the legally authorized representative.

26. Research on individuals from whom it is not possible to obtain consent, including proxy or advance consent, should be done only if the physical/mental condition that prevents obtaining informed consent is a necessary characteristic of the research population. The specific reasons for involving research subjects with a condition that renders them unable to give informed consent should be stated in the experimental protocol for consideration and approval of the review committee. The protocol should state that consent to remain in the research should be obtained as soon as possible from the individual or a legally authorized surrogate.

27. Both authors and publishers have ethical obligations. In publication of the results of research, the Investigators are obliged to preserve the accuracy of the results. Negative as well as positive results should be published or otherwise publicly available. Sources of funding, institutional affiliations and any possible conflicts of interest should be declared in the publication. Reports of experimentation not in accordance with the principles laid down in this Declaration should not be accepted for publication.

**C. ADDITIONAL PRINCIPLES FOR MEDICAL RESEARCH COMBINED WITH MEDICAL CARE**

28. The physician may combine medical research with medical care, only to the extent that the research is justified by its potential prophylactic, diagnostic or therapeutic value. When medical research is combined with medical care, additional standards apply to protect the subjects who are research subjects.

29. The benefits, risks, burdens and effectiveness of a new method should be tested against those of the best current prophylactic, diagnostic, and therapeutic methods. This does not exclude the use of placebo, or no treatment, in studies where no proven prophylactic, diagnostic or therapeutic method exists.

30. At the conclusion of the study, every subject entered into the study should be assured of access to the best proven prophylactic, diagnostic and therapeutic methods identified by the study.

31. The physician should fully inform the subject which aspects of the care are related to the research. The refusal of a subject to participate in a study must never interfere with the subject-physician relationship.

32. In the treatment of a subject, where proven prophylactic, diagnostic and therapeutic methods do not exist or have been ineffective, the physician, with informed consent from the subject, must be free to use unproven or new prophylactic, diagnostic and therapeutic measures, if in the physician's judgment it offers hope of saving life, re-establishing health or alleviating suffering. Where possible, these measures should be made the object of research, designed to evaluate their safety and efficacy. In all cases, new information should be recorded and, where appropriate, published. The other relevant guidelines of this Declaration should be followed.

**FOOTNOTE:** **Note of Clarification on Paragraph 29 of the WMA Declaration of Helsinki**

The WMA hereby reaffirms its position that extreme care must be taken in making use of a placebo-controlled trial and that in general this methodology should only be used in the absence of existing proven therapy. However, a placebo-controlled trial may be ethically acceptable, even if proven therapy is available, under the following circumstances:

Where for compelling and scientifically sound methodological reasons its use is necessary to determine the efficacy or safety of a prophylactic, diagnostic or therapeutic method; or

Where a prophylactic, diagnostic or therapeutic method is being investigated for a minor condition and the patients who receive placebo will not be subject to any additional risk of serious or irreversible harm.

**APPENDIX 9**

**Flow of patients in OPD and Study Clinic**

Does the patient have an assigned treatment?

Diagnosis of malaria

Assess severity of disease

Complicated malaria

Record the information in a study registry: date and reason of visit

Standard treatment with quinine. Inform the study staff and record data required for visit 1 of CRF

Is the study clinic open? (8am - 5pm)

Inform the study staff

Record data required for visit 1 of CRF. CRF and drugs allocated to the study must be available in OPD

Refer to study clinic for management

Uncomplicated malaria

Yes

Yes

No

No

No

Management per clinic physician

No

Yes

Diagnosis of malaria

Is the study clinic open? (8am - 5pm)

No

Treat with standard care.

Yes

Yes
