# Supplementary material for: Efficacy and Safety of Fixed-Dose Artesunate-Amodiaquine vs. Artemether-Lumefantrine for Repeated Treatment of Uncomplicated Malaria in Ugandan Children
Source: PLoS One. 2014 Dec 1;9(12):e113311. doi: 10.1371/journal.pone.0113311 (PMC4249977; doi:10.1371/journal.pone.0113311)
Supplement: Approval S1 — Makerere Institutional Review Board/Ethics Committee approval document. (PDF) [file pone.0113311.s001.pdf]

MAKERERE UNIVERSITY

P. O. Box 7072 Kampala, Uganda
E-mail: dean@med.mak.ac.ug

Tel: +256-414-530020
Fax: +256-414-541036/0414-532204

WE BUILD FOR THE FUTURE

FACULTY OF MEDICINE
OFFICE OF THE DEAN

Tuesday, March 04, 2008

Dr. Ambrose Talisuna
Uganda Malaria Surveillance Project

Dear Dr. Talisuna

Re: **Approval of Proposal**

Your proposal entitled "**A randomized study to compare a fixed dose combination of Artesunate Plus Amodiaquine Versus A fixed Dose Combination of Artemether plus Lumentrine In treatment of Treatment of Repeated Uncomplicated Plasmodium Falciparum Malaria Attacks Occurring During Two Years of Follow-up, In Children in Uganda**" was considered and reviewed by the research and ethics Committee. The proposal was found to be good but some amendments were suggested for incorporation in order to improve on the science of the study. I am happy the suggested changes have been incorporated.

On behalf of the committee, I am glad to inform you that the proposal has been approved. You may now proceed with the process of data collection.

Please Note:

1. You are reminded that Principal Investigator is responsible for reporting any serious adverse events, requesting prior IRB (Institution Review Board) approval for all modifications, requesting continuing review and approval, and only using current IRB approved documents. You are also required to provide progress reports at regular intervals.
2. Final approval is to be granted by Uganda National Council of Science and Technology.

Yours sincerely,

Dr. Charles Ibingira
Chair, Research & Ethics Committee
