# Supplementary material for: Efficacy and Safety of Fixed-Dose Artesunate-Amodiaquine vs. Artemether-Lumefantrine for Repeated Treatment of Uncomplicated Malaria in Ugandan Children
Source: PLoS One. 2014 Dec 1;9(12):e113311. doi: 10.1371/journal.pone.0113311 (PMC4249977; doi:10.1371/journal.pone.0113311)
Supplement: Approval S2 — Uganda National Council of Science and Technology approval document. (PDF) [file pone.0113311.s002.pdf]

Uganda National Council For Science and Technology

(Established by Act of Parliament of the Republic of Uganda)

UNCST

Your Ref:..........................

Our Ref: HS 415 — ***Date:***........10/04/08........

Dr. Ambrose Otau Talisuna
Assistant Commissioner Health Services
Epidemiological Surveillance Division
Ministry of Health
Kampala

Dear Dr. Talisuna,

RE: **RESEARCH PROJECT APPROVAL AND CLEARANCE**

This is to inform you that on **March 19, 2008** the Uganda National Council for Science and Technology (UNCST) approved the research proposal entitled, **"A RANDOMIZED STUDY TO COMPARE A FIXED DOSE COMBINATION OF ARTESUNATE PLUS AMODIAQUINE VESUS A FIXED DOSE COMBINATION OF ARTHEMETHER PLUS LUMENFANTRINE IN TREATMENT OF REPEATED UNCOMPLICATED PLASMODIUM FALCIPARUM MALARIA ATTACKS OCCURRING DURING 2 YEARS OF FOLLOW-UP, IN CHILDREN IN UGANDA"**. The approval is valid until **March 19, 2009**. If it is necessary to continue with the research project beyond the expiry date, a request for continuation should be made in writing, and a progress report should be provided, to the Executive Secretary, UNCST.

Any problems of a serious nature related to the execution of your research project should be brought to the attention of the UNCST, and any changes to the research protocol should not be implemented without UNCST's approval except when necessary to eliminate apparent immediate hazards to the research participant(s).

This letter also serves as proof of UNCST approval and as a reminder for you to submit to UNCST timely progress reports and a final report on completion of the research project.

Please, note that you are required to obtain a certificate for the importation and/or use of the study drug(s) from the National Drug Authority.

Yours sincerely,

Leah Nawegulo
for: Executive Secretary
UGANDA NATIONAL COUNCIL FOR SCIENCE AND TECHNOLOGY

cc Executive Director, National Drug Authority
cc Resident District Commissioner, Tororo District

LOCATION/CORRESPONDENCE

Plot 3/5/7, Nasser Road
P.O. Box 6884
[illegible]

COMMUNICATION

TEL: (256) 414-250499, (256) 414-705500
FAX: (256) 414-234579
E-MAIL: uncst@starcom.co.ug
